# Rapid brain tumor classification from sparse epigenomic data

**DOI:** 10.1038/s41591-024-03435-3

**Published:** 2025-02-28

**Authors:** Björn Brändl, Mara Steiger, Carolin Kubelt, Christian Rohrandt, Zhihan Zhu, Maximilian Evers, Gaojianyong Wang, Bernhard Schuldt, Ann-Kristin Afflerbach, Derek Wong, Amy Lum, Skarphedinn Halldorsson, Luna Djirackor, Henning Leske, Svetlana Magadeeva, Romualdas Smičius, Claudia Quedenau, Nils O. Schmidt, Ulrich Schüller, Einar O. Vik-Mo, Martin Proescholdt, Markus J. Riemenschneider, Gelareh Zadeh, Ole Ammerpohl, Stephen Yip, Michael Synowitz, Alena van Bömmel, Helene Kretzmer, Franz-Josef Müller

**Affiliations:** 1 Department of Psychiatry and Psychotherapy, Christian-Albrecht University of Kiel, Kiel, Germany; 2 Department of Genome Regulation, Max Planck Institute for Molecular Genetics, Berlin, Germany; 3 Digital Health Cluster, Hasso Plattner Institute for Digital Engineering, Digital Engineering Faculty, University of Potsdam, Potsdam, Germany; 4 Department of Mathematics and Computer Science, Free University Berlin, Berlin, Germany; 5 Department of Neurosurgery, University Medical Center Schleswig-Holstein (UKSH), Campus Kiel, Kiel, Germany; 6 Altona Diagnostics GmbH, Hamburg, Germany; 7 Institute for Biology and Biotechnology of Plants, University of Münster, Münster, Germany; 8 Mathematische Modellierung, Entwicklung und Beratung, Düsseldorf, Germany; 9 Institute for Tumor Biology, University Medical Center Hamburg-Eppendorf, Hamburg, Germany; 10 Molecular Oncology, BC Cancer, Vancouver, British Columbia Canada; 11 Division of Genomic Diagnostics, Department of Pathology and Laboratory Medicine, Children’s Hospital of Philadelphia, Philadelphia, PA USA; 12 Department of Pathology and Laboratory Medicine, Faculty of Medicine, University of British Columbia, Vancouver, British Columbia Canada; 13 Vilhelm Magnus Laboratory for Neurosurgical Research, Institute for Surgical Research/Department of Neurosurgery, Oslo University Hospital, Oslo, Norway; 14 Section of Neuropathology, Department of Pathology, Oslo University Hospital, Oslo, Norway; 15 Max Delbrück Center for Molecular Medicine in the Helmholtz Association (MDC), Berlin, Germany; 16 Department of Neurosurgery, University Medical Center Regensburg, Regensburg, Germany; 17 Brain Tumor Center, University Medical Center Regensburg, Regensburg, Germany; 18 Department of Pediatric Hematology and Oncology, University Medical Center Hamburg-Eppendorf, Hamburg, Germany; 19 Research Institute Children’s Cancer Center Hamburg, Hamburg, Germany; 20 Institute of Neuropathology, University Medical Center Hamburg-Eppendorf, Hamburg, Germany; 21 Institute for Clinical Medicine, Faculty of Medicine, University of Oslo, Oslo, Norway; 22 Department of Neuropathology, Regensburg University Hospital, Regensburg, Germany; 23 MacFeeters Hamilton Neuro-Oncology Program, Princess Margaret Cancer Centre, University Health Network and University of Toronto, Toronto, Ontario Canada; 24 Division of Neurosurgery, Department of Surgery, University of Toronto, Toronto, Ontario Canada; 25 Princess Margaret Cancer Centre, University Health Network, Toronto, Ontario Canada; 26 Institute for Human Genetics, Ulm University and Ulm University Medical Center, Ulm, Germany; 27 Department of Computational Molecular Biology, Max Planck Institute for Molecular Genetics, Berlin, Germany; 28 Hoffmann Group, Leibniz Institute on Aging - Fritz Lipmann Institute (FLI), Jena, Germany

**Keywords:** Computational models, DNA sequencing, Machine learning, CNS cancer

## Abstract

Although the intraoperative molecular diagnosis of the approximately 100 known brain tumor entities described to date has been a goal of neuropathology for the past decade, achieving this within a clinically relevant timeframe of under 1 h after biopsy collection remains elusive. Advances in third-generation sequencing have brought this goal closer, but established machine learning techniques rely on computationally intensive methods, making them impractical for live diagnostic workflows in clinical applications. Here we present MethyLYZR, a naive Bayesian framework enabling fully tractable, live classification of cancer epigenomes. For evaluation, we used nanopore sequencing to classify over 200 brain tumor samples, including 10 sequenced in a clinical setting next to the operating room, achieving highly accurate results within 15 min of sequencing. MethyLYZR can be run in parallel with an ongoing nanopore experiment with negligible computational overhead. Therefore, the only limiting factors for even faster time to results are DNA extraction time and the nanopore sequencer’s maximum parallel throughput. Although more evidence from prospective studies is needed, our study suggests the potential applicability of MethyLYZR for live molecular classification of nervous system malignancies using nanopore sequencing not only for the neurosurgical intraoperative use case but also for other oncologic indications and the classification of tumors from cell-free DNA in liquid biopsies.

## Abstract (web summary)

MethyLYZR, an epigenetic classifier of brain tumors, provides clinically relevant cancer classification results within 15 min of sequencing, with potential applications for neurosurgical intraoperative use.

## Main

Intraoperative diagnostic procedures in oncologic surgery date back to the late 19th century and have substantially impacted patient outcomes^1^. They serve two primary clinical purposes: first, to establish a pathologic diagnosis, and second, to evaluate tumor cells at the resection margins^1^. The most immediate intraoperative use case is to differentiate surgical tumors from those for which non-surgical treatment modalities are preferable^2,3^. The increasing reliance of modern neuropathology on molecularly and specifically epigenetically defined tumor classes is exemplified by the most recent edition of the World Health Organization (WHO) classification of central nervous system (CNS) tumors^4^. It is based, in part, on the fundamental insight that malignancies found in the CNS can be identified and grouped into tumor classes based on genome-wide methylation profiles^5^. Specifically, a method developed by Capper et al. using a random forest model for methylation microarrays^5,6^ enables, today, the classification of up to 184 CNS tumor categories (DKFZ Brain Classifier 12.8) and has been integrated into clinical practice^7–9^. However, all genome-wide molecular methods currently employed in translational research and clinical routine require turnaround times of several days or, in some cases, weeks, precluding their use as next-day or even intraoperative diagnostic applications^8,10,11^.

Nanopore sequencing has become a transformative technology in preclinical research at the point of care (POC)^12^. Three specific features render this technology an attractive candidate for delivering molecular information within the timeframe of a neuro-oncologic surgery. First, nucleotide-resolution sequence data are available for further analysis and interpretation only milliseconds after a DNA or RNA strand enters a nanopore. Second, information on epigenetic modifications of these nucleotide sequences can be obtained within the same immediate timeframe. Third, transposase-based library preparation for nanopore sequencing experiments can be completed in minutes, enabling clinical sequencing workflows with a relatively small equipment footprint at the POC.

Several workflows employ nanopore sequencing to diagnose CNS tumors, sometimes within a day or even during neuro-oncologic surgery. Such diagnoses are achieved by classifying tumors according to characteristic CpG methylation profiles^8,13–15^. The initially proposed random forest approach has been customized to adaptive nanopore sequencing for a 4-day workflow^8^ and has been recently modified to also enable intraoperative applications^13,14^. This use case involves sample-specific, ad hoc training on only those CpGs covered in each nanopore sequencing experiment, typically necessitating 1.5 h (91–161 min) from sample to result^13,14^.

**Fig. 1:**
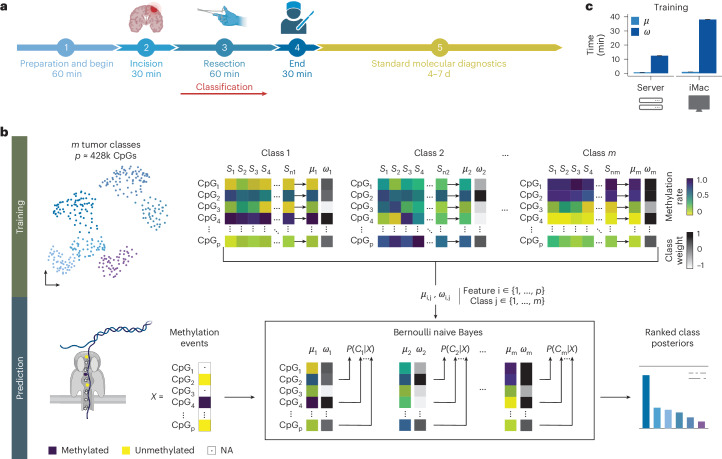
MethyLYZR enables tumor class prediction on sparse data without model retraining. **a**, Simplified schematic of the timeline of a brain surgery procedure. The stages encompass the following: (1) induction, involving anesthesia and patient positioning with neuronavigation adjustments (approximately 45–60 min); (2) incision and progression to the tumor (approximately 30 min); (3) tumor resection (approximately 60 min) and (4) retraction and completion of suturing (approximately 30 min). Notably, the 60-min tumor resection stage is the critical time window for obtaining a molecular diagnosis. However, the turnaround times of established molecular diagnostics extend beyond the length of the surgical procedure. **b**, Illustration of the training and prediction process of the naive Bayes algorithm. Multiple tumor classes (*m* classes) with several samples contribute CpG methylation ratios (*p* features) for algorithm training. The training involves generating *m* centroids ($\mu$) based on the provided samples (${S}_{1},\ldots ,{S}_{{n}_{m}}$), describing the average methylation probability of each of the *n* CpGs (features) per tumor class. Additionally, weights ($w$) are calculated per CpG and class, reflecting the predictive power of a CpG for a specific tumor class. For tumor class prediction in a given sample, sparse, binary methylation values from individual molecules—for example, obtained through Nanopore sequencing—serve as input for the pre-trained Bernoulli naive Bayes model. The output comprises a ranked list of posterior probabilities of all tumor classes in the model. **c**, Benchmarking analysis of MethyLYZR training time on published CNS 450k methylation arrays across 91 tumor classes with a total of 2,801 samples^5^. The training was executed on a single core using a Dell PowerEdge R7525 server (3 GHz AMD 64-Core Processor, 256 CPUs, 1,031.3 GB DDR4 RAM, Linux distribution) and an Apple iMac Pro (3 GHz 10-Core Intel Xeon W, 64 GB 2,666 MHz DDR4 RAM, 1 TB APFS SSD, Radeon Pro Vega 56 GPU with 8 GB VRAM, macOS 13.2.1). Notably, centroids and weight training were achieved on the server in under 20 min and on the iMac Pro in under 40 min.

Sample-to-result time and clinically relevant diagnostic accuracy are the primary concerns for any intraoperative diagnostic procedure. Although a typical CNS tumor resection requires a median time of 3 h (179 min; 123–250 min)^16^, the decisive time after a neurosurgeon reaches a brain tumor and any diagnostic information from a biopsy could realistically influence the extent of a subsequent resection is limited to under 1 h (Fig. 1a). Although imaging-based, stimulated Raman histology has demonstrated sample-to-result times of less than 2.5 min, the underlying neural networks currently identify substantially fewer tumor classes (*n* = 13) compared to those distinguishable using an integrated molecular approach (*n* = 108)^3,4^.

Most recently, the application of neural network models to nanopore data has yielded predictions of similar accuracy to an ad hoc random forest classifier within seconds, demonstrating a practicable turnaround time of approximately 1.25 h from sample to result^15^. However, due to the limited amount of publicly available training data, deep learning necessitates the simulation of tens of millions of nanopore datasets to train and validate the complex classifiers while demanding extensive computational resources for hyperparameter tuning.

Here we present MethyLYZR, a probabilistic framework that enables live classification of malignant transformed tissues from sparse DNA methylation profiles without requiring ad hoc training. MethyLYZR results are similar and, in many cases, superior in diagnostic accuracy to competing methods.

## Results

Nanopore sequencing is a stochastic ‘shotgun’ sequencing approach^17^. Despite its potential for high-throughput scaling^18^, it can realistically capture only a small portion of the human genome, typically well below 2%, within the critical timeframe of neurosurgical oncology procedures. In this context, unlike methylation arrays or deep sequencing datasets, shallow nanopore sequencing provides a single-molecule, binary output regarding a CpG’s methylation status. Each CpG site on a single DNA molecule is classified as methylated or unmethylated, diverging from the continuous, bulk methylation measurements (methylation rate or probability) typically obtained via methylation arrays. Another major challenge is the stochastically obtained feature set—every sequencing experiment will recover a different, random subset of CpGs.

These specific constraints render the Bernoulli naive Bayes classifier^19,20^ a suitable framework to address the unique algorithmic challenges of classifying cancer epigenomes in the shortest possible time. The classifier uses Bayes’ theorem to update the likelihood that a tumor sample belongs to a particular cancer class as new methylation data come in (Fig. 1b).

**Extended Data Fig. 1:**
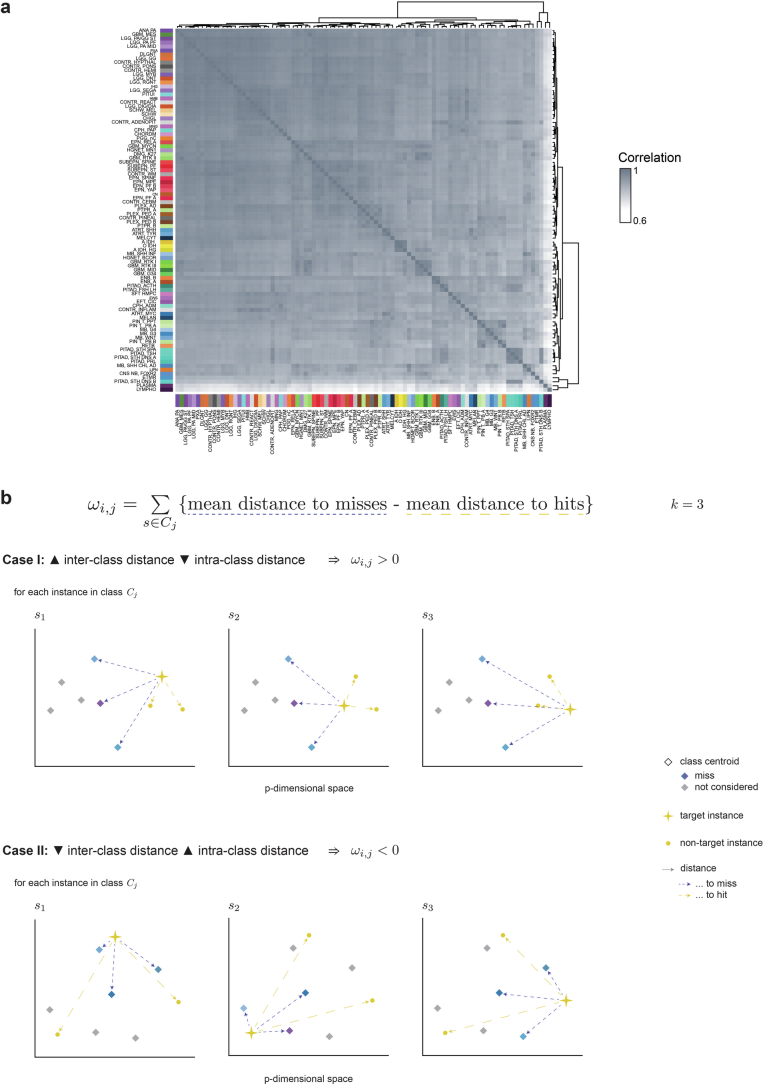
Training of weights for Naïve Bayes classifier. **a**) Correlation matrix of centroids derived from the training of MethyLYZR on 2,801 CNS 450k methylation arrays, segregated into 91 classes. Utilizing all CpGs post-quality control, the majority of centroids exhibit high correlation. **b**) Schematic of ReliefF-algorithm-based calculation of feature weights. Weights ω_ij_ are calculated for each feature (CpG) and class with index i and j. In brief, for every sample within a class ${C}_{j}$, the mean distance to its k-nearest foreign centroids (misses: inter-class) is calculated and subtracted by the mean distance to all other samples within the same class (hits: inter-class). The proximity of samples and centroids is pre-calculated on the full, p-dimensional information. CpGs that serve as good and precise predictors for a class will exhibit a smaller intra-class than inter-class distance, resulting in a positive weight, and vice versa for non-specific CpGs.

To train the Bernoulli naive Bayes classifier, we calculate the average methylation rate for each CpG site across different cancer classes, using data from the Illumina 450k methylation arrays. This gives us a probability of methylation for each CpG site within each cancer class (Fig. 1b, top). MethyLYZR then applies a weighting system^21,22^ to these probabilities to enhance its accuracy, particularly in distinguishing between closely related cancer types. This system also accounts for the fact that methylation patterns at different CpG sites are often correlated, which helps to improve the model’s reliability^23–25^ (Methods; Supplementary Fig. 1; Fig. 1b top; and Extended Data Fig. 1).

For the actual cancer classification, the naive Bayes classifier updates its predictions about the likely tumor type as new methylation data from the nanopore sequencing become available (Fig. 1b, bottom). It generates a list of possible tumor classes with the most probable class identified as the most likely result.

Of note, a central property of the naive Bayes classifier is its ability to accurately predict tumor types, even when only a random subset of CpG sites is available. Although missing values are a major challenge for most other machine learning approaches, they are intrinsically easy to deal with when employing a naive Bayes model: as long as the measurements are missing at random, ‘one simply ignores them’^26^.

Taken together, in the context of low-coverage nanopore sequencing with more than 98% of missing observations, the Bernoulli naive Bayes classifier is particularly well suited for intraoperative classification.

**Extended Data Fig. 2:**
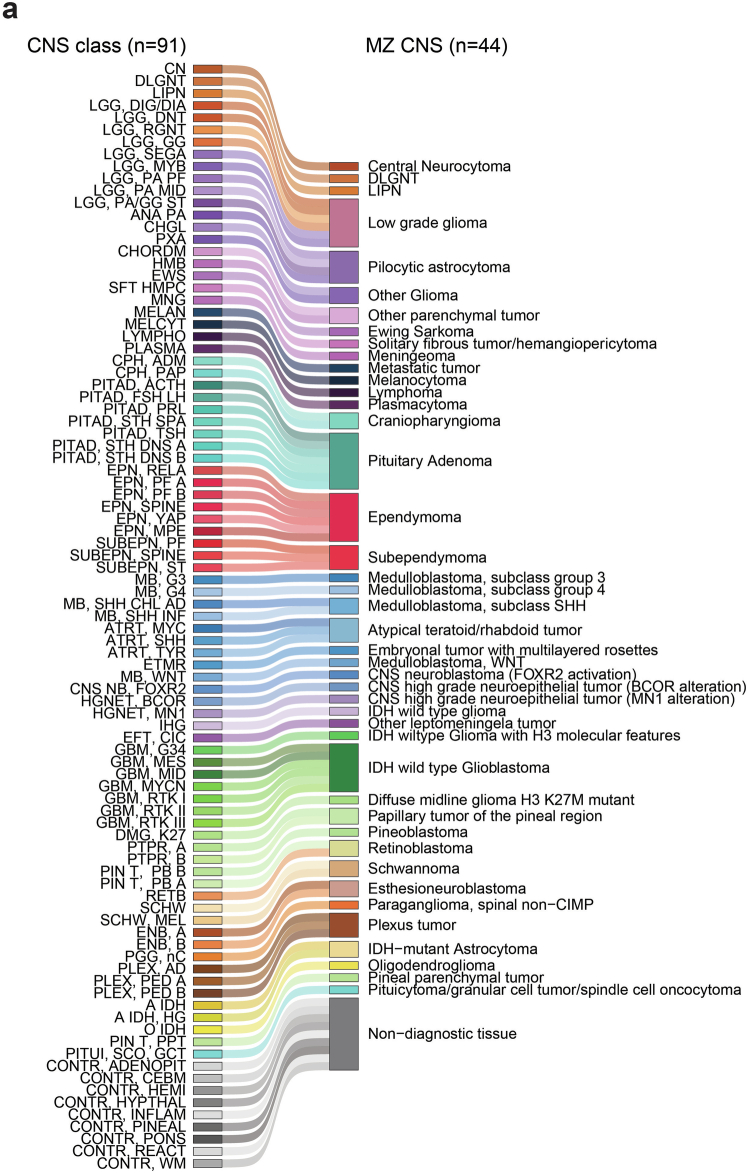
Grouping of 91 CNS classes into 44 clinically relevant MZ CNS classes. **a**) 91 CNS tumor classes are consolidated into 44 MZ CNS classes, preserving clinically relevant distinctions. Larger fusions are particularly impactful on Low-grade glioma, Pituitary Adenoma, Ependymoma, IDH wild-type Glioblastoma, and non-diagnostic control classes.

In the absence of extensive methylation sequencing references for most brain tumor types, we used a publicly available 450k methylation array atlas with 2,801 samples across 91 CNS tumor and control classes for training^5^. This dataset was previously used to train random forest and neural network algorithms for intraoperative classification tasks^13–15^. The 91 class labels in the training dataset represent a combination of CNS tumor entities, suggestive grading information and molecular concepts and, in some instances, reflect computationally derived sample groups with unknown clinical significance^5^. For practical application, we reordered the 91 CNS training classes into 44 MethyLYZR (MZ) CNS classes, guided by their potential clinical impact (Extended Data Fig. 2a, Supplementary Table 1 and Supplementary Text) as well as eight broad methylation class families (MCFs) as outlined previously^5^. For example, we consolidated six glioblastoma subtypes identified in the training dataset^5^ to reflect the clinical reality where such specific subtypes are not routinely distinguished during standard diagnostic procedures. Similarly, nine control tissues were categorized as ‘non-diagnostic tissue’, supporting the distinction between neoplastic tumors and non-malignant or diagnostically inconclusive tissue, which is relevant for clinical decision-making.

Training of MethyLYZR’s weighted naive Bayes algorithm is efficient and fast, with linear complexity in the number of features and quadratic complexity in the number of samples. This efficiency enables the algorithm to complete training while requiring minimal computational resources: within a few minutes on a high-performance server and in well less than 1 h on a 2017 Apple iMac personal computer (Fig. 1c, legend, and Supplementary Table 2).

**Extended Data Fig. 3:**
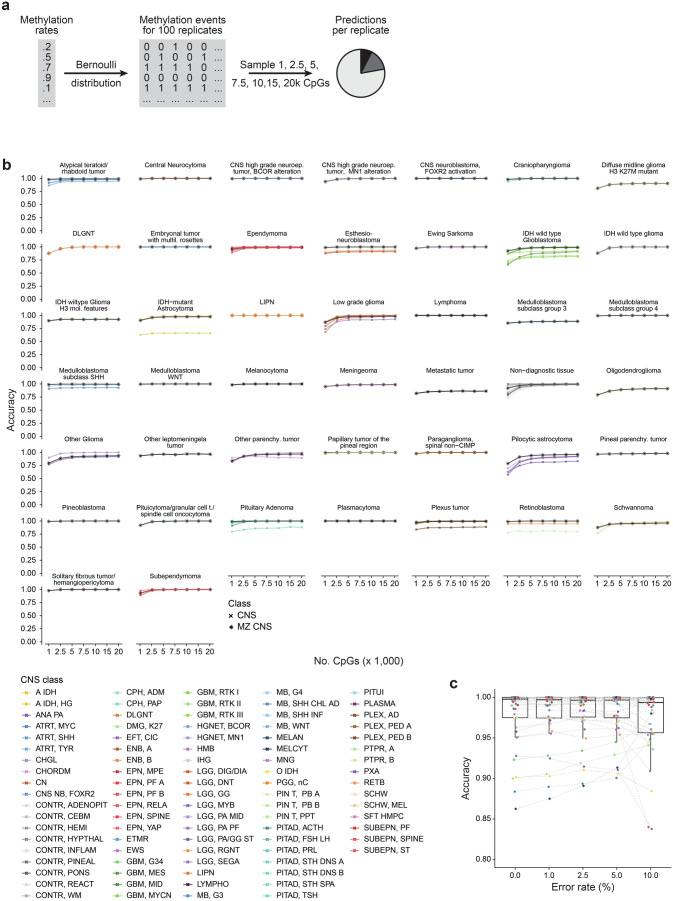
Evaluation of prediction accuracy per CNS tumor class. **a**) Schematic illustrating the simulation strategy for obtaining in silico converted binary methylation events from 450k arrays. Methylation rates per sample serve as the probability for a CpG to be methylated on a single molecule. Employing a Bernoulli distribution, the methylation rates of 100 molecules are simulated for each CpG, resulting in 100 sampled replicates from each 450k methylation array. Subsequently, a subset of CpGs (n = 1k, 2.5k, 5k, 7.5k, 10k, 15k, and 20k) is randomly sampled and utilized for class prediction of each synthetic replicate. **b**) Accuracy of predictions per CNS class as a function of the number of sampled CpGs. The 91 CNS classes are grouped by clinical relevance MZ CNS class. In the majority of classes, predictions exhibit improvement with an increasing number of CpGs, plateauing at around 5 to 7.5k CpGs. Beyond this threshold, further increments in the number of CpGs yield only marginal improvements in predictions. Black lines indicate the average accuracy of the MZ CNS class. **c**) Accuracy of predictions per MZ CNS class (n = 44) for different levels of error. The error rate indicates the frequency with which a CpG methylation status was inverted. The average accuracy is 97.89%, 98.01%, 98.10%, 98.08%, and 97.30% for error levels of 0%, 1%, 2.5%, 5%, and 10%, respectively. Boxplots display the median as the central line, the interquartile range (IQR; 25^th^ to 75^th^ percentile) as the box, and outliers (points beyond 1.5 times the IQR) as dots outside the whiskers.

For performance evaluation, we initially generated a synthetic dataset to simulate shallow nanopore methylation patterns based on the 450k methylation array reference (Extended Data Fig. 3a). This involved generating 100 replicates per sample for each of the 91 brain tumor classes, each providing binary methylation data for every CpG (280,100 synthetic samples in total).

**Fig. 2:**
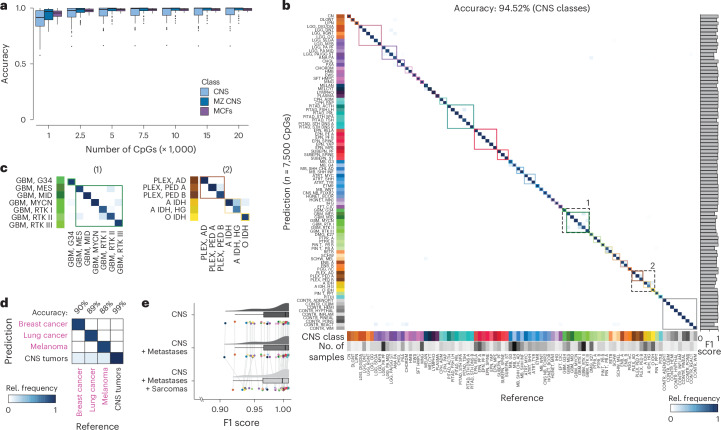
Highly accurate tumor class prediction from sparse, binary DNA methylation profiles based on 450k methylation arrays. **a**, Evaluation of prediction accuracy for the synthetic samples using a random subset of 1,000, 2,500, 5,000, 7,500, 10,000, 15,000 or 20,000 CpGs. In silico simulation of 100 × 2,801 samples mirroring low-coverage Nanopore sequencing was performed from 450k arrays of 2,801 biologically independent samples representing 91 CNS cancer and control methylation classes. Box plots display the median as the central line, the IQR (25th–75th percentile) as the box and outliers (points beyond 1.5× the IQR) as dots outside the whiskers. **b**, Confusion matrix depicting the prediction outcomes for all imputed samples using 7,500 CpGs, yielding an overall accuracy of 94.52% for CNS classes and 97.72% for MZ CNS classes. Colors indicate relative frequencies that are normalized to the number of samples in each reference class. Misclassification errors are represented by deviations from the bisecting line, and clinically relevant groups (MZ CNS classes) are highlighted by colored squares. F1 scores are provided on the right. **c**, Zoom into the confusion matrix for groups of CNS tumor classes with slightly lower F1 scores than the average. **d**, Confusion matrix illustrating predictions on an extended dataset, including CNS tumors, breast cancer, lung cancer and melanoma CNS metastases (91 CNS classes and 2,801 samples; three metastatic classes and 85 samples). Using 7,500 CpGs, MethyLYZR achieves an accuracy of 90.31%, 89.39%, 88.76% and 99.99% in distinguishing among breast, lung, melanoma and CNS samples, respectively. **e**, Distribution of F1 scores per class resulting from the prediction of 280,100 simulated CNS samples across three models with increasing complexity. The three models include 91 CNS classes (top), 91 CNS + 3 metastasis classes (middle) and 91 CNS + 3 metastasis + 64 sarcoma classes (bottom). F1 scores per model are represented as dots and summarized through box and density plots. Box plots display the median as the central line, the IQR (25th–75th percentile) as the box and outliers (points beyond 1.5× the IQR) as dots outside the whiskers.

**Extended Data Fig. 4:**
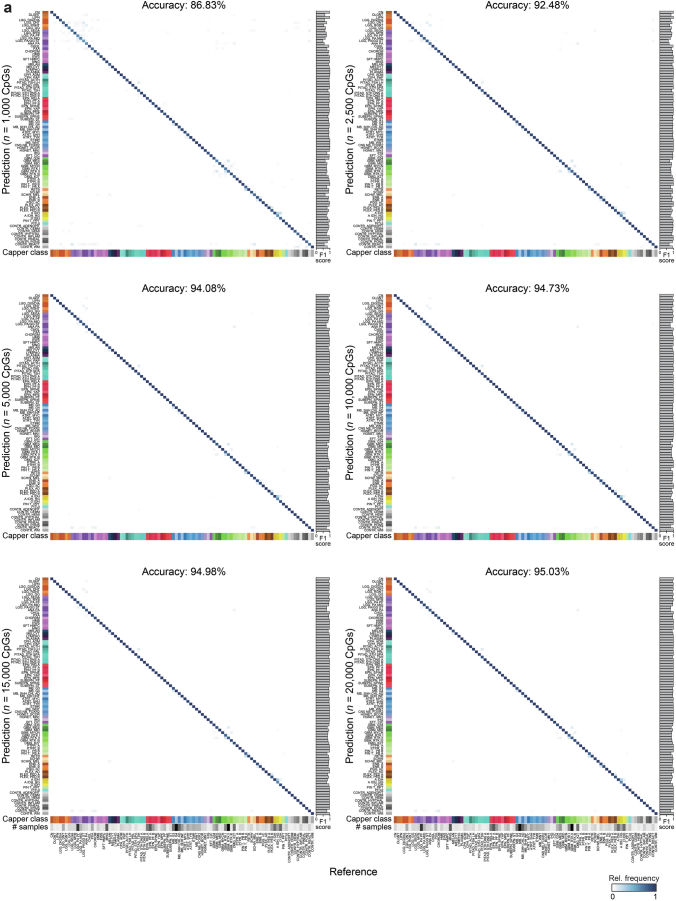
Evaluation of prediction accuracy per CNS tumor class. **a**) Confusion matrix depicting the prediction outcomes for all imputed samples using 1k, 2.5k, 5k, 7.5k, 10k, 15k, and 20k CpGs, yielding an overall accuracy of 86.83% to 95.03% for CNS classes and 92.42% to 97.99% for MZ CNS classes. Color key indicates relative frequencies that are normalized to the number of samples in each reference class. Misclassification errors are represented by deviations from the bisecting line and F1 scores per class are provided on the right.

To assess the impact of sequencing depth on accuracy, we sampled methylation data of 1 to 20,000 CpGs from synthetic nanopore profiles. Using only 1,000 randomly selected CpGs, this resulted in an overall median accuracy across classes of 91.45%, 97.02% and 95.47% across all 280,100 synthetic samples (0.2% of all modeled CpGs; CNS, MZ CNS and MCFs, respectively; Fig. 2a, Extended Data Fig. 3b and Supplementary Tables 3–5). Including an increasing number of CpGs results in improved accuracy, saturating at approximately 7,500 CpGs. At this number of CpGs, we observed an accuracy of 94.52% across all samples within the 91 CNS classes (Fig. 2b). Furthermore, when introducing methylation calling error rates of up to 10% in silico, the accuracies appeared to be stable (94.70%, 94.53%, 94.92% and 93.73% with error rates of 1%, 2.5%, 5% and 10%, respectively; Extended Data Fig. 3c). Notably, across all tested CpG quantities, most misclassifications were not random but confined to our broader diagnostic categories (97.72% accuracy on MZ CNS classes for 7,500 CpGs; Fig. 2a–c and Extended Data Figs. 3b and 4a).

Epidemiologically, intracranial metastases are estimated to be 10 times more common than primary brain tumors^27^. Consequently, neurosurgical biopsies for brain metastases are frequent and essential when neuroimaging is ambiguous, no primary tumor is known, multiple primaries exist or when specific tumor characteristics could influence treatment decisions^28^.

**Extended Data Fig. 5:**
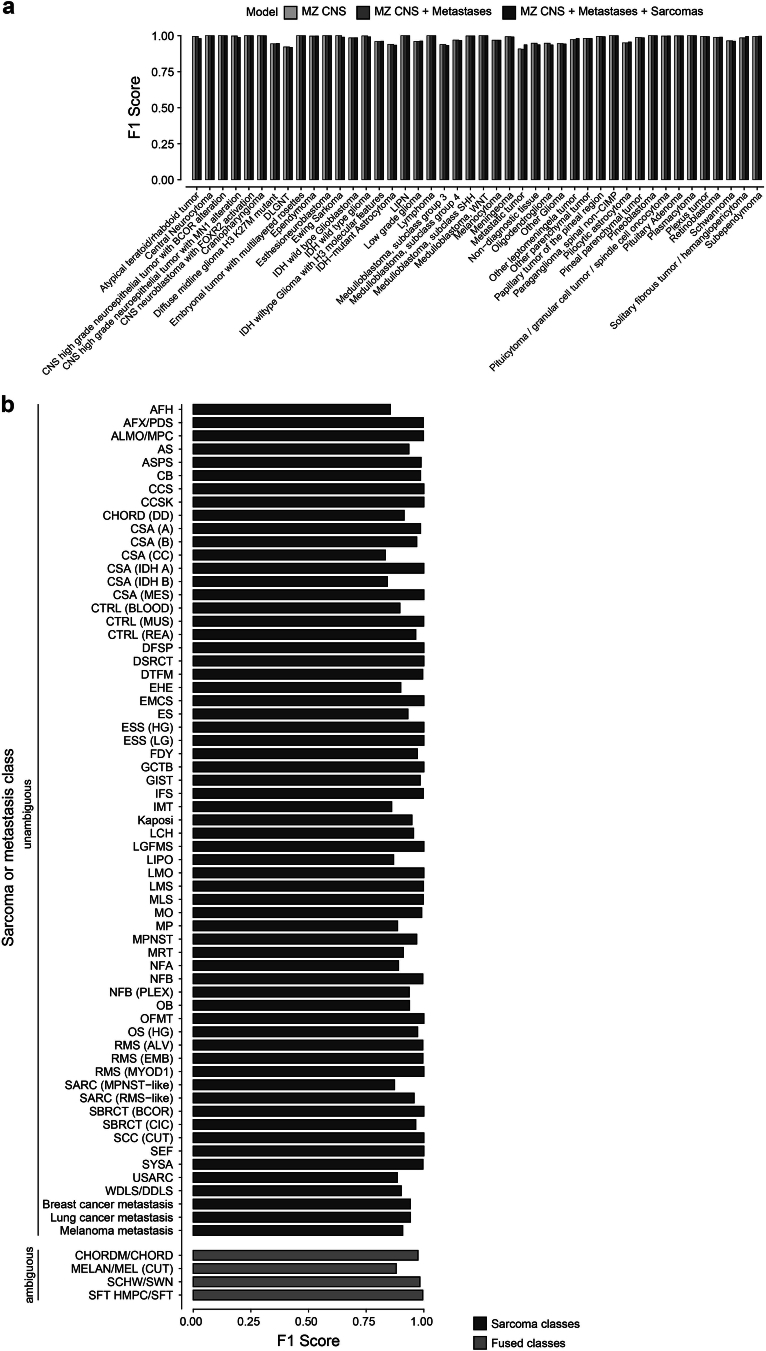
Evaluation of MethyLYZR on models with increased complexity. **a**) F1 scores per MZ CNS class resulting from the prediction of 100 ×2,801 simulated CNS samples across three models with increasing complexity. The F1 scores per MZ CNS class demonstrate no decline of F1 scores across the models, suggesting sustained accuracy even with substantially expanded model scopes. The three models include 91 CNS classes (light grey), 91 CNS + 3 metastasis classes (grey), and 91 CNS + 3 metastases + 64 sarcoma classes (dark grey). **b**) F1 scores for each sarcoma or metastases class derived from the prediction of 100 ×1,162 simulated samples using a model trained on 91 CNS, 3 metastases, and 64 sarcoma classes. All F1 scores surpass 0.83, with the majority approaching almost 1 (mean: 0.96). Classes that were present in more than one reference dataset were fused, labeled as ambiguous, and appropriately accounted for.

To expand the clinical utility of MethyLYZR and assess the impact of broadening its scope, we augmented the training dataset^5^ with additional tumor samples originating from breast cancer, lung cancer and melanoma CNS metastases^29^ (three metastatic classes, 85 samples). Testing the predictive power of MethyLYZR in this expanded model, we first retrained on CNS and metastasis samples and followed the above-outlined evaluation approach to generate synthetic, sparse datasets (Extended Data Fig. 3a). Notably, when including the metastatic classes, our model demonstrated the ability to differentiate between brain and metastatic tumor samples with 88.76% to 90% accuracy using randomly selected, synthetic subsets of 7,500 CpGs (Fig. 2d, Extended Data Fig. 5a and Supplementary Tables 6 and 7).

To further evaluate the adaptability of MethyLYZR, we expanded our training dataset to include sarcomas^30^ (64 classes represented by 1,077 samples), increasing the total to 158 classes. We then assessed the model’s performance on the original CNS samples to determine if the expansion to CNS and metastasis or CNS, metastasis and sarcoma impacted the predictive reliability. The statistical analysis of F1 scores (Wilcoxon test *P* value: 0.8339 and 0.2314, respectively) indicated that accuracy was maintained despite the substantially broader scope of the expanded model (Fig. 2e, Extended Data Fig. 5a,b and Supplementary Tables 4 and 7–9).

**Fig. 3:**
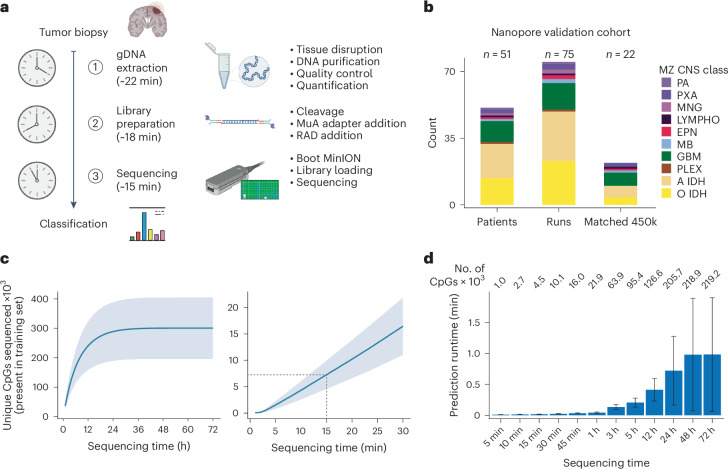
Workflow for intraoperative shallow Nanopore sequencing. **a**, Schematic representation of the timeline for intraoperative tumor sequencing and classification in our study. The cancer class prediction is achieved within a rapid turnaround time of just 1 h from tumor biopsy reception. The process involves genomic DNA extraction (approximately 22 min), Nanopore library preparation (approximately 18 min) and loading of the library with subsequent sequencing (15–20 min). **b**, Description of the Nanopore and 450k methylation array cohort derived from patients with CNS cancer in this study. A total of 75 Nanopore runs were conducted using samples from 51 patients, and, for a subset of 22 patients, 450k methylation arrays were generated from matched tumor biopsies. **c**, Relationship between sequencing time and the number of CpGs sequenced at least once, derived from our cohort of 75 Nanopore runs. In the initial 24 h of sequencing, the count of newly observed CpGs rises with sequencing time, saturating into enhanced coverage per CpG thereafter (left). Within 15 min of sequencing, approximately 7,500 CpGs are covered on average (right). Data are presented as mean ± s.d. **d**, Benchmarking analysis of MethyLYZR prediction time on our Nanopore runs using the model trained on the 91 CNS and three metastasis tumor classes executed on an Apple iMac Pro (3 GHz 10-Core Intel Xeon W, 64 GB 2,666 MHz DDR4 RAM, 1 TB APFS SSD, Radeon Pro Vega 56 GPU with 8 GB VRAM, macOS 13.2.1). For data acquired from 15 min of sequencing, the runtime is negligibly small (on average less than 1 s), and, even with full 72-h runs, the prediction time remains well below 4 min, even in the most extreme cases (on average less than 1 min). Numbers on top state the mean number of CpGs for each time benchmarked. The bar represents the median, and the error bar is the s.d. gDNA, genomic DNA.

To adapt our approach for intraoperative sequencing, we first optimized the library preparation strategy for intraoperative applications (Fig. 3a and Supplementary Video). Specifically, we refined a commercially available DNA preparation method to consistently extract DNA from brain tumor biopsies within 22 min. We next optimized a protocol for a transposase-based, rapid nanopore library preparation kit to obtain a sequencing library within 18 min. This protocol works with small tissue samples (10–15 mg) realistically obtainable during routine neurosurgical procedures, yielding sufficient DNA for nanopore sequencing (600–700 ng required for R9 and 100–150 ng for R10 pores, due to the increased sensitivity of R10) in parallel with biopsy retrieval for current clinical integrated diagnostic procedures. Furthermore, we adapted MethyLYZR into the standard Oxford Nanopore Technologies (ONT) basecalling workflow, establishing a live methylation processing pipeline. This end-to-end integration enables immediate POC diagnostic cancer prediction from CpG methylation data directly from the sequencer, without internet reliance. We can obtain sufficient methylation measurements from approximately 15–20 min of sequencing using our optimized workflow. This allows us to complete the process from biopsy acquisition to prediction in under 1 h (Fig. 3a).

Using our optimized strategy, which takes approximately 40 min for library preparation, we generated 75 nanopore separate sequencing experiments using a MinION sequencer and R9 flow cells from 51 patient biopsies (Fig. 3b and Supplementary Table 10). For this sample set, postoperative diagnoses were based on molecular markers and histopathological assessments by a university center neuropathologist. In line with previous classification studies, we grouped our samples into MZ CNS classes in view of the intraoperative practical application (Extended Data Fig. 2a). Our nanopore reference samples span 10 different brain tumor classes. For validation, we expanded the dataset by matching Illumina EPIC methylation arrays for 22 samples (Supplementary Table 11).

Overall, nanopore sequencing of these samples indicates a near-linear correlation between sequencing time and coverage of model features in the first hours, with saturation after approximately 24 h (Fig. 3c). Within the 15 min that our workflow allows for sequencing, we obtain 1,878–12,487 CpGs with a mean of approximately 7,500 CpGs (Supplementary Table 12). Given the results of the synthetic data above, we expect our protocol to enable robust and reliable live tumor diagnosis from sparse CpG methylation data. Because the tumor class prediction will run in parallel to an ongoing nanopore sequencing run, we additionally evaluated the time and memory requirements for prediction on an increasing number of CpGs (Fig. 3d). Notably, the computational costs, specifically in terms of time and memory, remain negligible even for full 72-h runs—on average requiring less than 1 min and less than 3 GB of RAM with more than 200,000 unique CpGs covered.

**Extended Data Fig. 6:**
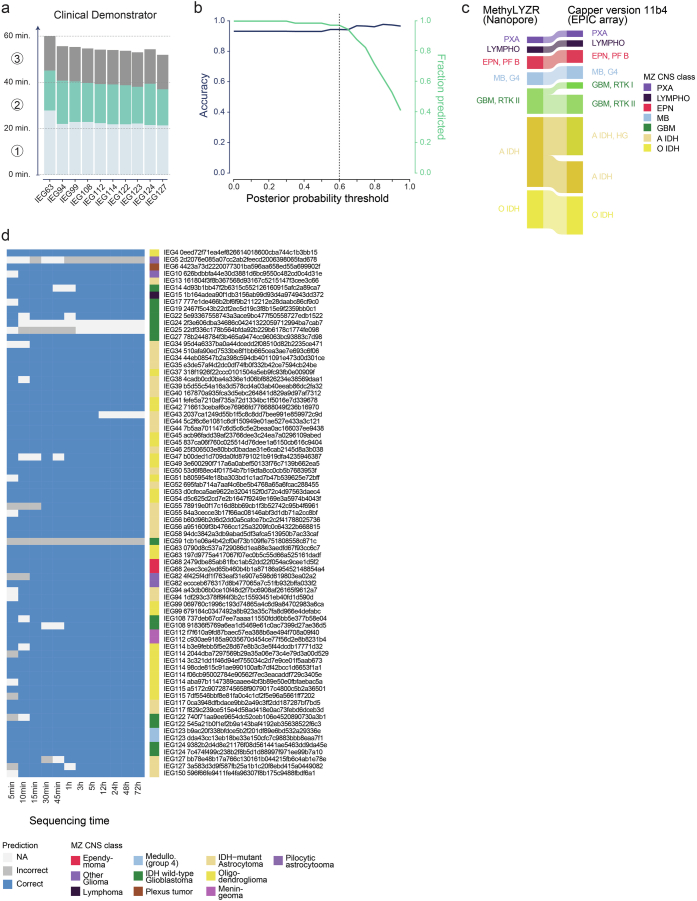
Evaluation of prediction accuracy on Nanopore samples. **a**) Comparison of times for (1) DNA isolation, (2) library preparation, and (3) sequencing for 10 samples run in interoperative settings in the clinics (‘Clinical Demonstrators’) with corresponding videos (see Supplementary Video). The median times are 22:15 minutes for DNA extraction, 17:10 minutes for library preparation, and predictions after 15 minutes of sequencing, resulting in the correct diagnosis in all runs (2-4 sequencing runs per sample to validate consistency in results, Supplementary Table 10). **b**) Assessment of a posterior probability cutoff for Nanopore samples. The graph illustrates the percentage of samples exceeding the threshold for each posterior probability, along with the corresponding accuracy based on these samples. Notably, a posterior probability of 0.6 or higher resulted in a high percentage of correctly predicted samples. **c**) Comparative analysis of predicted classes derived from Nanopore data using MethyLYZR and matching 450k methylation arrays employing the Capper classifier version 11b4. At the CNS class level, predictions align in 74.07% of cases, while 100% of predictions are in agreement at the clinically relevant MZ CNS levels. **d**) Assessment of predictive power across sequencing times ranging from 5 minutes to 72 hours, analyzed individually by runs. The most substantial increase in prediction power was observed between 5 and 15 minutes of sequencing, with three samples exhibiting either no correct predictions or none at all. The color bar on the side depicts the reference class.

For a subset of 10 samples, the entire workflow was run in an intraoperative setup (Supplementary Video). Given the stringent timeline of less than 1 h for clinical validation, each step—from surgical planning and biopsy handling to DNA extraction, nanopore sequencing and bioinformatics analysis—is tightly interconnected. The intraoperative process was preceded by setting up a tailored laboratory, the establishment of ethical, legal and scientific frameworks and specific surgical planning (see Methods ‘Clinical Demonstrator experimental workflow’ subsection). Time-critical intraoperative steps include the rapid extraction and sequencing of DNA from tumor biopsies, followed by the live application of the MethyLYZR algorithm, confirming our turnaround times of approximately 22 + 18 min until sequencing in the clinical environment (Extended Data Fig. 6a and Supplementary Table 10).

**Fig. 4:**
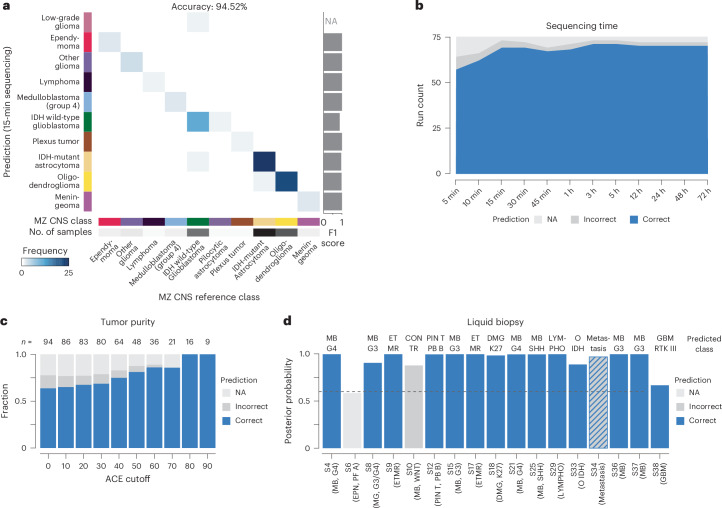
MethyLYZR predicts cancer classes from CNS cancer as well as spinal cord liquid biopsies with high accuracy. **a**, Confusion matrix illustrating the prediction outcomes for all Nanopore samples using CpGs obtained within 15 min of sequencing, resulting in an overall accuracy of 94.52% for MZ CNS classes. Misclassification errors are depicted by deviations from the bisecting line, and F1 scores per class are presented on the right. **b**, Evaluation of predictive power across sequencing times ranging from 5 min to 72 h. The largest increase in prediction accuracy was observed between 5 min and 15 min of sequencing (89.06% versus 94.52%). Beyond this interval, extended sequencing times yielded only small improvements in accuracy (94.52% versus 97.22% for 15 min versus 72 h). **c**, Tumor class predictions for 96 Nanopore-sequenced CNS tumors based on 7,500 CpGs to simulate 15 min of sequencing, stratified by estimated purity (ACE). As purity increases, the accuracy of MethyLYZR demonstrates an upward trend, reaching consistently high levels of diagnostic accuracy from approximately 60% tumor purity onward. Accuracy (%) left to right: 82.2, 84.8, 87.5, 87.3, 90.6, 92.6, 96.9, 100.0, 100.0 and 100.0. **d**, Tumor class predictions for 17 cfDNA samples obtained from CSF samples of pediatric CNS tumor patients with more than 2,500 CpGs covered and an estimated tumor fraction above 0.1. MethyLYZR provided high-confidence predictions for 16 of the 17 samples and, among these, achieved 93% accuracy, including a metastasis predicted as metastatic (instead of CNS). Number of CpGs used for prediction (left to right): 208,678; 100,598; 259,863; 45,822; 51,741; 20,309; 188,340; 8,861; 50,493; 9,150; 3,058; 7,453; 198,609; 212,907; 111,630 and 5,841.

Having established that our optimized workflow enables tumor class prediction within 1 h of sample receival, we next assessed the performance of MethyLYZR on our 75 samples. For 73 of the samples, we obtained high-confidence calls with a posterior probability greater than 0.6 from sequencing data obtained within the first 15 min and delivered diagnoses with an accuracy of 94.52% (Fig. 4a, Extended Data Fig. 6b and Supplementary Table 13). For those 22 biopsies with both rapid nanopore sequencing and EPIC methylation arrays available, we observed a high concordance in the diagnostic outcomes, underscoring the potential reliability and accuracy of our nanopore-based approach in a clinical setting (100% MZ CNS concordance; Extended Data Fig. 6c and Supplementary Tables 11 and 13).

To assess if the predictive power of our classifier would improve with prolonged sequencing time, we sampled all reads obtained along a detailed time grid from 5 min to 72 h for prediction. The most substantial increase in prediction accuracy was notable between 5 min and 15 min of sequencing. Beyond this interval, extended sequencing times resulted in only marginal accuracy improvement—94.52% versus 97.22%—highlighting the model’s efficiency in scenarios with limited information availability (Fig. 4b, Extended Data Fig. 6d and Supplementary Table 14). However, although current methods do not allow for copy number variation profiles from only 15 min of nanopore sequencing, extended analyses can be performed on the full 72 runs to obtain genome-wide copy number changes for a comprehensive neuropathologic assessment^31^ (Supplementary Figs. 2 and 3).

**Extended Data Fig. 7:**
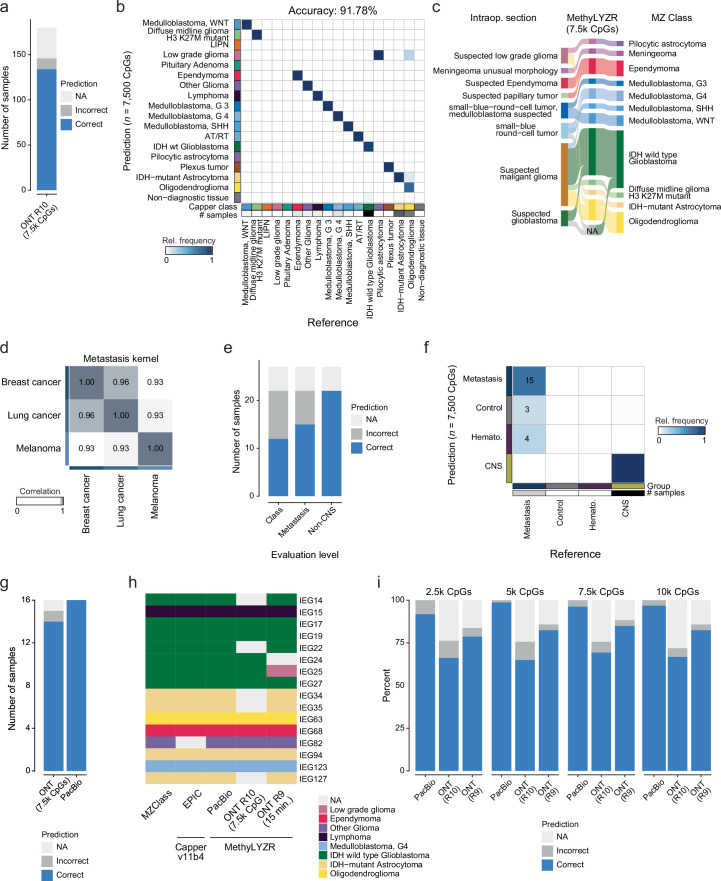
Evaluation of the barcoded Nanopore cohort and technology comparison. **a**) Barplot visualizing prediction outcome of the 180 brain cancer biopsies of 13 CNS cancer classes sequenced using barcoding on a PromethION R10 flow cell. For 80% of the 180 samples a classification was returned with an accuracy of 91.78%. **b**) Confusion matrix illustrating the prediction outcomes for all 180 barcoded Nanopore samples using 7.5k CpGs, resulting in an overall accuracy of 91.78% for MZ CNS classes. Misclassification errors are depicted by deviations from the bisecting line. **c**) For a subset of 26 samples with available intraoperative frozen section neuropathology (left) and matching Nanopore sequencing experiments (middle), MethyLYZR’s results showed full agreement with the broader rapid frozen section categories and more nuanced feedback aligned with current diagnostic groups (right). **d**) Correlation heatmap of the metastasis class kernels (breast cancer, lung cancer, and melanoma), showing correlations of >0.93. **e**) Barplot visualizing the 27 brain metastases mainly from lung, colon, and breast. 22 of these were predicted with a posterior above 0.6 (7.5k CpGs). 15 of the 22 were identified as metastasis and the 7 misclassifications were correctly identified as non-CNS cancer and assigned to control (n = 4) or hematopoietic (n = 3) groups. **f**) Confusion matrix illustrating the prediction outcomes for all 22 metastasis samples using 7.5k CpGs. 15 of the 22 were identified as metastasis and the 7 misclassifications were correctly identified as non-CNS cancer and assigned to control (n = 4) or hematopoietic (n = 3) groups. **g**) Barplot visualizing the prediction outcome of the 16 samples sequenced with Nanopore (first 15 minutes, rapid kit, R9) and PacBio HiFi (no posterior threshold due to high sequencing quality). **h**) Heatmap comparing the prediction accuracy of different sequencing technologies (EPIC with Capper classifier, PacBio HiFi, Nanopore R9 rapid, and R10 barcoded) for 16 samples from 8 different CNS classes. **i**) Visualization of prediction outcomes when using a limited number of CpGs (n = 2.5k, 5k, 7.5k, and 10k). All data were down-sampled 10 times to the CpG numbers on a per-read sampling basis.

Although our strategy requires library preparation and sequencing on a single-patient-per-one-flow-cell basis, we scaled our benchmarking to a higher throughput scenario. We sequenced 180 brain tumor biopsies covering 14 CNS tumor classes using rapid, multiplexed barcoded library preparation on PromethION R10 flow cells on P2 Solo and P24 systems (ONT), maintaining the same library preparation times per sample (180 nanopore libraries from 154 patients). MethyLYZR reported classifications for 147 samples with an overall MZ CNS class accuracy of 91.78% using CpGs obtained from read sampling resembling 15 min of sequencing (34 below threshold; Extended Data Fig. 7a,b and Supplementary Table 15). The model accurately identified prevalent classes (glioblastoma, astrocytoma and oligodendroglioma) as well as less common tumors, such as plexus tumor, atypical teratoid/rhabdoid tumor (AT/RT) and diffuse midline glioma with H3K27M mutation, demonstrating its effectiveness in a multiplexed, high-throughput setting.

To assess the clinical utility of MethyLYZR against conventional intraoperative frozen section neuropathology, we analyzed a subset of 26 brain tumor biopsies from our retrospective high-throughput cohort with available frozen section diagnoses. The results of MethyLYZR showed 100% categorial agreement with the broader rapid frozen section categories while providing nuanced feedback. This enhanced diagnostic precision, aligning better with integrated WHO diagnostic groups, could offer neurosurgeons more detailed insights than traditional intraoperative histopathological assessment (Extended Data Fig. 7c and Supplementary Table 15).

We extended our validation analysis to a cohort of 27 brain metastases from 20 patients, primarily from lung, breast and melanoma origins, with additional cases from colon cancer and endometrial cancer. Our training dataset for these metastases was limited, lacking data for colon and endometrial metastases and showing high kernel correlations (>0.93) among other metastasis types (Extended Data Fig. 7d). Given that the primary clinical concern is distinguishing metastases from primary brain tumors, we focused on classifying samples as either CNS tumors or non-CNS tumors (hematopoietic cancers, control group or metastases). MethyLYZR provided classifications for 81% of these samples, with most identified as metastases and none as CNS tumors (22 non-CNS: 15 metastases and seven control or hematopoietic cancer; Extended Data Fig. 7d–f and Supplementary Table 15).

We further evaluated the performance of MethyLYZR across different methylation profiling technologies by analyzing 16 samples using PacBio HiFi, Illumina EPIC arrays and both R9 rapid and R10 rapid barcoding nanopore protocols. This multi-platform approach allowed us to compare technology-specific error models and their impact on prediction accuracy. Due to the high accuracy of HiFi reads, we applied no posterior filtering to the PacBio data. In this limited sample set, MethyLYZR achieved a correct classification in 16 of 16 samples using the full PacBio dataset (no posterior filtering, similar to EPIC arrays), potentially surpassing both nanopore versions and the array-optimized DKFZ classifier (Extended Data Fig. 7g,h and Supplementary Table 16). This becomes specifically evident at lower numbers of CpGs, where tumors are characterized with higher accuracy and sensitivity by PacBio sequencing compared to nanopore sequencing (Extended Data Fig. 7i and Supplementary Table 17). However, the technology does not support real-time sequencing and is, therefore, infeasible for intraoperative classification.

**Extended Data Fig. 8:**
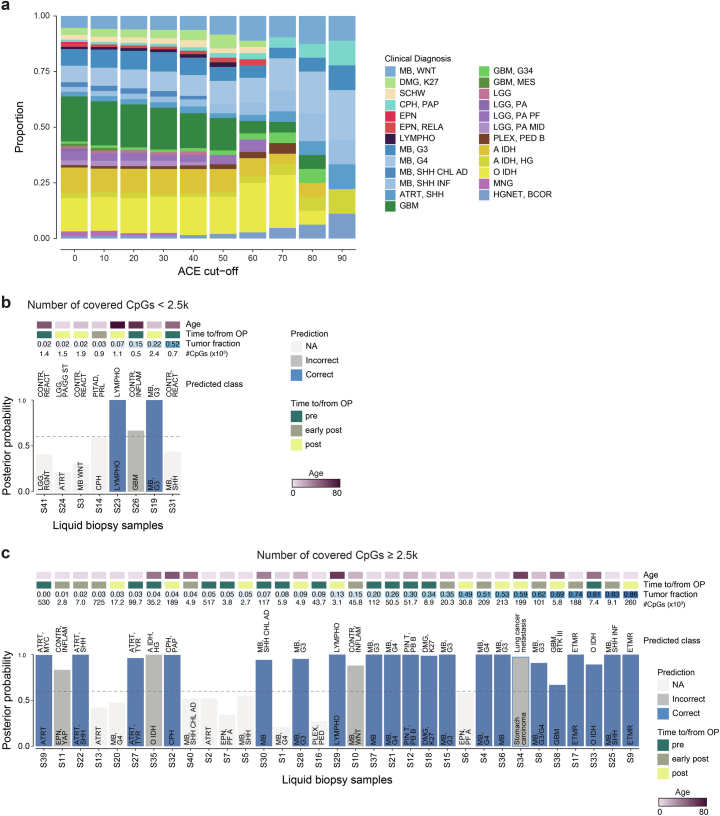
Tumor purity affects prediction accuracy. **a**) Proportions of tumor classes of tumor purity (ACE) stratified samples. **b**) Classification results of cell-free DNA from cerebrospinal fluid sequenced with Nanopore sequencing. 8 samples with limited quality showed less than 2.5k CpGs covered after filtering for reads within the expected length for cfDNA of 50-700nt. Annotations on top state the age of the donor, the time to/from operation, the estimated tumor cell content, and the number of CpGs. **c**) Classification results of cell-free DNA from cerebrospinal fluid sequenced with Nanopore sequencing. 33 samples with more than 2.5k CpGs covered after filtering for reads within the expected length for cfDNA of 50–700nt are shown. 17 had a tumor fraction above 0.1: one sample did not reach the posterior threshold; 15 of 16 were correctly predicted (93%), including a metastasis predicted as metastatic (instead of CNS). Annotations on top state the age of the donor, the time to/from operation, the estimated tumor cell content, and the number of CpGs.

Previous studies emphasized the critical role of tumor purity in robust CNS tumor classification^13–15^. Analyzing a nanopore dataset of 94 brain tumor samples matched with Illumina EPIC array data^13^, we noted a positive correlation between purity and MethyLYZR’s diagnostic accuracy. Enhanced correctness in classifications and fewer misclassifications were evident when purity exceeded 60%, with no errors above 70% (Fig. 4c and Supplementary Table 18). These findings underscore the importance of effective neurosurgical sampling and highlight the challenges of confidently diagnosing tumors, particularly for tumors with infiltrative growth or low cellularity (Extended Data Fig. 8a).

DNA methylation-based classification from cerebrospinal fluid (CSF) liquid biopsies offers a promising diagnostic tool, particularly for brainstem tumors, combining minimally invasive sampling with molecular insights^32^. We analyzed cell-free DNA (cfDNA) from 17 CSF samples^32^, selected for their typical histone-associated fragment size (50–700 bp in CSF^33^ and sample purity greater than 0.1). The complete analysis of the full cohort encompassing 41 samples with low CpG number and purity lower than 0.1 is presented in Extended Data Fig. 8b,c (Supplementary Table 19). This selection aimed to validate the ability of MethyLYZR to classify tumors based on authentic cfDNA, following its proven efficacy with cell-derived DNA. Although this experiment centered on cfDNA-specific analysis for liquid biopsy diagnostics, in clinical application MethyLYZR will be used to process methylation patterns from any DNA in clinical CSF samples. MethyLYZR accurately classified 15 of 16 samples that met the prediction threshold, including correctly identifying one metastasis as a non-CNS tumor, demonstrating its effectiveness in cfDNA-based tumor classification from CSF (Fig. 4d).

**Extended Data Fig. 9:**
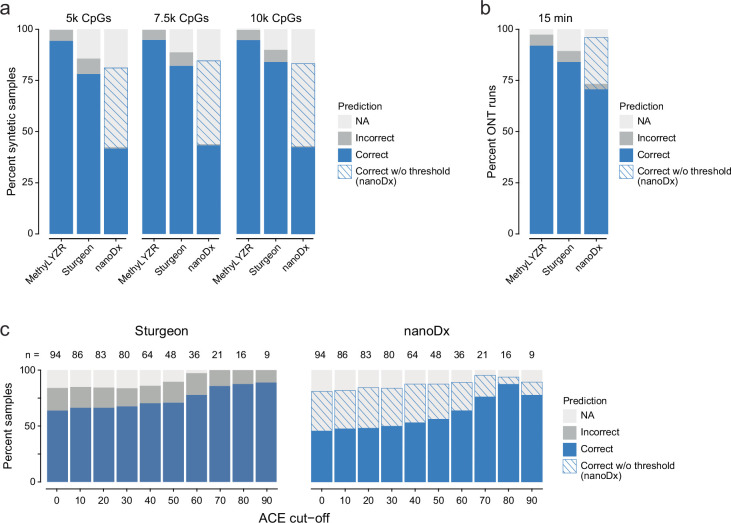
MethyLYZR predictions are more accurate than Sturgeon on shallow coverage, low CpG methylation information. **a**) Comparative analysis of MethyLYZR, Sturgeon, and nanoDx on the 100 ×2,801 simulated CNS samples based on 450k methylation array data. Predictions were conducted for 5k, 7.5k, and 10k CpGs. MethyLYZR predicts a class in >99% of samples with an average accuracy of 94.43%, 95.15%, and 95.25%, whereas Sturgeon reports for 89% of the samples a class with an accuracy of 92.61% (7.5k CpGs) and nanoDx in 42% with 98.69% (7.5k CpGs) accuracy (correct class, but below default reporting threshold indicated by blue diagonal lines). Of note, nanoDx has to re-train and thus requires more than 20 minutes per sample. **b**) Comparative analysis of MethyLYZR, Sturgeon and nanoDx on our 75 Nanopore runs. Predictions were conducted for CpGs that were sequenced within the first 15 minutes. MethyLYZR predicts a correct class in 94.52% out of 97.33% of samples, while Sturgeon only predicts 91.94% out of 82.67% of samples correctly and nanoDx predicts 98.69% out of 42% of samples correctly (correct class, but below default reporting threshold indicated by blue diagonal lines). **c**) Comparative analysis of Sturgeon and nanoDx matching the MethyLYZR analysis in Fig. 4c (Nanopore samples stratified by tumor purity, 73 of 94 predicted in full dataset, 82.19% accuracy). Of note, the predictions are based on only 7.5k CpGs to simulate 15 minutes of sequencing. While Sturgeon shows an accuracy of 75.95% (79/94 samples predicted), nanoDx shows an accuracy of 100% but low number of above-threshold classifications (43/94 samples predicted) and also 100% without the default threshold (correct class, but below default reporting threshold indicated by blue diagonal lines).

Finally, in a comparative analysis using our synthetic dataset, simulating 15 min of sequencing, MethyLYZR demonstrated superior performance with limited data compared to neural networks (Sturgeon) and random forest–based (nanoDx) predictions (5,000, 7,500 and 10,000 CpGs; Extended Data Fig. 9a and Supplementary Table 20). Corroborating these findings, the performance of MethyLYZR using actual nanopore data obtained within 15 min (7,500 CpGs in the case of tumor purity stratified data) surpassed the performance of both (Extended Data Fig. 9b,c and Supplementary Tables 13 and 21).

## Discussion

Our study suggests the potential applicability of MethyLYZR, a probabilistic naive Bayes classifier, for live molecular classification of nervous system malignancies using nanopore sequencing. Although further validation is needed, these initial results are promising and indicate the classifier’s capability in this context. The comprehensive evaluation across simulations, metastases, sarcomas and intraoperative clinical scenarios, along with its potential applicability in cfDNA-based diagnostics, underscores its versatility. Furthermore, the high concordance between our test cohorts’ predicted and actual tumor classes supports the model’s capability to deliver clinically relevant diagnoses. With the capability of MethyLYZR for live tumor prediction alongside nanopore sequencing, only DNA extraction time, library preparation and sequencer throughput are constraints for faster intraoperative results. Nevertheless, validation through multicentered clinical trials and prospective studies is still needed to ensure the model’s robustness across large and diverse sample cohorts and sequencing conditions, ultimately establishing its reliability and utility for clinical applications.

The results of this study also highlight a central use case for intraoperative neuropathology where all currently available intraoperative sequencing workflows fail, irrespective of the algorithm employed: identifying residual malignant cells at the tumor margins or distinguishing between active tumor and treatment effects upon suspected recurrence. Currently, high tumor cell purity is critical for obtaining reliable intraoperative sequencing classifications. As identifying epigenetic signatures of brain tumors with low tumor cell content from bulk sequencing data is essentially impossible regardless of the algorithm employed, we posit that this will be one of the next frontiers in live artificial intelligence (AI) algorithm development.

Notably, the accuracy of tumor class predictions using MethyLYZR, along with ad hoc random forest classifiers and neural networks^13–15^, reaches a similar plateau, indicating that these diverse methylation-based algorithms may have a similar upper limit in their ability to discern and interpret biological signals for cancer classification. However, although Sturgeon reports requiring approximately 1.25 h for the majority of tumor samples for extracting DNA from a biopsy to entity prediction^15^, MethyLYZR stays within the 1-h limit, aligning better with surgical timelines. In comparative analyses, MethyLYZR demonstrated superior accuracy compared to Sturgeon and nanoDx when evaluated under the constraints of this extremely short timeframe. This result is surprising, given that naive Bayes algorithms operate under the counterintuitive assumptions of feature independence (Methods) and simplicity, whereas neural networks excel at modeling complex interactions and dependencies between features. However, the sparsity and stochastic nature of data obtained intraoperatively may diminish the effectiveness of highly expressive AI systems. Additionally, ad hoc random forest classifiers face challenges in this context, as they require retraining for each sparse dataset, resulting in higher runtimes—more than 20 min for 7,500 CpGs with nanoDx compared to less than 1 min for MethyLYZR on the same system—which also aligns better with the concept of ‘model parsimony’ in clinical applications.

Model parsimony emphasizes the simplicity, effectiveness, tractability and transparency of diagnostic models. This principle advocates for the simplest yet effective explainable methods while acknowledging the well-documented limitations and consequences of traditional correlative analysis^34,35^. The approach does not negate the use of advanced machine learning technologies^36^ but, instead, suggests their application in scenarios where simpler models are insufficient. Looking ahead, integrating heterogeneous signals—such as DNA methylation, mutation signatures, copy number variations, breakpoint determinations and tumor purity with other modalities such as patient characteristics, magnetic resonance imaging (MRI) and Raman histology—into nonlinear neural network models will be the next challenge in molecular neuropathology. Leveraging simple probabilistic models such as MethyLYZR to inform and complement advanced AI systems promises substantial advancements in personalized diagnostics and treatment strategies aligned with transparency and explainability in medical care.

However, realizing the full potential of these technologies faces practical hurdles. Although the most comprehensive CNS tumor classifier to date was trained on more than 100,000 methylation arrays, a foundational dataset published in 2018 (ref. ^5^) used in this and other studies^13–15^ remains the only publicly available comprehensive resource for algorithm and clinical model development. Nevertheless, the most important limitation is the scarcity of publicly available, sequencing-based DNA methylation training and testing data. The lack of sufficient data not only constrains model development but also limits the validation and refinement of algorithms to integrate the vastly more informative data from all 32 million CpGs present in the human genome^37^.

Going forward, the advent of intraoperative diagnostics challenges the traditional healthcare system structure. An accurate diagnosis of CNS tumors achievable in under 1 h represents a paradigm shift, necessitating integrated workflows that span neurosurgery, neuropathology and neuro-oncology. The preclinical development of intraoperative tumor classification systems not only opens avenues for prospective clinical trials comparing different resection strategies^9^ and various other therapeutic modalities but also calls for a systemic change in personalized oncology to accommodate highly integrated, live diagnostic processes at the POC.

## Methods

### Patient material

#### Overview

Patient material and clinical data were collected from the Department of Neurosurgery of the University Medical Center Schleswig-Holstein (UKSH) in Kiel, Germany; from the Division of Neurosurgery of Vancouver General Hospital in Vancouver, Canada; and from the Department of Neurosurgery, University Medical Center Regensburg in Regensburg, Germany, after obtaining written informed consent of the donors for diagnostic procedures, comprising molecular testing including methylation profiling. The study was approved by and adhered to the Ethics Committee of the University of Kiel (D443/20); the University of British Columbia Research Ethics Committee (REB no. H08-02838); and the Ethics Committee of the University of Regensburg (20-1799-101) and is in accordance with the 1975 Declaration of Helsinki and its further amendments. All samples included were, moreover, routinely classified according to the current WHO classification^4^ by the Department of Neuropathology, University Medical Center Eppendorf, in Hamburg, Germany; the Department of Pathology & Laboratory Medicine, Faculty of Medicine, University of British Columbia, in Vancouver, Canada; or the Department of Neuropathology or University Medical Center Regensburg, in Regensburg, Germany, respectively. An overview of the clinical data is given in Supplementary Table 10.

#### Population characteristics

Patients with a radiologically suspected primary brain tumor or brain metastasis undergoing surgery at the UKSH, Campus Kiel, Department of Neurosurgery, without any age restrictions, were asked to participate in the study. As the classifier was trained on a publicly available microarray dataset, only post hoc analysis of the classifier results was performed without any further patient characteristics stratification. Biopsies of gliomas, including glioblastomas, astrocytomas and oligodendrogliomas, were sourced from archive collections at the Brain Tumor Center, University Medical Center Regensburg, and the Department of Pathology & Laboratory Medicine, Faculty of Medicine, University of British Columbia. Our research findings do not apply to only one sex or gender; no sex-based and gender-based analyses were performed; and sex and gender are not relevant to our research findings.

#### Recruitment

Patients scheduled for an open craniotomy due to a suspected primary brain tumor or brain metastasis were consecutively identified at the Department of Neurosurgery, UKSH, Campus Kiel. Patients were contacted and asked to participate. Patients initially agreeing to participate were assigned a study ID (IEGXXX). Depending on age and legal status, patients, their parents or their legal guardians signed an informed consent for using their tissue and clinical data in research (opt-in procedure). Patients who did not sign the informed consent documents, whose legal capacity to consent was unclear or whose ability to consent was questionable due to, for example, neurocognitive deficits, were excluded from the study. However, their IEGXXX designation was retained. Samples from Vancouver and Regensburg were assigned a consecutive IEGXXX number on the day they were sequenced. No patient compensation was provided.

#### Ethics oversight

The study protocol was approved by and adhered to the Clinical Ethics Committee of the Medical Faculty of Kiel University (D443/20). All included patients or their legal guardians/parents provided written informed consent for participation in the study. The results were not shared with treating physicians or caregivers and, therefore, not used to alter patient treatment or diagnosis. The study was approved by and adhered to the University of British Columbia Research Ethics Committee (REB no. H08-02838). The study was approved by and adhered to the Clinical Ethics Committee of the Medical Faculty of Regensburg University (20-1799-101).

#### Clinical Demonstrator video

Consent to publish the video, including the depiction of the individual researcher, was obtained in accordance with the General Data Protection Regulation (GDPR).

### DNA extraction of fresh brain biopsies

DNA extraction was performed using the QIAamp Fast DNA Tissue Kit (Qiagen) following the manufacturer’s protocol with minor modifications. In brief, 15 mg of brain tissue was weighed and transferred into a tissue disruption tube. Tissue lysis was done by adding 265 µl of digestion buffer mix, followed by sample homogenization at 45 Hz for 2 min using the TissueLyser LT (Qiagen). Protein and RNase digestion was subsequently carried out at 56 °C for 7 min and 1,000 rpm in a thermomixer (Eppendorf). The digested sample was supplemented with 265 µl of buffer MVL and homogenized by pipetting. The precipitated DNA mixture was loaded onto a QIAamp mini spin column and centrifuged at 20,000*g* for 1 min, followed by two wash steps with 500 µl of buffer AW1 and AW2 at 20,000*g* for 30 s. Residual ethanol was removed by centrifugation at 20,000*g* for 2 min. Elution of DNA was done using 50 µl of pre-heated (56 °C) nuclease-free water for 1 min, following a centrifugation step at 20,000*g* for 1 min. DNA quantification was carried out using a NanoDrop One (Thermo Fisher Scientific).

### Preparation of ONT sequencing libraries

The SQK-RAD004 sequencing kit (ONT) was used following the manufacturer’s recommendation with minor modifications to prepare low-throughput rapid libraries. In brief, 600–700 ng of QIAamp extracted DNA was transferred into a 0.2-ml PCR tube and adjusted to a total volume of 7.5 µl with nuclease-free water. The sample was supplemented with 2.5 µl of fragmentation mix (FRA) and placed into a pre-heated (30 °C) thermocycler (VWR, Doppio). Fragmentation was immediately performed at 30 °C for 1 min, following a heat inactivation step for 1 min at 80 °C. Attachment of the rapid sequencing adapter (RAP) was carried out for 10 min at room temperature (RT). Meanwhile, RT equilibrated MinION flow cells (ONT, FLO-MIN106D R9.4.1) were primed following the manufacturer’s protocol using RT equilibrated priming mix. Final libraries were supplemented with 34 µl of sequencing buffer (SQB), 25.5 µl of loading beads and 4.5 µl of nuclease-free water, and samples were immediately loaded onto R9.4.1 flow cells via the SpotON port.

Barcoded sequencing libraries for R10.4.1 flow cells were prepared using the rapid barcoding kit 24 V14 (ONT, SQK-RBK114.24) with minor modifications to the manufacturer’s protocol. For each library, up to 12 DNA samples (50–100 ng per sample) extracted with the QIAamp Fast DNA Tissue Kit (Qiagen) were used without the AMPure XP bead clean-up step. A total of 150 ng of barcoded library was loaded onto a primed R10.4.1 PromethION flow cell and sequenced for 48–72 h on either a P2 Solo or a P24 PromethION device. For intraoperative sequencing, a single freshly extracted tumor DNA sample was processed using the same kit, allowing for DNA extraction and library preparation within 35–40 min. The barcoded sample (100–150 ng) underwent sequencing adapter ligation and was loaded onto a primed R10.4.1 flow cell. Sequencing was continued for up to 72 h.

### Preparation of PacBio HiFi sequencing libraries

DNA samples were prepared for PacBio HiFi sequencing as follows: 3 µg of DNA per sample, extracted using the QIAamp Fast DNA Tissue Kit, was diluted in 100 µl of Tris-EDTA (TE) buffer and subjected to one cycle of Hydropore shearing on a Megaruptor 3 instrument (Diagenode) at speed 31. The sheared DNA was quantified using the Qubit dsDNA BR Kit and analyzed on a Fragment Analyzer 5200 (Agilent) with the HS Large Fragment 50 kb Kit to confirm proper fragmentation. Subsequently, each sample was processed using the SMRTbell prep kit 3.0 (PacBio, PN:102-182-700) according to the manufacturer’s protocol. The resulting barcoded libraries were divided into two pools and pooled equimolar. Three SMRT cells 25 M were sequenced on a PacBio Revio instrument with 24-h movies and SMRT Link software version 13.1.

### Illumina Infinium MethylationEPIC BeadChIP array generation

A subset of samples was analyzed using Illumina Infinium MethylationEPIC BeadChip (850,000) arrays (*n* = 22 Kiel, *n* = 2 Vancouver; Supplementary Table 2). In brief, the EZ DNA Methylation Kit (Zymo Research) was used according to the manufacturer’s instructions to perform bisulfite conversion of genomic DNA. Converted DNA was processed and subsequently hybridized to Infinium MethylationEPIC BeadChips (Illumina) following Illumina’s standard procedure. Scanning of Infinium MethylationEPIC BeadChips was performed using an Illumina NextSeq 550 system on default settings. Subsequent data analysis was performed using the minfi R package (version 1.32.0). For further analysis, loci showing detection *P* > 0.01 were excluded.

### Illumina Infinium MethylationEPIC BeadChIP array pre-processing

DNA methylation profiles based on methylation arrays for the model training were obtained from Capper et al.^5^ (CNS tumors GSE90496), Orozco et al.^29^ (metastases GSE108576) and Koelsche et al.^30^ (sarcomas GSE140686).

Our pre-processing and normalization workflow closely followed the procedures described by Capper et al.^5^. In brief, we first merged all samples from different sources into a unified dataset for further analysis. Then, the standardized Illumina normalization procedure was applied for both color channels to all samples independently by performing a background correction and a dye bias correction (the mean of control probe intensities scaled to 10,000).

Then, the following ambiguous or problematic probes were filtered: removal of probes targeting the X and Y chromosomes (*n* = 11,551); removal of probes containing a single-nucleotide polymorphism (dbSNP132 common) within five base pairs of and including the targeted CpG site (*n* = 7,998); removal of probes not mapping uniquely to the human reference genome (hg19 or hg38) allowing for one mismatch (*n* = 3,965); and removal of probes not included on the Illumina EPIC array (*n* = 32,260). In total, 428,201 probes targeting CpG sites were kept for further analysis. Batch effects caused by the material tissue type (formalin-fixed paraffin-embedded (FFPE) or frozen) were removed using a univariate linear model, separately for methylated and unmethylated signals.

### Nanopore data pre-processing

#### Validation cohort

The raw nanopore signals were processed into bases using the Dorado basecall server (version v7.0.9+1d91537ff from ONT) (https://github.com/nanoporetech/dorado/) implemented in the sequencing software MinKnow, using the high-accuracy basecalling model with 5mC modifications (dna_r9.4.1_450bps_modbases_5mc_cg_hac.cfg). The obtained reads were mapped to the human reference genome GRCH38.p13 (obtained from the UCSC Genome Browser) using minimap2 (ref. ^38^) (version v2.24-r1122) and saved into BAM files. Information on modified bases uses the MM and ML tags defined in the Sequence Alignment/Map Optional Fields Specification.

The extraction of the methylation values from the BAM files was done using a custom Python script (bam2feather.py). In short, the script first filters for primary alignments with a minimal mapping quality of 10, as reported by minimap2. Positions are further filtered on loci corresponding to a genomic position on the Illumina Infinium Human Methylation 450K BeadChip. The per-read and CpG methylation probability was calculated using the SAM MM and ML tags. The output is either directly streamed to MethyLYZR for prediction or written to disk as a feather file with the following information: epic_id (ID of the CpG position), methylation (probability of methylated position), scores_per_read (number of used CpG tags on the read), binary_methylation (methylation as a binary information), read_id (ID of the read), sfv azZSQtart_time (time in seconds form start of the sequencing run), run_id (ID of the sequencing run), QS (quality score, as reported by the basecaller) and read_length map_qs (mapping quality score, as reported by minimap2). For the pre-processing, Python v.3.8.10 was further used.

#### External cohort (purity analysis)

The available raw nanopore signals^13^ were processed into bases using the guppy basecall server (version 6.2.7+e9cbf95) from ONT using the high-accuracy basecalling model (version 2021-05-17_dna_r9.4.1_minion_384_d37a2ab9). The methylation information was extracted using megalodon from ONT (https://github.com/nanoporetech/megalodon; version 2.5.0), which uses the remora methylation calling algorithm. Mapping to the human reference genome GRCH38.p13 was done within megalodon with a version of minimap2. Results were then saved in an SQLite-Database (per_read_modified_base_calls.db). After extracting the per-read methylation information using the integrated megalodon command ‘megalodon_extras per_read_text modified_bases’ into a tab-separated text file, the methylation values were extracted using a modified version of bam2feather.py.

### Naive Bayes model for tumor classification

#### Objective

We use a naive Bayesian framework to allow tumor classification from sparse, shallow-coverage sequencing data. This model uniquely assumes conditional independence between features, which constitutes the here-required flexibility for the probabilistic classifier by focusing solely on the observed features.

The model calculates the probability of the tumor belonging to a certain class based on the observed methylation data. Mathematically, this involves integrating our prior knowledge about the probability of a tumor being of a certain type with the likelihood of the specific methylation pattern in the observed data (Fig. 1b, bottom).

Another key advantage of the naive Bayesian approach is its capacity to directly incorporate previous clinical knowledge into the predictions. Using population-wide tumor prevalence for the prior class distribution is a reasonable baseline assumption. Alternatively, if certain methylation patterns are known to be limited to particular clinical presentations, anatomical locations or age groups, this information could be factored into the predictions. This integration of experimental data with prior medical knowledge may further refine the model’s predictive accuracy in tumor classification.

#### Naive Bayes model

Let $X=({x}_{1},\,{x}_{2},\,\ldots ,{x}_{p})$, the tumor sample to be classified, be described by methylation rates ${x}_{i}$ of $p$ observed CpG loci. According to Bayes’ theorem, we update prior beliefs about the patient’s tumor type $P({C}_{j})$ in light of new evidence $X$ and model the probability of a tumor sample to be from class *C*_*j*_ a posteriori as

$$P({C}_{j}|X)=\frac{P(X|{C}_{j})P({C}_{j})}{P(X)}.$$

The denominator normalizes by the probability of observation $X$, which is defined as

$$P(X)=\mathop{\sum }\limits_{j=1}^{m}P(X|{C}_{j})P({C}_{j}),$$

dependent only on $X$—hence, a constant scaling factor independent of tumor class ${C}_{j}$, ensuring that the sum of conditional probabilities over all $m$ classes equals 1. Here, we implement the calculation of the denominator using the log-sum-exp trick, where

$$\begin{array}{l}\log P(X)=\,\log \mathop{\sum }\limits_{j=1}^{m}\exp ({l}_{j})={l}_{\mathrm{max}}+\,\log \mathop{\sum }\limits_{j=1}^{m}\exp ({l}_{j}-{l}_{\mathrm{max}}),\\\quad\,{\rm{with}}\,{l}_{j}=\,\log P(X|{C}_{j})P({C}_{j})\;{\rm{and}}\,{l}_{\mathrm{max}}=\mathop{\max }\limits_{j\in \{1,\,\ldots ,\,m\}}{l}_{j}\end{array}$$

is applied to avoid numerical underflow.

Notably, the prior $P({C}_{j})$ provides the basis for the direct integration of previous clinical information into the prediction.

In the naive Bayes classifier, the likelihood $P({X|}{C}_{j})$ is calculated under the assumption of conditional independence between the features in $X$:

$$P(X|{C}_{j})=P({x}_{1},\ldots ,{x}_{p}|{C}_{j})=\mathop{\prod }\limits_{i=1}^{p}P({x}_{i}|{C}_{j}).$$

This makes the model insensitive to missing data, as the likelihood can be calculated based solely on observed attributes. In our case, this central property allows for classification from shallow nanopore sequencing, where only a subset of features is available.

As we are dealing with binary feature values ${x}_{i}\in \left\{\mathrm{0,1}\right\}$ from the observation of methylation events, the feature-specific likelihoods $P\left({x}_{i}|{C}_{j}\right)$ are derived using a Bernoulli distribution

$$P\left({x}_{i}|{C}_{j}\right)={\mu }_{i,j}^{{x}_{i}}{(1-{\mu }_{i,j})}^{(1-{x}_{i})},$$

where ${\mu }_{i,j}=P\left({x}_{i}=1\right|{C}_{j})$ are corresponding pre-trained, class-specific methylation probabilities.

Such that the posterior probability of a tumor sample, observed by $X=({x}_{1},\,{x}_{2},\,\ldots ,{x}_{p})$, belonging to class ${C}_{j}$, is calculated by

$$P\left({C}_{j}{|X}\right)=\frac{\mathop{\prod }\nolimits_{i=1}^{p}P\left({{x}_{i}{|C}}_{j}\right)P({C}_{j})}{P\left(X\right)}=\frac{\mathop{\prod }\nolimits_{i=1}^{p}{\mu }_{i,j}^{{x}_{i}}{(1-{\mu }_{i,j})}^{(1-{x}_{i})}P\left({C}_{j}\right)}{P\left(X\right)}.$$

To avoid numerical underflow when multiplying a substantial number of probabilities, we use the logarithmic form for the calculation of posterior probabilities:

$$\log P\left({C}_{j}{|X}\right)=\log P({C}_{j})+\mathop{\sum }\limits_{i=1}^{p}\log P\left({{x}_{i}{|C}}_{j}\right){\rm{\mbox{--}}}\log P\left(X\right),$$

where

$$\log P\left({{x}_{i}{|C}}_{j}\right)={x}_{i}\cdot \log \left({\mu}_{i,j}\right)+\left({1-x}_{i}\right)\cdot \log \left({1-\mu }_{i,j}\right).$$

#### Decision rule

Finally, our classifier infers the class label $\hat{{\rm{C}}}$ of a tumor with feature vector $X$ by assignment to the class with highest posterior probability:

$$\hat{{\rm{C}}}({\rm{X}})=\mathop{{\rm{argmax}}}\limits_{j\, \in \, \{1,\, 2,\,\ldots ,\,m\}}P({C}_{j}|X),$$

thereby minimizing the expected number of classification errors.

To ensure high precision of the classifier—at the cost of potentially lower recall—we return only high-certainty predictions, by introducing a posterior probability threshold $\tau \in \left\{\mathrm{0,1}\right\}$. A prediction with a posterior probability $P\left({C}_{j}{|X}\right)$ is deemed to be of low certainty if $P\left({C}_{j}{|X}\right) < \tau$ and of high certainty if $P\left({C}_{j}{|X}\right)\ge \tau$.

#### Training

Training of the naive Bayes model comprises derivation of class prior probabilities and conditional probabilities for each feature across all classes. The prior probabilities $P\left({C}_{j}\right)$ are estimated based on the empirical distribution of tumor classes within the respective training dataset. Due to the continuous nature of methylation rates in the training data—diverging from the assumption of binary variables in the Bernoulli naive Bayes—we infer the conditional probabilities $P\left({x}_{i}=1\right|{C}_{j})$ directly from class centroids, calculated as the mean methylation rates, ${\mu }_{i,j}$, for each CpG across samples within each tumor class (Fig. 1b, top).

#### Weighting

The two strong assumptions made by the naive Bayes model—conditional independence between features and equal contribution of features to the outcome—notably simplify computation but may not always hold true for real-world data, such as CpG methylation in cancer tissue. Individual cancer epigenomes display a highly correlated structure (Extended Data Fig. 1a), and not all CpGs carry the same information weight for each cancer class^5^, violating these assumptions. Nonetheless, information-theoretic work has shown that naive Bayes classifiers are also remarkably accurate if the features being modeled are functionally highly dependent^39,40^. Various feature-weighting approaches have been proposed to enhance performance of the naive Bayes, mainly by relaxing the naive independence assumption^23–25^. Here, we alleviate the caveat of attribute independence via feature weights, taking into account inter-class and inter-feature relationships.

Class-specific feature weights ${w}_{i,\,j}$ are integrated into the naive Bayes model when calculating the likelihoods $P\left({{x}_{i}{|C}}_{j}\right)$ as follows:

$${P\left({{x}_{i}{|C}}_{j}\right)}^{{w}_{i,j}}={\left[{\mu }_{i,j}^{{x}_{i}}{(1-{\mu }_{i,j})}^{(1-{x}_{i})}\right]}^{{w}_{i,j}}.$$

It follows for the calculation in log space:

$$\begin{array}{c}\log P{({x}_{i}|{C}_{j})}^{{w}_{i,\,j}}=\,\log {\left[{\mu }_{i,\,j}^{{x}_{i}}{(1-{\mu }_{i,\,j})}^{(1-{x}_{i})}\right]}^{{w}_{i,\,j}}\\ ={w}_{i,\,j}({x}_{i}\cdot \,\log ({\mu }_{i,\,j}))+{w}_{i,\,j}((1-{x}_{i})\cdot \,\log (1-{\mu }_{i,\,j})).\end{array}$$

When applying class-specific attribute weighting, the likelihoods $P({{X|C}}_{j})P({C}_{j})$ in the denominator of Bayes’ rule need to be adjusted per class to account for class-specific differences in the sum of feature weights.

#### ReliefF-based algorithm for feature weighting

To enhance the model’s ability to discern classes often confused due to highly similar or correlated profiles, we employed an adapted version of the ReliefF algorithm^21,22^ for quantification of tumor-class-specific informativeness of each feature (Extended Data Fig. 1b).

First, pairwise distances between all samples and class centroids are calculated. To derive weight ${\omega }_{i,j}$ for a specific feature with index *i* and class ${C}_{j}$, the algorithm performs the following steps for each sample in class ${C}_{j}$:

1. Calculate the mean distance to the remaining samples in class ${C}_{j}$ (referred to as ‘hits’, denoted as $h$).
2. Identify the *k*-nearest centroids, which are not class ${C}_{j}$ (these are the ‘misses’, denoted as $m$), and calculate the mean miss distance.
3. Subtract the mean hit distance from the mean miss distance.

Then, feature weight ${\omega }_{i,\,j}$ is derived by summing the resulting values from all samples in class ${C}_{j}$, such that

$$\omega_{i,\,j} = \sum\limits_{x \in C_j} \left\{ \frac{\sum\limits_{m \in KNN\,(x) \atop l(m) \neq C_j} |x_i - m_i|}{k} - \frac{\sum\limits_{h \neq x \atop h \in C_j} |x_i - h_i|}{|C_j| - 1} \right\},$$

where ${\mathrm{KNN}}\left(x\right)$: *k*-nearest centroids of $x$ and ${\rm{l}}:\,X\to C$ maps centroids to classes, such that for informative features ${\omega }_{i,\,j} > 0$ and for uninformative features ${\omega }_{i,\,j} < 0$. Here, we use $k=5$ for the number of considered nearest centroids.

According to Foo et al.^41^, the derived feature weights $${\omega }_{i,j}\,$$$${\mathbb{\in }}\,{\mathbb{R}}$$ are transformed by

$${w}_{i,\,j}={e}^{-{\omega }_{i,\,j}}$$

before being used in the naive Bayes model.

### Complexity analysis of MethyLYZR training

**1 Aim**

Evaluating how training of our ReliefF-weighted naive Bayes classifier scales with

- number of features
- number of classes
- sample size

For this, evaluating the time complexity of

- calculation of centroids
- calculation of feature weights

with respect to the above parameters.

**Variables**

- $p$: number of features—that is, CpGs
- $m$: number of classes
- ${n}_{j}$: number of samples in class ${C}_{j}\left(j\in \{1,\,\ldots ,\,m\}\right)$
  - → such that $N=\sum _{j}{n}_{j}$ is the number of all samples

**2 Calculation of centroids**

For each class $C_j$

- → there is a total of $m$ classes
  - for each sample in that class
    → there is a total of ${n}_{j}$ samples in class *C*_*j*_
    • iterate over all $p$ features $\in {\mathcal{O}}(p)$
  - $$\in \mathcal{O}({n}_{j}\cdot p)$$
- such that this adds up to a time complexity over all $m$ classes of:
  $${\mathcal{O}}(p\cdot ({n}_{1}+{n}_{2}+\ldots +{n}_{m}))$$
- Because $N={\sum }_{j}{n}_{j}$, the time complexity for calculating the class centroids can be specified as:
  $$\mathcal{O}(p\cdot N)$$

**3 Calculation of feature weights**

**3.1 Pre-calculating pairwise distances**

For each class $C_j$

- → there is a total of $m$ classes
  - comparing the mean profile with each sample
    → there is a total of $N$ samples across all classes
    • for each comparison iterating over all $p$ features to calculate distance $\in \mathcal{O}(p)$
  - $$\in \mathcal{O}(N\cdot p)$$
- such that this adds up to a time complexity over all $m$ classes of:
  $$\mathcal{O}(m\cdot N\cdot p)$$

**3.2 ReliefF-like algorithm**

Here, we are iterating over the classes, but, practically, we are doing the same for each sample, so, for complexity, considering cost per sample.

**3.2.1 Identifying** ${\boldsymbol{k}}$ **nearest misses**

For each sample

- → there is a total of $N$ samples
  - sorting the pre-calculated distances to the $m$ average class profiles to identify $k$ nearest misses $\in{\mathcal{O}}({m}\,{\log}\,{m})$

such that this adds up to a time complexity over all samples of:

$$\mathcal{O}(N\cdot m\,\log m)$$

**3.2.2 Calculating mean miss distance**

For each sample

- → there is a total of $N$ samples
  - calculating the absolute difference between the sample and its $k$ nearest misses
    • for each instance iterating over all $p$ features to calculate distance $\in \mathcal{O}(p)$
  - $$\in \mathcal{O}(k\cdot p)$$

such that this adds up to a time complexity of ${\mathcal{O}}{\left(N{\cdot} k{\cdot} p\right)}$ over all samples, which can be simplified assuming a constant number $k=5$ when considering the $k$ classes as misses to:

$${\mathcal{O}}{\left(N{\cdot} p\right)}$$

**3.2.3 Calculating mean hit distance**

For each sample in class ${C}_{j}$

- → there is a total of ${n}_{j}$ samples
  - calculating the absolute difference between the sample and the remaining ${n}_{j}-1$ samples of the same class
    • for each instance iterating over all $p$ features to calculate distance $\in \mathcal{O}(p)$
  - $$\in \mathcal{O}(({n}_{j}-1)\cdot p)\in \mathcal{O}({n}_{j}\cdot p)$$

such that this adds up to a time complexity of $\mathcal{O}({n}_{j}^{2}\cdot p)$ over all ${n}_{j}$ samples in one class ${C}_{j}$.

Summing this over all $m$ classes adds up to:

$$\mathcal{O}(p\cdot ({n}_{1}^{2}+{n}_{2}^{2}+\ldots +{n}_{m}^{2}))$$

**3.3 Summarized time complexity of ReliefF-like feature weight calculation**

Combining all the above steps, including pre-calculating the pairwise distances and the ReliefF-like algorithm, the time complexity is

$$\mathcal{O}(m\cdot N\cdot p)+\mathcal{O}(N\cdot m\,\log m)+\mathcal{O}(N\cdot p)+\mathcal{O}(p\cdot ({n}_{1}^{2}+{n}_{2}^{2}+\ldots +{n}_{m}^{2}))$$

and because $\mathcal{O}(N\cdot p)$ is bounded above by $\mathcal{O}(m\cdot N\cdot p)$, it follows that this is in

$$\mathcal{O}(m\cdot N\cdot p)+\mathcal{O}(N\cdot m\,\log m)+\mathcal{O}(p\cdot ({n}_{1}^{2}+{n}_{2}^{2}+\ldots +{n}_{m}^{2})).$$

Furthermore, in the case of a high-dimensional data space, we can assume that the number of features $p$ is considerably larger than the number of classes $m\,(p\gg m)$. Following this, when considering ${\mathcal{O}}({m}{\cdot}{N}{\cdot}{p})$ and ${\mathcal{O}}({N}{\cdot}{m}\,{\log}{m})$, the ${\log{m}}$ part grows much slower than $p$, such that $\mathcal{O}(m\cdot N\cdot p)$ can be assumed to dominate the term, and the upper-bound complexity can be approximated as

$$\mathcal{O}(m\cdot N\cdot p)+\mathcal{O}(p\cdot ({n}_{1}^{2}+{n}_{2}^{2}+\ldots +{n}_{m}^{2})).$$

**4 Total time complexity of MethyLYZR training**

Combining the above-derived complexity for

1. calculation of centroids
  → $$\mathcal{O}(p\cdot N)$$
2. calculation of feature weights
  → $$\mathcal{O}(m\cdot N\cdot p)+\mathcal{O}(p\cdot ({n}_{1}^{2}+{n}_{2}^{2}+\ldots +{n}_{m}^{2}))$$

it can be seen that, with $\mathcal{O}(N\cdot p)\in \mathcal{O}(m\cdot N\cdot p)$, the upper-bound complexity of MethyLYZR training can be described by

$$\mathcal{O}(m\cdot N\cdot p)+\mathcal{O}(p\cdot ({n}_{1}^{2}+{n}_{2}^{2}+\ldots +{n}_{m}^{2}))$$

**5 Interpretation**

- As assumed, the number of classes $m$ alone does not dominate the complexity. It appears only linearly in the first term, making it relatively efficient as $m$ becomes larger.
- However, the complexity is more dependent on the actual number of samples $N$ and the number of features $p$ that the model is trained on.
- Notably, all terms scale only linearly with the number of features $p$.
- The quadratic sum in the last term shows that the distribution of the samples across the classes is also essential. If one or more classes exhibit a substantially higher number of samples, then the last step in the ReliefF-based method can get more computationally heavy. In the most extreme dysbalanced case, where some class ${C}_{j}$ holds almost all samples $\left({N}_{j}\approx N\right)$, the computational complexity can be described by $\mathcal{O}(p\cdot ({n}_{1}^{2}+{n}_{2}^{2}+\ldots +{n}_{m}^{2}))\in \mathcal{O}(p\cdot {N}^{2})$. Taken together, the upper bound complexity is quadratic in the number of samples.

#### Feature independence

The naive Bayes classifier relies on the feature independence assumption, which is violated both for methylation rates obtained from an array and for methylation values obtained from Oxford Nanopore Technologies. Supplementary Fig. 1 shows the Pearson correlation and respective weights per class among those CpGs that were sequenced by the nanopore runs IEG4, 5 and 6 within the first 15 min as well as a summary across correlations and feature weights from all R9 runs. The strong correlation between the single CpGs leads to a highly correlated structure of the centroids, as shown in Extended Data Fig. 1a.

Nonetheless, several works in information theory have shown that naive Bayes classifiers are also remarkably accurate if the features being modeled are functionally highly dependent^39,40^. The literature has proposed various strategies that relax the naive independence assumption of the Bayes classifier^24,25,42^.

To address the potential dependence of features (for example, CpGs), we incorporated a feature-weighting scheme in MethyLYZR using the ReliefF algorithm^21,22^ that considers both inter-class and inter-feature relationships. In short, the ReliefF weighting algorithm assesses tumor-class-specific informativeness of each feature by the comparison of inter-class and inter-feature distances (see the Methods ‘ReliefF-based algorithm for feature weighting’ subsection). The calculation of these weights needs to be done only once during model training, and its complexity can be approximated by $\mathcal{O}(m\cdot N\cdot p)+\mathcal{O}(p({n}_{1}^{2}+{n}_{2}^{2}+\ldots +{n}_{m}^{2}))$, with number of classes $m$, number of samples in class $i$ ${n}_{i}$, number of features $p$ and total number of samples $N$ (see complexity analysis section above).

### Prediction

#### Read filtering and read weighting

In case of highly correlated features, the model’s predictions can be skewed, as they might overly rely on the correlated features at the expense of other potentially informative, independent features (Supplementary Fig. 1a,b and Methods above). To ensure that reads covering a high number of feature CpGs do not disproportionately influence the prediction, we implemented a read-filtering and read-weighting approach.

Initially, CpG methylation calls stemming from reads that cover more than 10 of the pre-defined feature CpGs are excluded from the prediction. Subsequently, we assign a read weight ${r}_{i}=\frac{1}{{\mathrm{no.}}\,{\mathrm{of}}\,{\mathrm{features}}\,{\mathrm{on}}\,{\mathrm{same}}\,{\mathrm{read}}}$ to each measured feature in a sample, which downweights the influence of reads with a high density of features that are more likely to be correlated, thus reducing their impact in the model. Then, resulting class log-likelihoods are scaled by the factor $\frac{300}{\sum {r}_{i}}$, where 300 is a base count of the number of reads that we standardize to.

#### Methylation calling

Features for prediction are filtered and converted to binary methylation calls by the methylation probability (see above: bam2feather.py). CpGs with methylation probabilities of 0.2 or below are considered unmethylated (0); features with methylation probabilities of 0.8 or above are considered methylated (1); and all calls with intermediate probabilities are discarded (0.3–0.7 for R10).

For each feature of a sample, noise terms ${\eta}_{i}$ that quantify the uncertainty of methylation calls with probability ${m}_{i}$ are calculated using ${\eta}_{i}=0.5-\left|{m}_{i}-0.5\right|$; values below 0.05 are set to this minimum threshold. For prediction of a specific sample, its noise terms ${\eta}_{i}$ are integrated into the model’s centroids by setting ${\mu {\prime} }_{i,j}$ = ${\mu }_{i,j}$ – ${\mu }_{i,j}\cdot$ 2${\eta}_{i}$ + ${\eta}_{i}$.

#### Threshold analysis

The model’s performance was systematically evaluated at posterior probability decision thresholds between 0 and 1 with increments of 0.1. The optimal cutoff $\tau =0.6$ was chosen as the point that provided the optimal balance between sensitivity and specificity (precision and recall), ensuring high precision of the returned predictions (Extended Data Fig. 6b).

### Model evaluation

#### Evaluation metrics

The performance of the models was assessed by considering the accuracy ($\frac{{\mathrm{TP}}+{\mathrm{TN}}}{{\mathrm{TP}}+{\mathrm{TN}}+{\mathrm{FP}}+{\mathrm{FN}}}$), where ${\mathrm{TP}}$: true positives, ${\mathrm{TN}}$: true negatives, ${\mathrm{FP}}$: false positives and ${\mathrm{FN}}$: false negatives) across all samples and per class, as well as the F1 score ($\frac{2\cdot {\mathrm{precision}}\cdot {\mathrm{recall}}}{{\mathrm{precision}}+{\mathrm{recall}}}$) per class.

#### Models

The CNS MethyLYZR model was trained on 2,801 samples from 91 CNS tumor and control classes^5^, covering the above-described 428,201 CpGs as features.

To validate the homogeneity of the CNS tumor classes defined by Capper et al., we used the gap statistic, a formalized procedure of the heuristic elbow method for cluster number estimation^43^. For each pre-defined tumor class, we employed the gap statistic method with *k*-means clustering to identify potential intra-class clusters. As the maximum gap statistic value indicates the optimal number of clusters *k*, all homogenous classes are expected to show a maximum at *k* = 1 (Supplementary Fig. 4).

The extended CNS and metastasis model was trained on the 2,801 CNS samples plus 85 samples from three metastatic classes. Likewise, the extended CNS, metastasis and sarcoma model was trained on 2,801 CNS samples plus 85 metastasis samples plus 1,077 samples from 64 sarcoma classes.

#### Tiered class evaluation

In the evaluation of our machine learning model, we employed a multi-tiered accuracy assessment approach. Initially, accuracies were calculated for each CNS class predicted by the model, providing a granular view of its performance. Subsequently, we introduced an additional layer of evaluation by aggregating the 91 CNS classes into 44 broader MZ CNS classes of high clinical relevance, allowing an assessment of the model’s impact on clinical considerations. Additionally, we also evaluate the model’s performance on the level of eight broad MCFs, summarizing histologically and biologically closely related tumors as described by Capper et al.^5^.

#### Synthetic data

To comprehensively evaluate the model’s performance, synthetic datasets mirroring methylation patterns obtained by shallow nanopore sequencing were created by sampling from an underlying distribution of methylation events. Specifically, for each sample in the reference datasets, 100 binary replicates covering all CpGs were sampled using a Bernoulli distribution, where methylation event probabilities were derived from methylation rates as observed by corresponding 450k methylation arrays. Then, to simulate various coverage levels, we randomly selected subsets of 1,000, 2,500, 5,000, 7,500, 10,000, 15,000 or 20,000 CpGs per synthetic nanopore profile.

In the prediction of synthetic profiles, read weights were set to a constant value, thus having no impact, and noise was uniformly set to 0.05 for all features. Furthermore, no posterior thresholds were applied.

#### Time-course analysis (ONT validation cohort)

To evaluate the accuracy of the predictions in a real-time scenario, we post hoc filtered reads by their timestamps to obtain CpG methylation calls obtained after 5 min, 10 min, 15 min, 30 min, 45 min, 1 h, 3 h, 5 h, 12 h, 24 h, 48 h and 72 h of sequencing.

#### Benchmarking

For evaluation of our model’s training time and resource utilization, we used the CNS tumor methylation array dataset, encompassing a total of 2,801 samples across 91 tumor classes, each with 428,201 features. To measure training time and memory usage of the algorithm, we employed Python’s time and memory_profiler packages. Benchmarking was performed in two computing environments: a high-performance server (Dell PowerEdge R7525, 3 GHz AMD 64-Core Processor, 256 CPUs, 1,031.3 GB DDR4 RAM, Linux distribution) and a 2017 Apple iMac (3 GHz 10-Core Intel Xeon W, 64 GB 2,666 MHz DDR4 RAM, 1 TB APFS SSD, Radeon Pro Vega 56 GPU with 8 GB VRAM, macOS v.13.2.1).

Additionally, for each of the 75 nanopore sequencing runs, we evaluated the model’s prediction latency at various sequencing durations, by extracting data from 5 min up to 72 h (5 min, 10 min, 15 min, 30 min, 45 min, 1 h, 3 h, 5 h, 12 h, 24 h, 48 h and 72 h). These prediction benchmarks were conducted on the Apple iMac (above).

#### Post hoc simulation of low-coverage sequencing

To simulate short, low-coverage sequencing from full sequencing runs (R10 barcoded, PacBio HiFi, external purity cohort), we employed a read sampling method to preserve the sequencing read context of the CpG methylation calls. This was achieved by randomly sampling reads from the data until the number of covered CpG sites reached the target number—for example, 7,500 to imitate 15 min of nanopore sequencing. The measured methylation states for each CpG site within the selected reads were used as input for classification. To assess the impact of various coverage levels, datasets with 2,500, 5,000 and 7,500 CpGs were generated and subsequently evaluated, with 10 replicates produced for each sample and CpG count.

#### Tumor purity analysis

To evaluate the model’s sensitivity to tissue sample impurity, we integrated a dataset of 95 ONT-sequenced brain tumor samples with estimated tumor purities via absolute copy number estimation (ACE) as described by Djirackor et al.^13^. We then assessed the predictive performance at purity thresholds ranging from 0% to 90%, in 10% increments. For the analysis, we mimicked the intraoperative case and considered only 7,500 CpGs obtained by the above-described read sampling.

#### Liquid biopsies

cfDNA shows a typical fragment length distribution (120–220 bp), peaking at 167 bp, corresponding to the length of DNA wrapped around one, two or three histones. In CSF samples from patients with brain tumors, cfDNA fragments were observed to be slightly shorter, enriching at 145 bp^33^. However, liquid biopsies from CSF are likely contaminated by intact cells taken up during the needle biopsy. To mitigate the potential interference from non-cfDNA contaminants, such as cellular genomic DNA, on our benchmarking, we implemented a rigorous filtering strategy based on fragment size distribution analysis.

In 2024, Afflerbach et al.^32^ published a set of nanopore brain cancer samples for which cfDNA was derived from CSF. From their initially obtained samples, 85% (129/178) contained at least 5 ng of DNA, which was their threshold for preparing a nanopore sequencing run. In 39% (50/129) of the cases, cfDNA was successfully sequenced, and, of those, 41 were available to us. Clinical specifics of these data are provided in Supplementary Table 19. In brief, 16 samples were collected pre-operation, 11 early post-operation and 14 later post-operation (>14 d).

1. In the initial step, we excluded reads with a length profile outside of what is considered cfDNA and kept only reads with a length of 50–700 nucleotides.
2. Subsequently, we split the cohort by the number of covered CpGs, where a very low number (*n* = 8 samples) indicates that problems with the library might have occurred or that a high fraction of bacterial contamination was present.
3. We then ordered the samples by estimated tumor fraction and used only those with a fraction of at least 0.1 (*n* = 17) for validation.

#### Comparative analysis with Sturgeon

Sturgeon^15^ was applied to the same synthetic dataset described above, encompassing 280,100 samples from CNS classes. For this, BED files with binary methylation information were generated with the same CpG probes used for MethyLYZR input—covering 5,000, 7,500 and 10,000 CpGs randomly drawn to simulate sparse nanopore sequencing. Additionally, Sturgeon was used for classification of data from 15 min of sequencing from the 75 R9 nanopore CNS tumor samples. For this, BED files with binary methylation information were generated with the same CpG probes used for MethyLYZR input—filtered for methylation probabilities below 0.2 or above 0.8. Similarly, Sturgeon was used for classification of the external cohort for purity analysis, where BED files were generated with binary methylation information from 7,500 CpG probes obtained by read sampling. The Sturgeon classifier (https://github.com/marcpaga/sturgeon, commit hash: b9f1cf565ce17eb43957b9c1acb5ea15a480e23e) was executed using the provided general model (https://www.dropbox.com/s/yzca4exl40x9ukw/general.zip?dl=0). Results with scores lower than 0.8 were considered inconclusive. Comparative evaluation of predictive accuracy across MethyLYZR and Sturgeon was done based on 87 merged classes predicted by Sturgeon, where ‘MB SHH – CHL AD INF’ summarizes ‘MB, SHH CHL AD’ and ‘MB, SHH INF’; ‘SUBEPN – ALL’ summarizes ‘SUBEPN – PF’, ‘SUBEPN – SPINE’ and ‘SUBEPN – ST’; and ‘LGG PA’ summarizes ‘LGG, PA MID’ and ‘LGG, PA.’

#### Comparative analysis with nanoDx

Analogously to Sturgeon, nanoDx^14^ was applied to the synthetic datasets, the 15-min R9 nanopore CNS samples and the tumor purity cohort. CSV files with EPIC probe IDs and corresponding methylation levels were prepared as the input for nanoDx. Modules from the nanoDx classifier version 0.6.2 (https://gitlab.com/pesk/nanoDx, commit hash: b31d30fd690bb10d18c45abc3d8934ff0b0b6062), including ‘readCpGs.R’, ‘transform_Rdata.R’, ‘feature_selection_tfidf.py’ and ‘pyRF5xCVrecal.py’, were executed using Snakemake^44^ (version 8.14.0) with parameters ‘—cores 1’. For ad hoc model training, the Heidelberg brain tumor classifier version 11b4 reference set^5^, which was downloaded from https://gitlab.com/pesk/nanoDx, was used as the training dataset. Results with scores lower than 0.15 were considered inconclusive.

### Statistical analysis and reproducibility

All statistical analyses were performed in R version 4.2.2 (ggpubr version 0.5.0, RColorBrewer version 1.1-3, arrow version 10.0.1, rtracklayer version 1.58.0, GenomicRanges version 1.50.2, ggsci version 2.9, circlize version 0.4.15 and tidyverse version 1.3.2) and Python version 3.11.7 (arrow version 1.3.0, contourpy version 1.2.0, cycler version 0.12.1, Cython version 0.29.36, fonttools version 4.47.2, importlib-resources version 6.1.0, kiwisolver version 1.4.5, mappy version 2.26, matplotlib version 3.8.2, memory-profiler version 0.61.0, natsort version 8.4.0, numpy version 1.26.3, packaging version 23.2, pandas version 2.2.0, pillow version 10.2.0, pyarrow version 15.0.0, pyparsing version 3.1.1, pysam version 0.22.0 with SAMtools version 1.16.1 and htslib version 1.16, python-dateutil version 2.8.2, pytz version 2024.1, scipy version 1.12.0, setuptools version 44.0.0, six version 1.16.0, tabulate version 0.9.0, types-python-dateutil version 2.8.19.20240106, tzdata version 2023.4, watchdog version 3.0.0 and zipp version 3.17.0). Confusion matrices and heatmaps were plotted using the ComplexHeatmap package (version 2.14.0). All other plots were generated using the ggplot2 package (version 3.4.3). Sankey plots were generated using the ggsankey package (version 0.0.99999). All boxplots display the median as the central line, the interquartile range (IQR; 25th to 75th percentile) as the box and outliers (points beyond 1.5 times the IQR) as dots outside the whiskers. Both line plots with error bands and bar plots with error bars display mean values, with the error bands and error bars representing the standard deviation above and below the means. To test for a statistically significant difference in F1 scores of CNS classes across core and expanded models, two-sided Wilcoxon signed-rank tests were used. Accuracies and F1 scores were calculated using the caret package (version 6.0-93). Gap statistics were calculated using the clusGap function from the cluster package (version 2.1.4), initialized with five configurations, considering up to five clusters and using ten bootstrap samples. No statistical methods were used to predetermine the sample size. The experiments were not randomized, and the investigators were not blinded to allocation during experiments and outcome assessment. However, our analytical pipeline followed uniform criteria applied to all samples, allowing analysis of our data in an unbiased manner. Patients who did not sign the informed consent documents, whose legal capacity to consent was unclear or whose ability to consent was questionable due to, for example, neurocognitive deficits, were excluded from the study.

### Color coding

CNS tumor classes were colored according to Capper et al.^5^, where methylation classes were grouped by histology and colored accordingly in similar shades. MZ CNS classes were colored using a shade representative of the constituting CNS tumor classes.

### MZ CNS classes—a dynamic approach to brain tumor classification

Brain tumors require a precise classification for effective treatment, a challenge given their heterogeneity. DNA methylation profiling offers a solution and has become a part of the WHO 2021 CNS tumor classification^4^. The initial system by Capper et al. (2018), analyzing 2,801 CNS tumor and control biopsies, is comprehensive yet limited to 91 methylation classes^5^. These classes combine various tumor types, grading and molecular data; sometimes, they are based solely on epigenetic analysis and have unclear relationships to existing diagnostic categories. The authors also identified eight larger groups of histologically and biologically closely related tumor classes within the 91 methylation classes in which most misclassification errors occurred and termed these groups ‘Methylation Class Families’ (MCFs).

Hollon et al.^3^ proposed a hierarchical diagnostic model tailored for intraoperative use, focusing on real-time differentiation of intraoperatively obtained biopsies via stimulated Raman histology^3^. This model prioritizes key distinctions, such as tumoral versus. non-tumoral tissue, for surgical decisions by employing a tree-like structure for classification. It focuses on diagnostic relevance and surgical need, covering 13 CNS tumor and control groups rather than aligning with the WHO classification.

Since 2018, methylation classification for CNS tumors has advanced—with the Heidelberg classifier (DKFZ Brain Classifier 12.8), trained on over 100,000 Illumina Methylation microarrays, now identifying 184 classes. However, due to inaccessible training data, recent developments still depend on the foundational dataset released by Capper et al.^5^.

Therefore, MethyLYZR introduces a flexible classification approach, anticipating that future intraoperative systems will incorporate a broader dataset spectrum, adhering to a hierarchical taxonomy for precise and tailored intraoperative decisions. It combines granular methylation classes, MethyLYZR classes (MZ classes) and a therapeutical decision-driven hierarchy. MZ classes offer simplified classification by consolidating groups with common origins or molecular profiles (Supplementary Table 1). The structure begins with binary distinctions (non-diagnostic versus diagnostic tissue) and progresses to detailed classifications based on cell type and molecular features, represented as leaves in a hierarchical tree. Methylation classes are systematically organized by cell type and molecular characteristics, culminating in representing all 91 classes as leaves in the tree. MZ classes are defined as either hierarchical nodes or, when clinically pertinent, as specific methylation classes (CNS classes).

In such a dynamic system, individual diagnostic decisions—for example, proposed by prospective clinical studies—could be specifically encoded. For example, a prospective clinical study by Drexler et al.^9^ based on methylation array profiling suggested that a maximized extent of resection (EOR) provides some survival benefits to patients with GBM RTK I and RTK II methylation subclasses but not to those with a GBM MES subclass. Therefore, an adapted MZ class system could guide prospective intraoperative clinical studies by assigning the clinical treatment node ‘maximal EOR’ to the GBM RTK I and GBM RTK II classes and ‘minimal EOR’ to GBM MES.

This hierarchical classification system, at this stage, is a purely conceptual model that demonstrates how a dynamic classification system could be adapted to specific clinical and translational needs while acknowledging the limitations of the current methylation-based classification system based on a limited training dataset (Supplementary Fig. 5). Similarly, in the context of the present study, the MZ classes represent pragmatic reference points for the evaluation of the performance of the Bernoulli naive Bayes framework with simulated and actual nanopore data and the comparison of results across different classification algorithms—for example, Bayesian classification versus deep neural networks.

Notably, the MZ classes are not intended to replace or contradict the existing neuropathological systems, such as the WHO 2021 classification system, but, rather, to complement and enhance them. Future iterations of MZ classes will require refinement and validation with emerging methylation classes and datasets, aligning them with clinical trials and evolving treatment strategies. Such systems, including those by Hollon et al.^3^ and MethyLYZR, will leverage healthcare opportunities and expert consensus to determine the best treatment approaches for specific cancers. We expect similar taxonomic approaches to become part of clinical practice guidelines, integrating diagnosis and treatment decisions to aid neurosurgeons, neuropathologists and neuro-oncologists.

### Clinical Demonstrator experimental workflow

#### Overview

Developing and implementing highly integrated workflows such as the Clinical Demonstrator experiments is an interdisciplinary challenge in translational medicine. Given the less than 1-h timeline for the clinical validation effort, every step is tightly coupled, necessitating close collaboration among neurosurgical teams, molecular biologists, bioinformaticians and neuropathologists. In this tightly coupled process, each step depends heavily on the previous one, and deviations or delays can disrupt the entire process (Supplementary Fig. 6).

This includes:

1. **Laboratory setup:** ensuring the laboratory is specifically tailored and equipped for the required tasks, including necessary equipment and reagents.
2. **Ethical, legal and scientific framework:** establishing a rigorous framework to ensure that all procedures comply with ethical standards, legal requirements and scientific integrity, including obtaining informed consent from patients.
3. **Multidisciplinary integration:** Coordinating among different professional teams, ensuring that everyone is well informed and aligned with the project goals and timelines.
4. **Surgical planning:** planning of the surgical procedure to ensure the timely and targeted collection of brain tumor biopsies from regions of the cancer with high tumor cell content.
  The overview then outlines the sequentially dependent intraoperative MethyLYZR process, illustrating the following critical steps to be performed within less than 1 h.
5. **Operation:** the actual surgical procedure where neurosurgeons obtain fresh brain tumor biopsies.
6. **Biopsy handling:** proper handling of the biopsy samples immediately after surgery to ensure their integrity, involving preparing the samples for DNA extraction and the separation of samples for the experimental workflow as well as for the routine clinicopathological workup.
7. **DNA extraction and library preparation:** DNA must be extracted from biopsy samples efficiently and in the shortest possible timeframe. Preparing nanopore sequencing libraries from the extracted DNA with optimized sequencing protocols for high pore occupancy is crucial for rapid, high-quality sequencing results.
8. **DNA sequencing:** using nanopore technology to sequence the DNA and identify methylation marks.
9. **Classification**: applying the MethyLYZR algorithm to the sequencing data for brain cancer classification, involving complex bioinformatics analyses to interpret the data and provide a diagnosis.
  Finally, contextualization and data integration as the final step after the experiment was performed for the scientific analysis of the results obtained in a clinical demonstrator experiment, which can take up to several weeks due to the currently still protracted nature of multi-modal routine clinical workup:
10. **Data integration:** integrating various data types obtained from patient characteristics, clinical information systems, conventional molecular assays, neuropathology and other analyses, which often depend on the tumor type identified in the integrated clinical neuropathological workup.

In the following, we share and contextualize, based on our experience with this study, each of the numbered steps to foster replication and improvement of the set of methods that we developed and described.

### Laboratory setup

The laboratory was set up in the surgical tract of the UKSH Neurozentrum at Campus Kiel, only two hallways from the operating rooms. The room was previously used for intraoperative rapid frozen section neuropathology. As this intraoperative service cannot currently be offered at the UKSH in Kiel, we set up the intraoperative sequencing laboratory there. Two desks or working areas are necessary—one for the molecular workflow and one for the computer setup (Supplementary Figs. 7 and 8).

**Equipment for the molecular workflow**

1. TissueLyzer LT (Qiagen, cat. no. 85600)
2. TissueLyzer LT Adapter (Qiagen, cat. no. 69980)
3. ThermoMixer C (Eppendorf, cat. no. 5382000015)
4. NanoDrop One (Thermo Fisher Scientific, cat. no. ND-ONE-W)
5. Precision scale, for example, Steinberg SBS-LW-300A (Expondo, cat. no. EX10030053)
6. Thermocycler, for example, Biometra TAdvanced Twin 48 (Analytik Jena, cat. no. 846-2-070-212)
7. Centrifuge capable of 20,000*g*, for example, Eppendorf centrifuge 5425 (Eppendorf, cat. no. 5405000719)
8. P1000, P200 and P10 pipettes, for example Eppendorf Research Plus (Eppendorf, cat. no. 3124000121, -083, -016)
9. Tabletop centrifuge, for example Greiner Bio-One Mini centrifuge (Greiner Bio-One, cat. no. 843070)
10. Fridge (4 °C)
11. Freezer (−20 °C)

Note: the equipment chosen, except the nanopore sequencers, can be replaced with equally functional equipment from other vendors. The list only describes the exact setup used in this study and is not intended to suggest specific vendors or equipment manufacturers.

**Equipment for the bioinformatic workflow**

1. Computer with monitor, RTX4090 or higher GPU, minimum 12 GB GPU RAM, 64 GB RAM Multicore CPU (12-core/24-thread Intel i7/i9 10th generation or newer processor / AMD Ryzen processor recommended), 2 TB of internal SSD storage + 6 TB external SSD storage and USB-C interface
2. Internet connection, as ONT sequencing devices in default settings need to contact ONT servers before a sequencing experiment can be started
3. MinION/P2 Solo sequencer (ONT, cat. no. MIN-101B/PRO-SEQ002)

Note: internet access can be problematic due to the operation of non-clinical computer hardware connected to the nanopore sequencer in a highly regulated and protected clinical environment. ONT can be contacted to enable the operation of nanopore sequencers without an internet connection.

### Framework

#### Ethics approval

Obtaining ethics approval was dependent on prototypical data and a well-defined setup. The study was approved by the local ethics committee of Kiel University (D443/20) and complies with the 1975 Declaration of Helsinki and its subsequent amendments.

Patients for the study were recruited from UKSH, Department of Neurosurgery in Kiel, Germany. Eligibility criteria included patients with radiologically suspected or probable relapsed brain tumors (including primary brain tumors and metastases) scheduled for cytoreductive surgery as recommended by the interdisciplinary tumor board. Amendments were obtained to subsequently include pediatric patients and also patients suffering from suspected brain metastases, as the capabilities of MethyLYZR expanded. Exclusion criteria were established to omit patients with small tumors or those located in eloquently sensitive brain regions that precluded adequate tissue collection for analysis.

Critical: adhering to applicable national laws and institutional regulatory board guidelines is essential when using human biological material. Obtaining informed consent from human subjects is mandatory. At UKSH, since 2017, all patients have been asked to grant permission for the future use of their data and biological materials for scientific research through a ‘broad consent’ framework, which ensures that patients receive adequate information and can give informed consent. Given the unique nature of our study, we opted not only to rely on the broad consent ethics approval but also to seek specific ethics approval for this particular study.

Critical: compliance with the GDPR, which became law in 2018, has become a fundamental regulatory requirement for all studies involving the collection and distribution of large datasets, including genome sequencing data, among research institutions in Europe. The GDPR ensures the protection of personal data and privacy for individuals within the European Union. For our study, this means implementing stringent data protection measures, obtaining explicit consent from participants and ensuring transparency about how their data will be used and shared. Additionally, it has become standard practice to establish data-sharing and processing agreements that adhere to the rules set out by the GDPR. Data anonymization and secure data transfer protocols are essential to comply with these standards. Although laws and regulations may vary globally, the GDPR has also become relevant for global consortia when European partners are involved.

Note: managing patients’ expectations after going through the informed consent information was sometimes difficult, given the potential and tangible impact of a diagnostic tool with more rapid diagnostic feedback compared to conventional, integrative diagnostic workflows. However, at the preclinical and experimental stage, more consideration and care must be exerted when using results to inform clinical decisions or when the individual results are communicated to the patients outside a clinical trial.

Caution: obtaining informed consent from neurocognitively impaired patients who do not yet have established legal guardians was sometimes challenging. To address this, we worked with psychiatric consultation services to evaluate whether informed consent could be obtained despite the neurocognitive impairment, which is common among patients with brain tumors. This collaboration proved to be very helpful.

#### Legal

ONT equipment, software such as MinKNOW, consumables such as flow cells and reagents such as library kits are currently designated for research use only. ONT products are currently not intended for health assessments or for diagnosing, treating, mitigating, curing or preventing any disease or condition. As of this writing, clinical-use, CE-IVD-marked consumables and equipment have been announced by ONT but are not yet commercially available. Using ONT materials in a clinical setting currently violates the terms and agreements with ONT, which are required for obtaining these products. Additionally, any software associated with the described workflow in a routine clinical setting must be evaluated based on local laws and regulations before its use in the clinic.

Note: MethyLYZR, if applied in routine clinical care, would likely be classified in Europe as a Medical Device Software (MDSW) under the MDCG 2019-11 guidance on qualification and classification of software according to Regulation (EU) 2017/745 (MDR) and Regulation (EU) 2017/746 (IVDR). This guidance specifies that software intended for a medical purpose, such as diagnosing or predicting disease, is considered a medical device.

Under the MDCG 2019-11 guidelines, software qualifies as a medical device if it is intended to be used for medical purposes as defined by the MDR or IVDR. This includes software that either drives or influences a medical device or operates independently with its own medical purpose, such as diagnosis, monitoring or treatment of diseases. Because MethyLYZR is used for brain cancer classification through methylation analysis, it meets the criteria for being classified as in vitro diagnostic (IVD) medical device software.

Moreover, according to Rule 11 of the MDR, which specifically addresses the classification of MDSW, nearly all MDSW is classified as at least Class IIa, depending on the risk associated with the information it provides and the potential impact on patient care. Therefore, MethyLYZR would likely fall under this classification due to its role in providing critical diagnostic information that could influence clinical decisions. However, it is important to note that definitive classification would require a more detailed review by regulatory experts and discussions with regulatory authorities.

Consequently, this study is a purely preclinical scientific study; no information from the MethyLYZR results could be shared with the clinical teams, patients or caregivers.

Note: this sometimes posed ethical and also psychological challenges for the clinical teams, who all deeply care for the well-being of their patients. Getting apparently accurate results from MethyLYZR within such a short timeframe while having to wait for the conventional results for days and sometimes weeks could lead to ethical and also psychological conflicts because of the perceived ‘red tape’ associated with the outlined legal boundaries, in which the scientific study has to operate necessarily. Here, leadership and open communication with all involved in such patient-centric preclinical research was key to ensuring acceptance of the current situation.

#### Scientific

Clear goals and hypotheses must be established for the future scientific exploration of intraoperative sequencing. In this case, Clinical Demonstrator experiments were designed to explore the feasibility of this approach even with shorter timeframes enabled by novel bioinformatic methods of live tumor diagnosis from sparse epigenomic data.

Intraoperative methylation classification is an enormously powerful technology capable of providing differential diagnostic information for potentially several hundred cancer entities. Future prospective and rigorous studies will be needed to validate the combination of intraoperative methylation classification as a potential means to choose differential treatment modalities. Currently, no firm prospective evidence is established that different surgical approaches to certain brain tumor entities diagnosed based on methylation profiles are beneficial to the patient, although suggestive evidence has emerged^9^.

### Multiprofessional integration

#### Experience from the Kiel site

Effective communication and coordination among the diverse multiprofessional groups involved in the Clinical Demonstrator experiments were crucial for the project’s success, particularly in Kiel, where no intraoperative frozen rapid section diagnostics were available at the start of the project. The multidisciplinary project development required close interaction among neurosurgeons, nurses, technicians, molecular biologists and bioinformaticians. A key strategy was informing the surgical team about the planned experiments during the early morning surgery conference. This ensured timely biopsy retrieval and aligned the surgical procedures with the experimental needs.

To initiate this workflow in the setup phase, having a dedicated neurosurgeon who was not performing the surgery and managing the experiment was helpful. This neurosurgeon coordinated among stakeholders and ensured that the workflow could be implemented frictionlessly. This role included overseeing the biopsy retrieval process, coordinating with the laboratory team and ensuring that the samples were processed without delay.

Note: given the dynamic nature of surgical scheduling, frequent rescheduling of surgeries was common, especially when emergency trauma patients required the same operating rooms as the neuro-oncologic surgeries. To mitigate the impact of these rescheduling events, having initially the molecular and bioinformatic scientists on standby was helpful. This approach provided the flexibility to accommodate unexpected changes and ensured that the experiments proceeded without interruptions.

#### Experience from the Oslo site

Samples harvested for intraoperative frozen section analysis were split in two. One sample followed the routine frozen-section workflow, whereas the other part was placed in lysis buffer (Qiagen) in the operating room before transport to the analytical laboratory, typical transport time being 5–24 min. No other adaption or preparation was needed for the surgical team, except for the operating room nurse to split samples.

Overall, the success of the Clinical Demonstrator experiments depended on multiprofessional planning, clear communication and the dedicated efforts of all involved professionals. This collaborative approach allowed the teams to navigate the complexities of the clinical environment.

Perspective: We expect that, for a more routinely conducted intraoperative sequencing workflow, a single technician will suffice to enable POC intraoperative sequencing for two neuro-oncology operating rooms during a shift. Automation will further increase the throughput of this method and scalability in the clinical setting.

### Surgical planning

#### Experience from the Kiel site

A histologic diagnosis is required for optimal treatment of patients with brain tumors. This can be accomplished either at the time of surgical resection or with a stereotactic biopsy. Biopsy alone is used when the lesion is not amenable to resection, a meaningful amount of tumor tissue cannot be removed or the patient’s overall clinical condition will not permit surgery. The favored initial treatment for brain tumors within accessible locations is resection. Maximal resection with preservation of neurologic function is an important goal in the initial management of patients with brain tumors, and the extent of surgery must be balanced with the preservation of neurologic function.

#### Preoperative imaging

MRI with contrast is the optimal study for the evaluation of brain tumors. Standard sequences at the Kiel site included to characterize brain tumors include T1 and T2, fluid-attenuated inversion recovery (FLAIR), gradient-echo/susceptibility, diffusion-weighted imaging and post-contrast T1-weighted images. On MRI, high-grade gliomas are typically hypointense on T1-weighted images and enhance heterogeneously after contrast administration.

Note: MRI sequences for the characterization of brain tumors vary among sites and neurosurgical departments.

Note: preoperative functional MRI and diffusion tractography may be used to optimize tumor volume definition and minimize operative injury to eloquent areas by allowing preoperative definition of affected and normal brain areas and functional mapping of brain tissue. In addition to localization of functional cortical areas such as motor cortex via functional MRI, diffusion tensor imaging (DTI) enables the visualization of subcortical tracts that carry eloquent task information from speech, motor and visual pathways.

For neurosurgical biopsy procedures, precise planning and execution are essential for the classification of brain tumors. Biopsies were strategically collected using image-guided neurosurgery with the Brainlab system. This technology enables surgeons to target the parts of the brain tumor directly, which is most likely to yield optimal diagnostic evaluation. This is often in regions at the border of contrast enhancement or areas of decreased diffusion. Optimal sampling is critical for obtaining the high tumor cell content necessary for effective methylation classification^45^. This planning ensures that the biopsy process is the initial step immediately after reaching the CNS neoplasm in an open craniotomy procedure, aiming to achieve a classification result within 1 h.

### Surgery

#### Intraoperative techniques

For deep-seated or multifocal tumors, the combined use of computerized imaging and stereotactic devices has allowed neurosurgeons to perform deep brain biopsies with accurate tumor localization. Frameless stereotaxy establishes a computerized link between the preoperative three-dimensional tumor volume and the surface landmarks of the patient. This link permits the neurosurgeon to be aware of the three-dimensional position of surgical instruments within the intracranial space during the biopsy based on preoperative imaging. For brain tumors that contain both enhancing and non-enhancing components, tumor biopsy should target the enhancing areas to obtain diagnostic tissue that is representative of the highest-grade portion of the tumor (Supplementary Fig. 10).

Preoperative magnetic resonance DTI data are loaded into a neuro-navigation system. This system aligns the patient’s anatomy with the imaging data, allowing the surgeon to navigate accurately. Augmented reality (AR) technology overlays digital information, such as tumor boundaries and fiber tracts, onto the surgeon’s field of view (Supplementary Fig. 10). This can be achieved through AR screens on the microscope field, providing a real-time, immersive visualization. The surgeon begins the tumor resection, guided by the navigation system and AR overlays. The AR displays the tumor and critical brain structures, helping the surgeon avoid vital areas. The navigation system continuously updates, showing the precise location of surgical instruments relative to the malignancy and important fiber tracts.

Note: several further intraoperative techniques (awake craniotomy and intensity-modulated radiation therapy) are frequently used to improve the extent of surgical resection while minimizing collateral damage to the normal brain. Despite these advances in surgical techniques, local recurrences are frequent, even in patients undergoing an apparently complete removal of the tumor. High-grade gliomas are characterized by poorly defined tumor margins with infiltration of neoplastic cells along white matter fibers and the perivascular spaces, which can extend well beyond the tumor margin as defined by the surgeon or by radiographic studies. Unfortunately, methylation-based classifiers perform poorly at this stage with biopsies with low tumor cell content.

### Biopsy handling

The transfer process of biopsies to the intraoperative sequencing laboratory in Kiel was considered crucial and has been optimized by establishing a POC laboratory adjacent to the operating room. The daily surgical planning meeting plays a pivotal role in this process, involving neurosurgeons, nursing staff and laboratory technicians, all briefed about the fresh biopsy collection to ensure its immediate handling and transfer. The sample for molecular analysis is placed in sterile Ringer’s lactate solution to maintain tissue viability and avoid degradation in the short timeframe for intraoperative sequencing. This handling ensured the integrity of the biopsies for sequencing, although we observed substantial degradation of DNA after 6–8 h in this solution.

Caution: in our experience, placing the biopsy in formaldehyde solutions is incompatible with the rapid sequencing adaptor chemistry required for intraoperative methylation classification. Even though workflows for nanopore sequencing of formaldehyde-fixed brain tumor biopsies have been reported^32^, these rely on ligation library chemistries, which more effectively remove the enzyme-degrading formaldehyde molecules.

### Molecular workflow

The following molecular workflow describes the steps in setting up an intraoperative sequencing workflow. Moreover, it contains a detailed list of reagents and consumables at a clinical site. To enable fast processing of sample material, we highly suggest that users focus their special attention on the pre-processing section for optimal results.

1. DNA extraction of fresh brain tumor specimens usually obtained from a standard craniotomy
2. DNA quantification and quality assessment using a NanoDrop instrument
3. Preparation of a rapid sequencing library to be used in conjunction with PromethION P2 Solo sequencer
4. Priming of a PromethION flow cell for sequencing

Consumables

- 1.5 ml of DNA LoBind tubes (Eppendorf, cat. no. 30108051)
- Petri dish 150 × 20 mm (Sarstedt, cat. no. 82.1184.500)
- Scalpel, for example Feather disposable scalpel no. 21 (Feather, cat. no. 02.001.30.021)
- 1,000-µl pipette tip, for example Sarstedt filter tip 1,000 µl (Sarstedt, cat. no. 70.3050.255)
- 0.2-ml PCR tubes, for example Biozym PCR SoftTubes (Biozym Scientific, cat. no. 711080)
- 300-µl, 10-µl pipette tips, for example SurPhob 300/10 µl of XL (Biozym Scientific, cat. no. VT0250/VT0200)
- PromethION or MinION flow cell (ONT, cat. no. FLO-MIN114/FLO-PRO114M)

Reagents

- Ringerʼs solution (133 mmol l^−1^ sodium chloride (NaCl), 1.34 mmol l^−1^ potassium chloride (KCl), 2.76 mmol l^−1^ sodium hydrogen carbonate (NaHCO_3_) and 1.25 mmol l^−1^ calcium chloride (CaCl_2_))
- QIAamp Fast DNA Tissue Kit (Qiagen, cat. no. 51404)
- Dulbecco’s PBS, DPBS 1× (Life Technologies, cat no. 14190144)
- Nuclease-free water (Life Technologies, cat. no. 10977035)
- Molecular-grade ethanol (non-denatured) (96–100%; Sigma-Aldrich, cat. no. 1.07017.2511)
- 2-Propanol (≥100%; Carl Roth, cat. no. 6752.3)
- Rapid Sequencing Kit V14 (ONT, cat. no. SQK-RAD114)

#### Pre-processing steps

- Set a thermomixer to 56 °C.
- Supplement wash buffer AW1 and AW2 of the QIAamp Fast DNA Tissue Kit with 96–100% molecular-grade ethanol (Supplementary Table 22).
- Supplement wash buffer MVL of the QIAamp Fast DNA Tissue Kit with ≥99.8% molecular-grade 2-propanol (Supplementary Table 22).
- Prepare a master digestion buffer mix in a 1.5-ml Eppendorf tube for three reactions (MMX 3) and mix by pipetting (Supplementary Table 23).
- Transfer 265 µl of digestion buffer mix into an empty tissue disruption tube. Repeat the previous step to obtain two pre-filled tissue disruption tubes.
- Tare the scale using an empty blue cap of a tissue disruption tube provided with the QIAamp Fast DNA Tissue Kit.
- Set the centrifuge to 20,000*g* and 1 min at RT.
- Program a thermalcycler: 30 °C for 2 min 15 s and then 80 °C for 1 min.
- Place the reagents of the rapid sequencing kit (SQK-RAD114) on ice.
- Program a TissueLyser LT: 45 Hz, 2 min.
- Add 200 µl of nuclease-free water to a fresh 1.5-ml Eppendorf tube and place on a thermomixer at 56 °C.
- Remove a PromethION flow cell from the fridge and equilibrate to RT for 20–30 min.
- Boot a sequencing computer, attach the P2 Solo and open the MinKNOW software.
- Open a terminal and source the virtual environment.
- Set up the MethyLYZR command with the appropriate arguments and take the same sample name (-s Parameter) as the Sample ID in MinKNOW.

Note: we recommend performing a flow cell quality check (QC) before use. PromethION flow cells that show fewer than 5,000 active pores do not pass ONT’s quality standards and will be replaced.

#### DNA extraction

- The obtained brain biopsy should be provided in Ringerʼs solution, creating an isotonic environment for proper cellular stability.
- Place the specimen into a Petri dish and cut pinhead-sized fragments of vital tumor tissue (Supplementary Fig. 11).
- Weigh an empty cap of a tissue disruption tube by using a precision scale and press the tare button.
- Remove the empty cap of a tissue disruption tube from the scale and place the tissue fragment into the cap.
- Repeat the previous step to obtain a total of two reactions.
- Weigh the samples and adjust the tissue weight to 10–15 mg.

Note: the Qiagen Fast DNA Tissue Kit enables DNA extraction from up to 25 mg of fresh, frozen or stabilized tissue material using a combined mechanical, chemical and enzymatic lysis. Most importantly, the kit also allows for high-quality DNA extraction of challenging samples, such as brain tissue, within a short time. The outlined protocol has extensively been tested with 5–25 mg of brain tissue material from fresh, snap-frozen and preserved (Biomatrica, DNAgard Tissues and Cells) samples. However, we found that 10–15 mg of vital tumor tissue generally yields sufficient DNA for the ONT’s rapid library preparation protocol. Note that, additionally, the incubation time for the lysis reaction can be reduced from 10 min at 56 °C to 7 min if less tissue material is used.

- Remove the cap from the precision scale and screw it on top of a pre-filled tissue disruption tube.
- Quickly spin the tubes by using a tabletop centrifuge.
- Place the tubes in a TissueLyser LT instrument and disrupt the samples at 45 Hz for 2 min at RT.
- Quickly spin the tubes using a tabletop centrifuge.
- Place the samples in a thermomixer and incubate for 7 min at 56 °C with 1,000 rpm.
- Add 265 µl of buffer MVL (containing isopropanol) to each of the tissue disruption tubes and mix 10× by pipetting.

Note: adding buffer MVL to the lysate combined with pipette mixing results in DNA precipitation that can be visually monitored. Samples containing large amounts of DNA can even form a slightly viscous solution, indicating sufficient extraction with high DNA content.

- Apply the lysate to a fresh QIAamp mini spin column.
- Centrifuge the samples for 1 min at 20,000*g* for 1 min at RT.
- Replace the collection tube and add 500 µl of buffer AW1.
- Centrifuge the samples at 20,000*g* for 30 s at RT.
- Replace the collection tube and add 500 µl of buffer AW2.
- Centrifuge the samples at 20,000*g* for 30 s at RT.
- Replace the collection tube and centrifuge at 20,000*g* for 2 min at RT.
- Meanwhile, blank a NanoDrop device using 2 µl of nuclease-free water.
- Place the spin column into a 1.5-ml DNA LoBind tube.
- Apply 50 µl of pre-heated (56 °C) nuclease-free water to the spin column.
- Incubate the samples for 1 min at RT.
- Centrifuge the tube at 20,000*g* for 1 min at RT to elute the DNA.

Note: elution buffers, such as TE, usually contain 10 mM EDTA and can have detrimental effects on both the rapid library preparation and sequencing performance. We recommend using nuclease-free water (pH 7–8) instead for optimal results. However, once intraoperative sequencing has been performed, it is highly advised to supplement the remaining DNA sample with TE to prevent DNA degradation from hydrolysis and to allow long-term storage.

#### DNA quantification and quality assessment

- Quantify 2 µl of each eluate on a NanoDrop instrument.
- Select one of the samples based on DNA quality (A260/A230 and A260/A280) and quantity.
- Transfer 100–150 ng of the DNA sample into a fresh 0.2-ml PCR tube.
- Adjust the volume to 10 µl with nuclease-free water and proceed with the rapid sequencing protocol.

Note: rapid DNA quantification and quality assessment are pivotal in an intraoperative sequencing setup. A spectrophotometer, such as the NanoDrop One, enables DNA quantification within 10 s and provides valuable information about the DNA quality based on absorbance maxima at 230 nm, 260 nm and 280 nm. The QIAamp Fast DNA Tissue Kit results in high-quality DNA samples based on a characteristic A260/A280 ratio of 1.8–1.85 for almost all samples investigated in this study. However, it should be noted that we observed some variability in the A260/A230 ratio among brain tumor samples, especially if poor sample material, such as necrotic tissue, was used. Using fresh or immediately snap-frozen tissue material is, therefore, highly recommended.

#### Preparation of the sequencing library

Note: we recommend using the rapid sequencing kit (SQK-RAD114; Supplementary Table 24) to prepare sequencing libraries in an intraoperative setting to obtain the best results. Besides providing a simple and rapid protocol with minimal hands-on time, it maximizes the number of sequencing reads that can be used for bioinformatic analysis, leading to the fastest turnaround time for tumor prediction using MethyLYZR. Equally, the rapid barcoding kit (SQK-RAD114.24) can also be used if scaling out throughput is of particular interest. It provides the same rapid turnaround time while allowing multiple samples to be processed simultaneously. However, the barcoding protocol generally tends to result in lower amounts of sequencing reads usable for brain tumor classification due to a fraction of unclassified reads due to failed barcode assignment. The protocol described below outlines the rapid sequencing approach without barcoding.

- Add 1 µl of FRA to the diluted DNA sample.
- Start the PCR program.
- Thoroughly pipette mix the sample and place it into the thermalcycler.
- Meanwhile, place the PromethION flow cell into the P2 Solo.
- Rotate the inlet valve of the PromethION clockwise to expose the inlet port.
- Draw back 30 µl of flow cell storage buffer from the inlet port.
- Prepare the priming mix by adding 30 µl of FCT to 1,170 µl of FCF.
- Resuspend the priming mix using a P1000 and apply 500 µl of priming mix to the inlet port.
- Incubate the flow cell for 5 min at RT.
- Proceed with the library preparation and aliquot 3.5 µl of ADB into a fresh 0.2-ml PCR tube.
- Add 1.5 µl of the RA to the adapter dilution buffer and mix thoroughly by pipetting.
- Remove the tagmented sample from the PCR cycler and place it on wet ice for 10 s.
- Add 1 µl of the diluted RA to the tagmented sample and thoroughly mix by pipetting.
- Incubate the sample for 5 min at RT for the attachment of the sequencing adapter.
- Complete the flow cell priming by adding 500 µl of priming mix to the inlet port.

Caution: the flow cell storage buffer contains tripotassium hexacyanoferrate and is incompatible with acids. Always wear gloves and safety goggles when handling flow cells containing storage buffer.

Critical: avoid introducing air bubbles to the sensor array, as this would damage the nanopores. Always ensure that the inlet port is filled with buffer.

- Finalize the sequencing library according to the table below (Supplementary Table 25).
- Load 200 µl of final library via the inlet port.
- Turn the valve to close the port and apply a light shield (supplied with flow cells) to protect the sensor array.
- Ensure that all sequencing parameters have been selected correctly, and hit the start button to initialize sequencing.

Caution: optimal flow cell loading requires training and pipetting skills. Prepare by training on spent flow cells after watching instruction videos.

### DNA sequencing

#### Loading of the nanopore flow cell and start of the nanopore sequencing device

MinKNOW parameter set:

1. **Sample ID:** must match the sample name given in the live_classifier.py command.
2. **Sequencing kit:** select the appropriate kit.
3. **Basecalling:** basecalling with the high-accuracy model needs to be enabled.
4. **Modified basecalling:** modified basecalling needs to be enabled.
5. **Modified base context:** this needs to be set to 5mC and 5hmC.
6. **Alignment:** alignment needs to be enabled. Ensure that the alignment reference genome file matches the reference stated in the live_classifier.py parameters.
7. **Basecalled reads:** ensure that BAM files are written to disk every minute.
8. **Template:** all settings can be saved as a template for future use.
9. **Start sequencing run:** start the sequencing run after proper flow cell priming and loading the library.

### Classification

#### Hardware (computer)

For optimal performance during live classification, a suitable hardware setup is essential. MinKNOW, designed by ONT, handles live base and methylation calling on large datasets, which can be computationally intensive. Meeting specific hardware requirements is important for efficient operation.

This section provides the recommended hardware specifications from ONT and alternative configurations that we found to work. Adhering to these recommendations ensures live cancer classification results with live_classifier.py using MethyLYZR.

Caution: the following descriptions of computer setups and software configurations all represent only a snapshot of systems that have worked well for this task in 2023/2024. Specifically, as ONT software still undergoes further refinement, we expect the following recommendations and experiences to become outdated in the foreseeable future. We still think that this section will provide important information for any laboratory that wants to set up intraoperative live sequencing analysis in the years to come as a conceptual framework.

Recommendations from ONT. To achieve optimal performance for high-accuracy base and methylation calling:

- GPU: Nvidia RTX 4090 or higher
  - Minimum 12 GB GPU RAM
- RAM: 64 GB
- CPU: Multicore processor
  - 12-core/24-thread Intel i7/i9 10th generation or newer or AMD Ryzen processor
- Storage:
  - 2 TB internal SSD
  - 6 TB external SSD
- Interfaces:
  - USB-C interface for P2 Solo
  - USB-A 3.0 interface for MinION

Known working configurations. In addition to the recommended specifications, the following configurations are also known to work:

- Nvidia RTX 3090 Desktop GPU
- Nvidia RTX 4090 Mobile GPU

Caution: meeting these hardware requirements can ensure that your system can handle the computational demands of online high-accuracy base and methylation calling and running live classification. However, as many hardware components can affect the overall performance of such a system, online base and methylation calling cannot be guaranteed and requires thorough testing of the intraoperative compute system.

#### Hardware (sequencing devices)

##### MinION

The MinION is a mid-range sequencing device by ONT that can read DNA with up to 512 pores in parallel. The device cost is currently approximately €1,000 (June 2024).

##### PromethION 2 Solo (P2 Solo)

The P2 Solo sequencing device by ONT is a portable platform that can read DNA with up to (theoretically) 3,000 pores in parallel. The device cost is currently approximately €10,000 plus €1,000 yearly software and license fee (June 2024).

Note: PromethION flow cells do not currently offer an advantage over MinION flow cells for intraoperative sequencing workflows targeting results within 1 h after biopsy. Due to their different geometry, PromethION flow cells take considerably longer to reach maximum throughput compared to MinION flow cells. However, PromethION flow cells provide a 3–4-fold increase in overall sequencing yield after a complete run over several days.

#### Software

##### MinKNOW

MinKNOW is a software developed by ONT for controlling and managing sequencing devices such as MinION, GridION and PromethION. It handles device operation, data acquisition and preliminary analysis for sequencing workflows.

Key features

- Device control: allows starting, monitoring and controlling sequencing runs with real-time feedback.
- Data acquisition: streams data directly from the sequencing device, providing immediate access to raw reads.
- Real-time analysis: includes tools for real-time basecalling.
- Quality control: provides built-in metrics and monitoring to ensure data integrity.

Technical specifications

- Compatibility: it works with MinION, GridION and PromethION devices, although device-specific versions exist and are sometimes not identical in features.
- Operating systems: available for Windows, macOS and Linux.
- Integration: it integrates with other bioinformatics tools and pipelines for downstream data analysis.

Note: MinKNOW v.24.02.6 (OSX and Windows) and v.24.02.10 (Ubuntu) are not suitable for live classification, as they save the necessary data to disk at a minimum interval of 10 min. Earlier versions save data based on the number of reads rather than time. Subsequent versions offer an option to save data to disk every minute.

Caution: MinKNOW still regularly undergoes changes that affect compatibility with custom scripts and workflows. We test any update thoroughly for compatibility before live usage.

##### Installation

Windows installation.

1. System requirements:
  Ensure that your system meets the hardware requirements, including an Nvidia GPU (RTX 4090 or higher recommended) with a minimum of 12 GB GPU RAM.
  Ensure that you have administrative rights to install the software.
2. Download MinKNOW:
  Visit the ONT website and download the latest MinKNOW installer for Windows.
3. Install Nvidia drivers:
  Download and install the latest Nvidia drivers for your GPU from the Nvidia website.
4. Install CUDA Toolkit:
  Download and install the CUDA Toolkit from the Nvidia website. Ensure that you select the version compatible with your GPU and operating system.
5. Run the MinKNOW installer:
  Double-click the downloaded MinKNOW installer to start the installation process.
  Follow the on-screen instructions to complete the installation.
6. Verify installation:
  Start a test sequencing run to ensure that MinKNOW is using the GPU for basecalling and other computational tasks.

Linux installation.

1. System requirements:
  Ensure that your system meets the hardware requirements, including an Nvidia GPU (RTX 4090 or higher recommended) with a minimum of 12 GB GPU RAM.
  Ensure that you have administrative rights to install the software.
2. Update system packages:
  Open a terminal and update your system packages:
    sudo apt-get update
    sudo apt-get upgrade
3. Install Nvidia drivers:
  Install the latest Nvidia drivers:
    sudo add-apt-repository ppa:graphics-drivers/ppa
    sudo apt-get update
    sudo apt-get install nvidia-driver-460
    sudo reboot
4. Install CUDA Toolkit:
  Download and install the CUDA Toolkit from the Nvidia website. Follow the installation instructions specific to your Linux distribution.
5. Install MinKNOW:
  Add the ONT apt repository
    i. For Ubuntu 22:
      sudo apt update
      sudo apt install wget
      wget -O-https://cdn.oxfordnanoportal.com/apt/ont-repo.pub| sudo apt-key add -
      echo ‘debhttp://cdn.oxfordnanoportal.com/aptjammy-stable non-free’ | sudo tee /etc/apt/sources.list.d/nanoporetech.sources.list
    ii. For Ubuntu 20:
    sudo apt update
    sudo apt install wget
    wget -O-https://cdn.oxfordnanoportal.com/apt/ont-repo.pub| sudo apt-key add -
    echo ‘debhttp://cdn.oxfordnanoportal.com/aptfocal-stable non-free’ | sudo tee /etc/apt/sources.list.d/nanoporetech.sources.list
  Install GPU Version:
    sudo apt update
    sudo apt install ont-standalone-minknow-gpu-release
6. Verify installation:
  Start a test sequencing run to ensure that MinKNOW is using the GPU for basecalling and other computational tasks.

Following these steps, you can successfully install MinKNOW with GPU support on Windows and Linux systems.

##### Live classifier

Installation

This section provides a guide for setting up a Python virtual environment with the necessary dependencies for the live classification on Ubuntu Linux. It covers both native Ubuntu installations and Ubuntu running inside Windows Subsystem for Linux (WSL).

Ubuntu is the recommended distribution used by ONT. WSL allows Windows users to run an Ubuntu environment within Windows, enabling seamless integration between the two operating systems. Setting up workflows in these environments allows researchers and developers to leverage Linux tools and libraries while maintaining compatibility with Windows workflows.

This guide includes steps to install Python v.3.10, set up a virtual environment and install all required dependencies. It is applicable for both native Ubuntu systems and WSL.

Prerequisites

Before proceeding, ensure that you have administrative access to your system, as some commands require elevated privileges. Additionally, if you are using WSL, ensure that you have WSL installed and an Ubuntu distribution set up. If you have not installed WSL yet, follow the official Microsoft documentation to get started (Install WSL).

Step-by-step guide to install dependencies:

- Update package list:
  sudo apt update
- Install Python v.3.10:
  sudo add-apt-repository ppa:deadsnakes/ppa
  sudo apt update
  sudo apt install python3.10 python3.10-venv python3.10-dev
- Ensure that you have the latest version of pip for Python v.3.10:
  curl -sS https://bootstrap.pypa.io/get-pip.py| sudo python3.10
- Use the venv module to create a virtual environment:
  python3.10 -m venv liveclassifier
- Activate the virtual environment:
  source liveclassifier /bin/activate
- Ensure that pip is up to date inside the virtual environment:
  pip install–upgrade pip
- Install the necessary dependencies for the live classification script:
  pip install -r requirements.txt
  This setup ensures that your Python environment is isolated, avoiding conflicts with other projects and system-wide packages.

Usage

The main script initializes directories, sets up file watchers and processes BAM files for methylation analysis. It uses multiprocessing to handle large datasets efficiently. The key steps include reading BAM files, processing methylation data and predicting methylation classes. Results are aggregated, shown and saved for further analysis.

To run the live classifier, use the following command (be aware that the virtual environment needs to be activated first):

live_classifier.py --inputs /path/to/bam --output /path/to/output

--sample SampleID --reference hg38

This command specifies the input directory containing BAM files, the output directory for results, the sample ID and the EPIC ID reference (T2T or HG38).

Required arguments

--inputs (-i): filepath of BAM files. If MinKNOW is used, the path to the output directory will be used.

--sample (-s): name of the Sample. Needs to be the same as Sample ID in MinKNOW.

--output (-o): path to the output folder. A subfolder with the sample name will be created.

Optional arguments

--min_entries: minimum number of new CpG entries for methylation classification. The default is 1,000.

--methylation_qsore: minimum Q-Score of basecalled read to be considered in methylation classification. The default is 9.

--recursive (-r): recursively monitor subdirectories for BAM files.

--offline: run classification offline. No MinKNOW connection is needed.

--methylation_threads: number of threads used for methylation data extraction. The default is 8.

--io_threads: number of threads used for io-handling. The default is 2.

--reference: the reference for array probes (BED file linking an EPIC ID to a genomic position)—that is, ‘T2T’ or ‘HG38’.

--dev-key: developer Key for MinKNOW API. Only needed if MinKNOW is not running on the same machine—for example, WSL.

--filter: string that needs to be present in the path. Useful for filtering on barcodes. If barcode filtering is used, the filter phrase needs to be like in the MinKNOW output directory—for example, barcode01.

Usage notes

- Ensure that all dependencies are installed.
- Customize parameters such as --min_entries, --methylation_threads and --io_threads based on your computational resources and dataset size.
- For real-time analysis, the script can interface with MinKNOW API to process data as they are generated.

Starting a live classification

The command to run live_classifier.py is as follows:


live_classifier.py -i <input_directory>
--methylation_qsore <score> -r -s <sample_id>
-o <output_directory> --io_threads <num_io_threads>
--methylation_threads <num_methylation_threads>
--dev_key <device_key> --reference <HG38/T2T>


Important considerations:

1. Sample ID (-s parameter):
  The -s parameter specifies the sample ID and must match the Sample ID used in MinKNOW. This ensures consistency and proper identification of the sample throughout the analysis.
2. Device key (--dev_key parameter):
  If you are running the script in a WSL environment, you need to provide a device key using the --dev_key parameter.
  On a native Linux system, the script should connect directly to the MinKNOW software without requiring a device key.
3. Output directory (-o parameter):
  Change the -o parameter to an output directory to save prediction and methylation data. For example, -o /path/to/output_directory.
4. Input directory (-i parameter):
  The -i parameter specifies the input directory where the data are located.
  On WSL, this might be /mnt/c/data.
  On a native Linux system, this is usually /var/lib/minknow/data.
  The input path is a fallback directory if a connection to MinKNOW cannot be established.
5. If using the rapid barcoding kit (optional):
  The --filter parameter may be given to only analyze reads that correspond to the specific barcode.
  It must match the MinKNOW output subdirectory name—for example, barcode01.

Steps to run the command:

1. Setup MinKNOW and start sequencing run:
  Start MinKNOW and ensure that it is properly configured with the Sample ID matching the -s parameter.
  Wait until the target temperature is reached.
2. Run the command:
  Open a terminal and activate the virtual environment.
  Start the live classifier command.
3. Monitor the run:
  Monitor the output and logs to ensure that the script is processing data correctly.
  The prediction results are also output into the ‘System messages’ of MinKNOW, so it may be saved alongside the run report.
4. Post-run:
  After the sequencing run is stopped, the live classification script will finish the analysis and write the final methylation data and predictions to disk.

Offline classification

Below are the instructions for running an offline classification using the live_classifier.py script. Note that, in offline mode, the --dev_key parameter is not needed, and the --offline parameter must be provided.


live_classifier.py -i <input_directory>
--methylation_qsore <score> -r -s <sample_id>
-o <output_directory> --io_threads <num_io_threads>
--methylation_threads <num_methylation_threads>
--offline --reference <HG38/T2T>


Important considerations:

Sample ID (-s parameter):

- The -s parameter specifies the Sample ID.
  Output directory (-o parameter):
- Use the -o parameter to specify an output directory where the prediction and methylation data will be saved.
  For example, -o /path/to/output_directory
  Input directory (-i parameter):
- The -i parameter specifies the input directory where the data are located.
- On WSL, this might be /mnt/c/data.
- On a native Linux system, this is usually /var/lib/minknow/data.
- The input path acts as a fallback directory if a connection to MinKNOW cannot be established.
  Offline mode (--offline Parameter):
- The --offline parameter must be given to indicate that the classification is being run in offline mode.
  BAM files handling:
- BAM files will be read in chronological order.
- BAM files may come from different runs (with different run IDs). The start times in the output will be logged individually for different runs.
- Note: there is a known issue in ONT Dorado software where experiment start time offsets may be introduced based on the system locale. Be aware that start times might be incorrect in these cases.

Steps to run the command:

1. Prepare the environment:
  Open a terminal and activate the virtual environment.
2. Run the command:
  Execute the live_classifier.py command with the appropriate parameters.
3. Monitor the run:
  Monitor the output and logs to ensure that the script is processing data correctly.
4. Post-run:
  After the sequencing run is stopped, live_classifier.py will finish the analysis and write the final methylation data and predictions to disk.

**Output.** The script will generate the following output files in the specified output directory in a subdirectory named after the Sample ID:

- A log file containing the logs of the analysis
- A results CSV file containing the methylation prediction results
- A methylation data file with the .feather extension containing the processed methylation data
  Processed methylation data in Apache Arrow Feather format
- epic_id: the ID of the epigenetic marker corresponding to the Bead-Array
- methylation: the probability of methylation
- scores_per_read: the amount of CpGs with an epic_id on one read
- binary_methylation: binary value of methylation with a threshold of 0.8 being methylated (not further used)
- read_id: the ID of the read
- start_time: the start time of the read in seconds since the experiment start
- run_id: the ID of the run
- QS: quality score as reported by the basecaller
- read_length: the length of the read
- map_qs: the mapping quality score coming from minimap2

The data are sorted by start_time and then written into a feather file. Feather (https://arrow.apache.org/docs/python/feather.html) is a binary file format that is designed for efficient storage of pandas DataFrames. It allows for fast read and write speeds while maintaining complete data integrity.

Note: depending on the size of the input data and the number of threads specified, the script may require considerable computational resources.

Note: the tool is designed to work with specific reference genome versions (T2T or HG38). Ensure that the reference specified matches the reference used for alignment in the BAM files.

### Data integration

At the Kiel site, age, gender, tumor location from patient records and imaging and the integrated tumor diagnosis, including WHO grade and molecular markers, determined by the Department of Neuropathology, University Medical Center Eppendorf, were collected from the medical files of all included patients. All patient-identifying data were pseudonymized before further use for scientific purposes. If available, EPIC methylation results were collected from selected biopsies.

Note: although unfeasible in the intraoperative use case, copy number variant calling and tumor purity estimation through ichorCNA^46^ as implemented in the GLIMMERS pipeline^31^ provided valuable additional information.

Caution: sample mix-ups can affect a considerablet portion of the datasets. The incidence of sample mix-ups and mislabeled specimens in clinical studies varies significantly across different settings, with reported rates ranging from 0.39 per 1,000 to over 1%, depending on the specific type of specimen and the clinical environment. For instance, the College of American Pathologists documented mislabeling rates in various studies, showing a rate of 0.39 per 1,000 in 120 institutions^47^, 0.92 per 1,000 in 147 clinical laboratories^48^ and 1.12% in blood bank specimens from 122 clinical laboratories^49^. In terms of the cancer research setting, one study highlighted that sample mix-ups can occur in up to 3% of sequencing workflows^50^.

Note: as the sequencing results were immediately available, consistency checks between nanopore CNV results and expected CNV patterns (for example, 1p19q-deletions in oligodendroglioma) proved to be very helpful.

### Reporting summary

Further information on research design is available in the Nature Portfolio Reporting Summary linked to this article.

## Online content

Any methods, additional references, Nature Portfolio reporting summaries, source data, extended data, supplementary information, acknowledgements, peer review information; details of author contributions and competing interests; and statements of data and code availability are available at 10.1038/s41591-024-03435-3.

## Supplementary information

Supplementary Information Supplementary Figs. 1–5 with legends, Text and Protocol.

Reporting Summary

Supplementary Table Supplementary Table 1 Hierarchical structure of the MethyLYZR classes (MZ CNS classes) and mapping to the 91 CNS training classes proposed by Capper et al. Supplementary Table 2 Runtime and memory benchmarking for classifier training on a 2017 Apple iMac personal computer and a high-performance server. Supplementary Table 3 Accuracy and F1 scores for the prediction of synthetic CNS samples evaluated on the CNS classes using randomly sampled 1 to 20k CpGs. Supplementary Table 4 Accuracy and F1 scores for the prediction of synthetic CNS samples evaluated on the MZ CNS classes using randomly sampled 1 to 20k CpGs. Supplementary Table 5 Accuracy and F1 scores for the prediction of synthetic CNS samples evaluated on the MCFs using randomly sampled 1 to 20k CpGs. Supplementary Table 6 Accuracy and F1 scores for the prediction of synthetic CNS and CNS metastasis samples evaluated on the four broad classes, CNS, breast, lung and melanoma metastasis, using randomly sampled 7.5k CpGs. Supplementary Table 7 Accuracy and F1 scores for the prediction of synthetic CNS samples evaluated on the MZ CNS classes using randomly sampled 7.5k CpGs and an expanded model including CNS and metastasis classes. Supplementary Table 8 Accuracy and F1 scores for the prediction of synthetic CNS, CNS metastasis and sarcoma samples evaluated on the training classes using randomly sampled 7.5k CpGs and an expanded model including CNS, metastasis and sarcoma classes. Supplementary Table 9 Accuracy and F1 scores for the prediction of synthetic CNS samples evaluated on the MZ CNS classes using 7.5k randomly sampled CpGs and an expanded model including CNS, metastasis and sarcoma classes. Supplementary Table 10 Diagnostic and sequencing data on 75 nanopore runs from 51 patient biopsies and precise time information on 10 samples sequenced in an interoperative setup in the clinics (‘Clinical Demonstrator’; see Supplementary Video). Supplementary Table 11 Information on Illumina EPIC methylation arrays for 22 of the 75 nanopore runs. Supplementary Table 12 Sequencing statistics regarding captured model features over time for the 75 nanopore runs. Supplementary Table 13 Classification results of 75 nanopore runs using data from the first 15 min of sequencing. Supplementary Table 14 Predictions and statistics of the 75 nanopore runs over time. Supplementary Table 15 Classification results of 180 R10 nanopore runs using 7.5k CpGs from read sampling resembling 15 min of sequencing. Supplementary Table 16 Classification results of 16 PacBio samples using data from full runs. Supplementary Table 17 Statistics of classification using 2.5k, 5k and 7.5k randomly sampled CpGs from sequencing data across PacBio, ONT R9 and ONT R10 platforms. Note that no posterior threshold was applied to predictions from PacBio data due to the high sequencing accuracy. Supplementary Table 18 Classification results of 94 external nanopore sequencing runs from brain tumor samples, with varying cutoffs for tumor purity as measured by ACE. Supplementary Table 19 Classification results of 41 cfDNA nanopore runs from CSF liquid biopsies. Supplementary Table 20 Statistics of classification across MethyLYZR, Sturgeon and nanoDx model for synthetic CNS samples using 5k, 7.5k and 10k randomly sampled CpGs evaluated on Sturgeon’s summarized classes. Supplementary Table 21 Classification results of Sturgeon and nanoDx for 75 nanopore runs using data from the first 15 min of sequencing.

Supplementary Video This video is a composite of ten Clinical Demonstrator runs with timings for DNA extraction and library preparation (see also Supplementary Table 10). All libraries were run on multiple flow cells (2–4) to validate consistent classification across runs. For all runs, the correct diagnosis was returned using data from 15 min of sequencing. This video has been deposited at https://zenodo.org/records/13236097.

## Data Availability

Sequencing data obtained via Nanopore or PacBio sequencing have been deposited in the European Genome-Phenome Archive (study accession no. EGAS50000000559, Nanopore R9 dataset accession no. EGAD50000000832, Nanopore R10 dataset accession no. EGAD50000000791 and PacBio dataset accession no. EGAD50000000798). The methylation values have been deposited in feather format at 10.5281/zenodo.13236096 (ref. ^51^). The Supplementary Video has been deposited at 10.5281/zenodo.13324497 (ref. ^52^). Previously published 450k or EPIC arrays were used for classifier training and evaluation: brain normal and cancer data (GSE90496 and GSE109379), metastasis data (GSE108576) and sarcoma data (GSE140686). DNA methylation data for purity analysis were previously published by Djirackor et al.^13^ and were reprocessed for this study as described above. DNA methylation data for the liquid biopsy analysis were directly obtained from the authors of the original study^32^ upon personal communication.

## Code availability

MethyLYZR code with a pre-trained model and test data, as well as documentation, is available at the following link: https://github.com/marasteiger/MethyLYZR.
