# Supplementary material for: Rapid brain tumor classification from sparse epigenomic data
Source: Nat Med. 2025 Feb 28;31(3):840–8. doi: 10.1038/s41591-024-03435-3 (PMC11922770; doi:10.1038/s41591-024-03435-3)
Supplement: Supplementary file 1 — Supplementary Figs. 1–5 with legends, Text and Protocol. [file 41591_2024_3435_MOESM1_ESM.pdf]

nature medicine

Supplementary information https://doi.org/10.1038/s41591-024-03435-3

Rapid brain tumor classification from sparse epigenomic data

In the format provided by the authors and unedited

1 Supplementary Figures

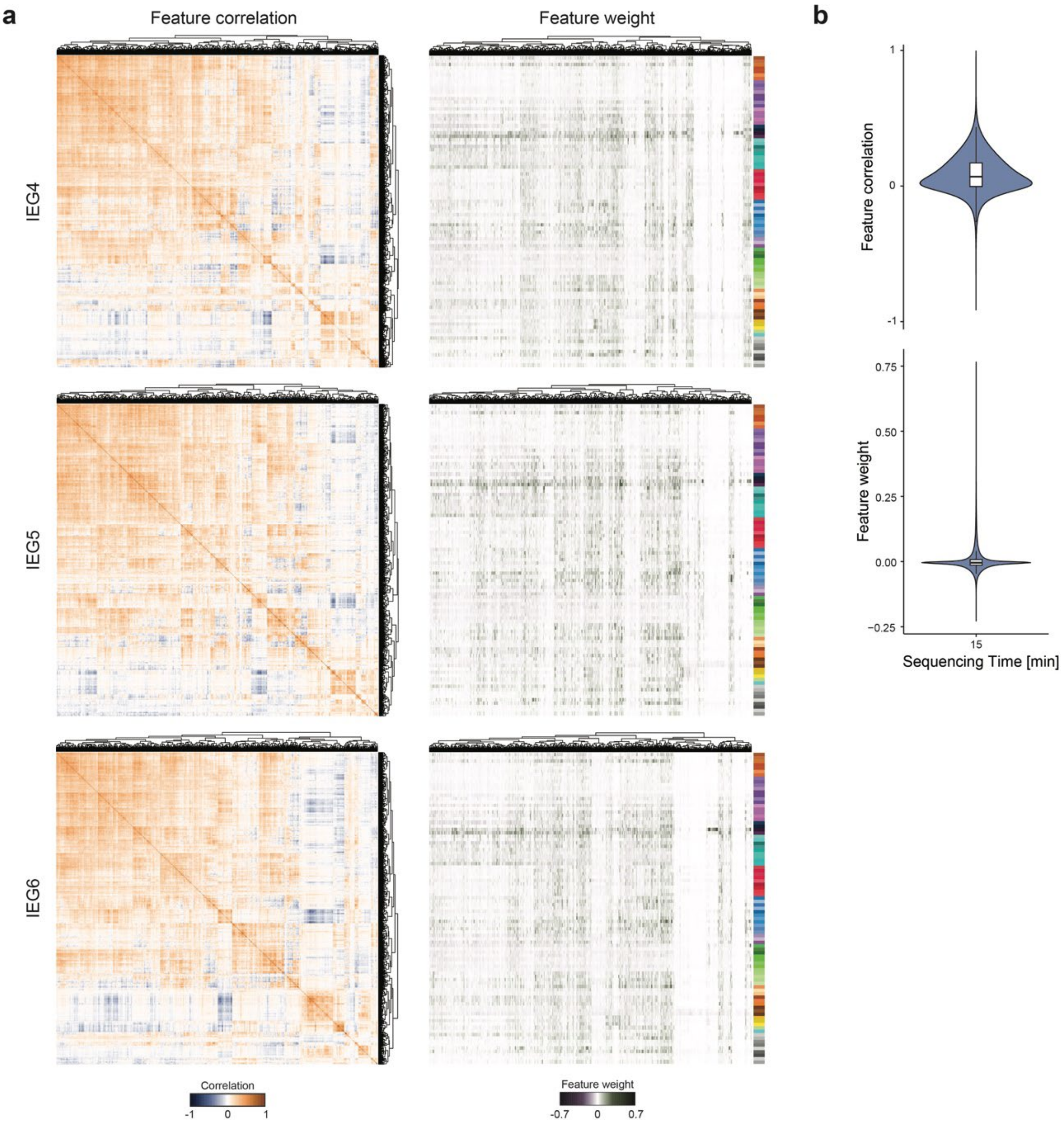

Supplementary Fig. 1: (a) Correlation of methylation rates from features obtained within 15 minutes of sequencing of three samples (left) and respective feature weights (right). (b) Summary of the distribution of all correlations and feature weights from all R9 samples obtained within 15 minutes of sequencing and respective feature weight distribution. Boxplots display the median as the central line, the interquartile range (IQR; 25th to 75th percentile) as the box, and 1.5 times the IQR as the whiskers.

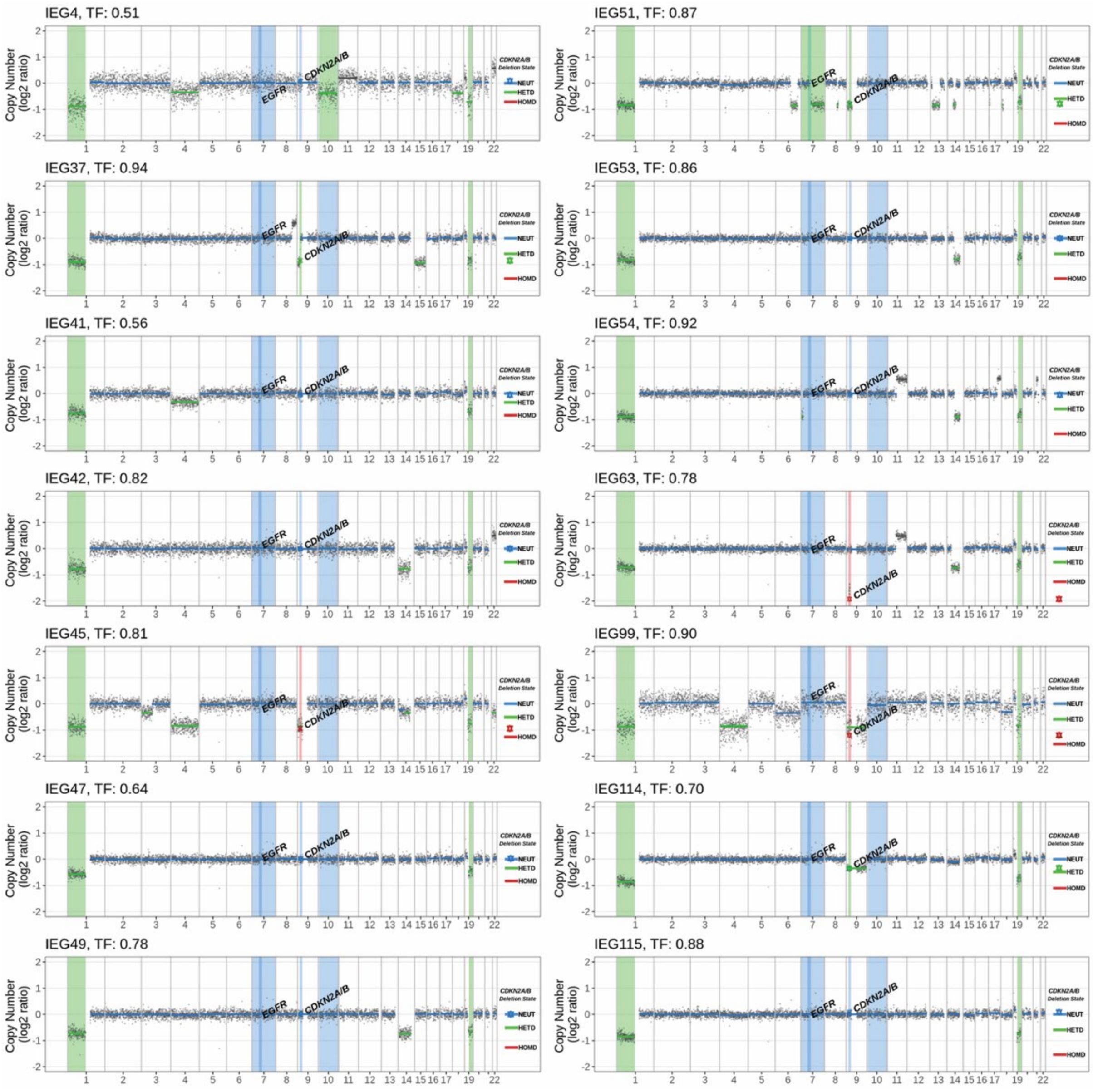

Supplementary Fig. 2: Copy number variation plots of O IDH samples obtained using GLIMMERS.

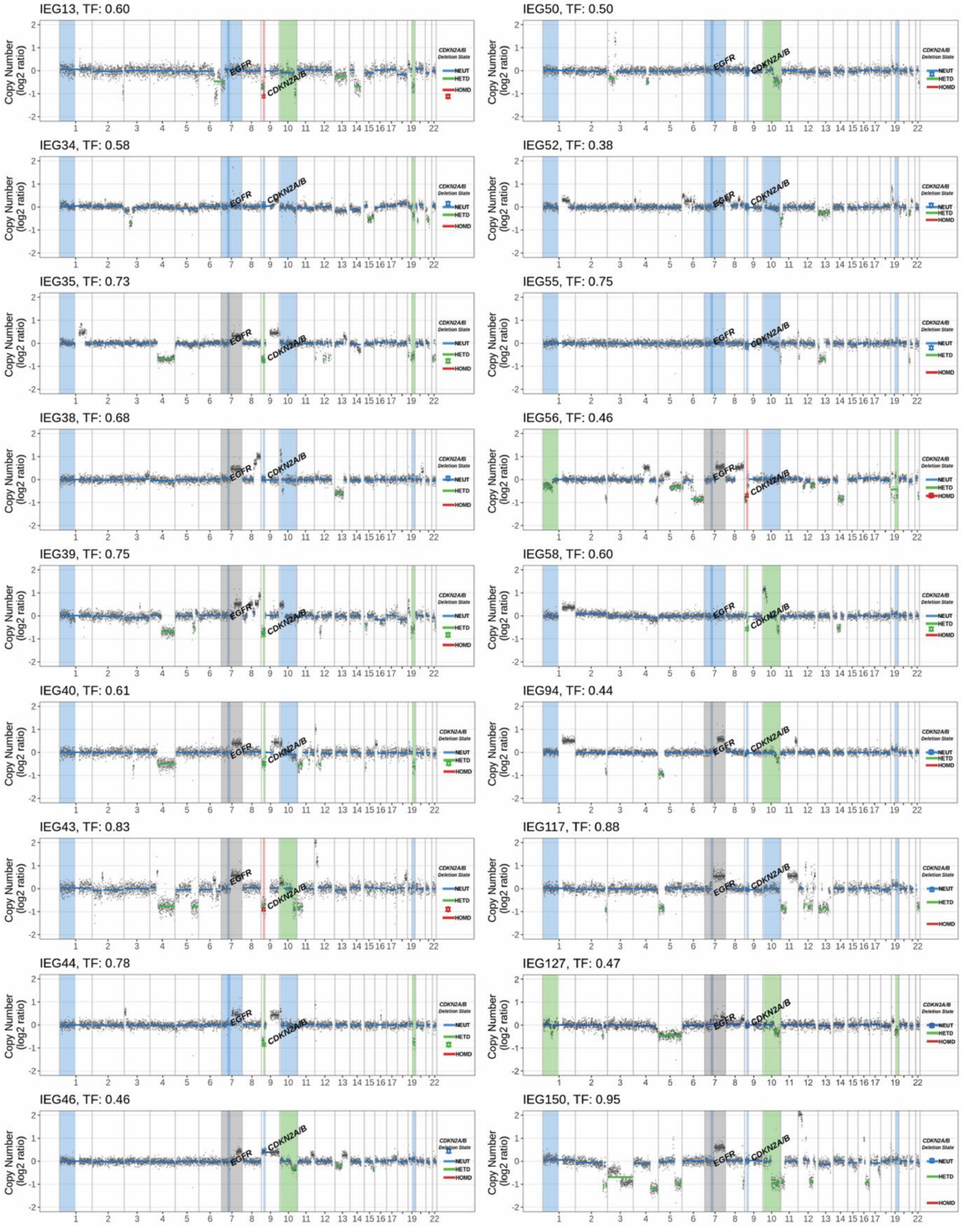

Supplementary Fig. 3: Copy number variation plots of A IDH samples obtained using GLIMMERS.

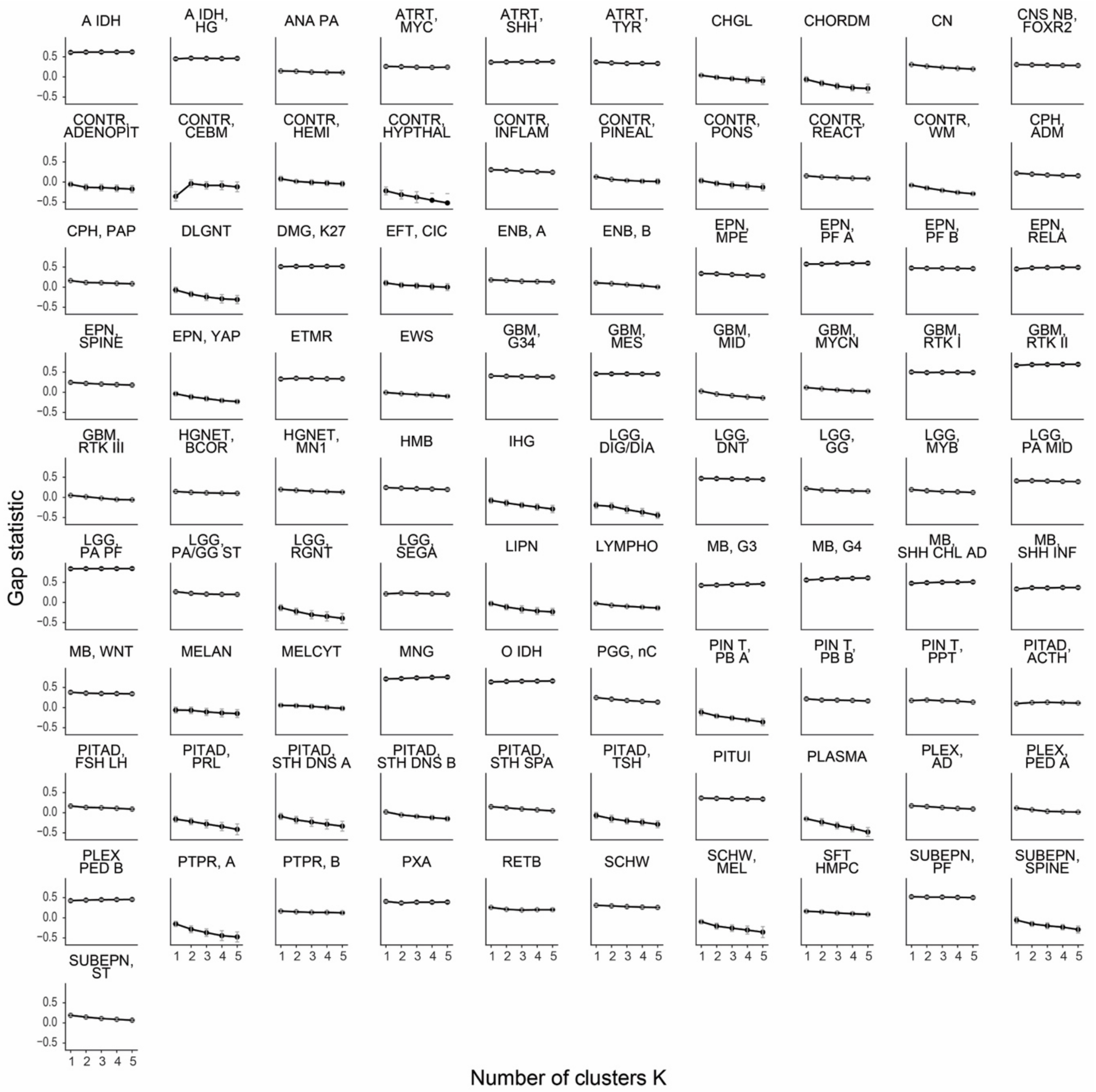

Supplementary Fig. 4: Gap statistic per CNS class as a function of the number of clusters k used for k-Means clustering on samples of the respective class. Dots indicate gap values, and error bars represent the standard error derived from B=5 Monte Carlo bootstrap samples.

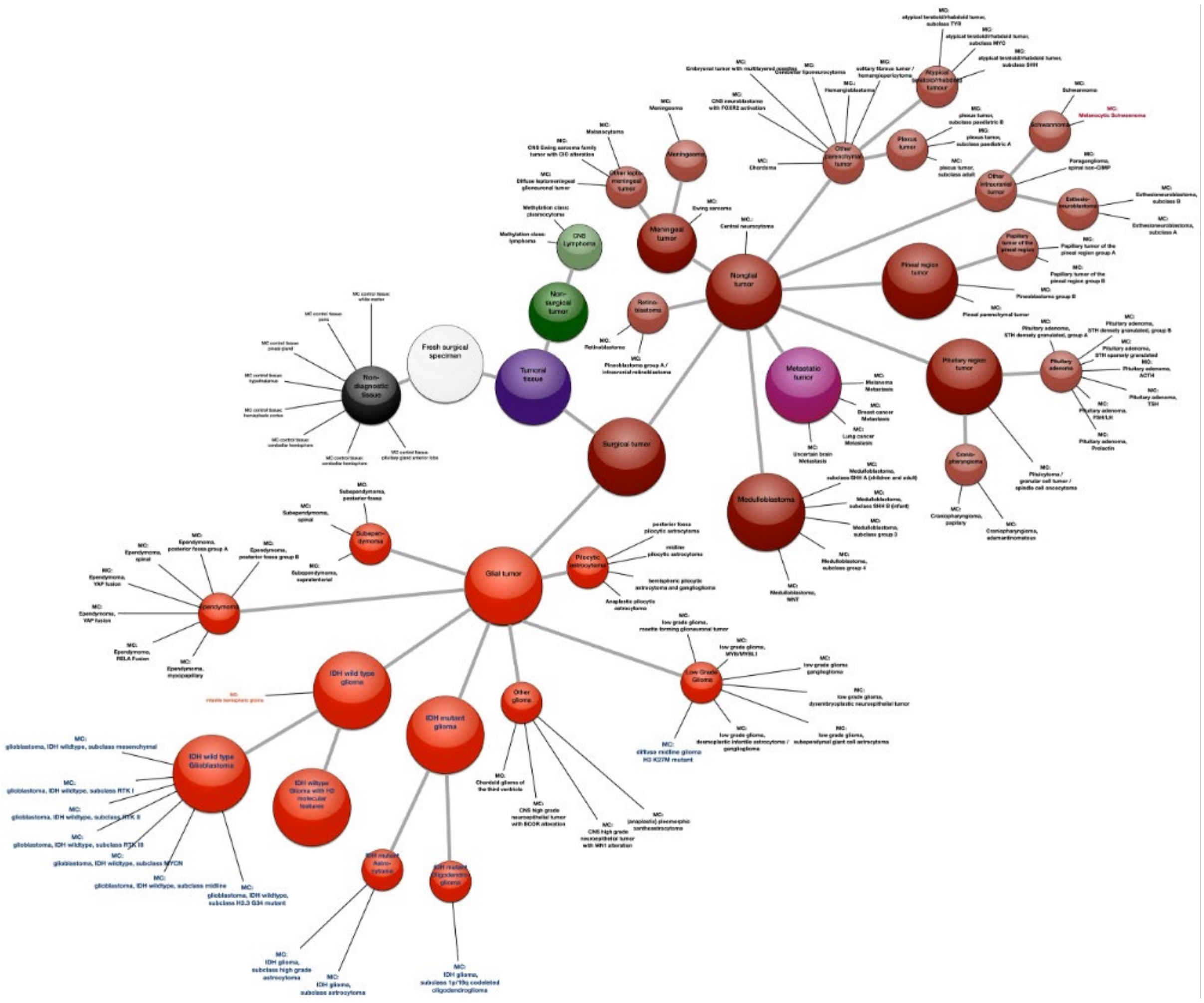

Supplementary Fig. 5: An overview of the MZ class hierarchical structure. The presented hierarchical classification system is a purely conceptual model demonstrating a potential approach to adapting a dynamic classification system to meet specific clinical and translational needs.

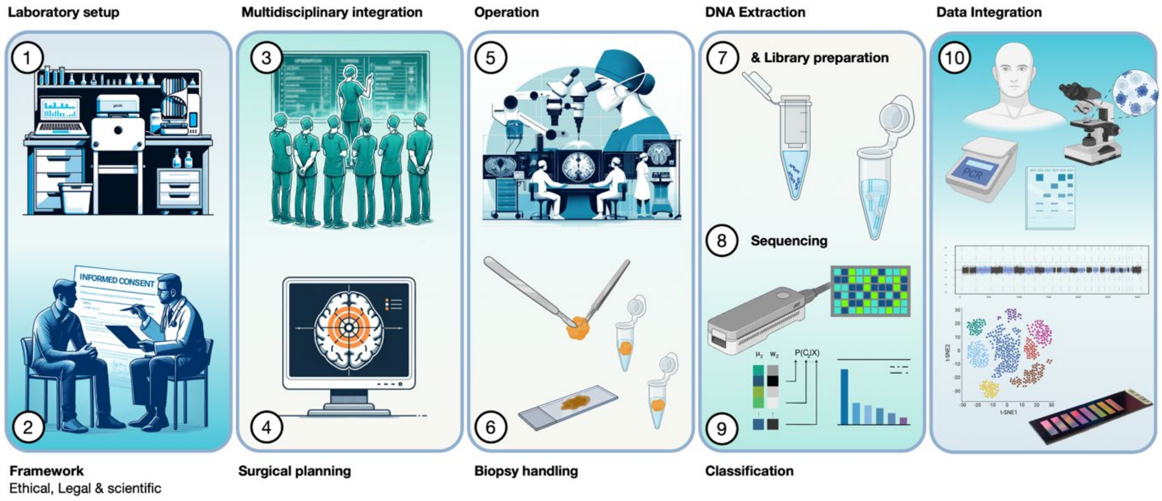

Supplementary Fig. 6: Outline of the sequential process, highlighting fundamental tenets essential for the success of such a project. This image was created with the assistance of DALL·E 2 and BioRender.

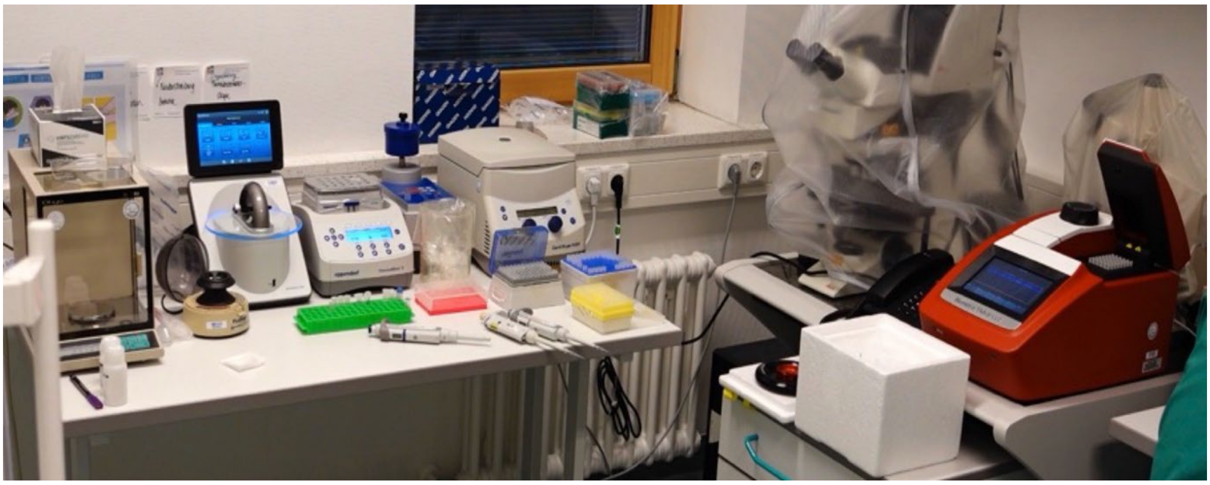

Supplementary Fig. 7: Molecular workspace: The photo showcases the exact setup used in this study and is not intended to suggest specific vendors or equipment manufacturers.

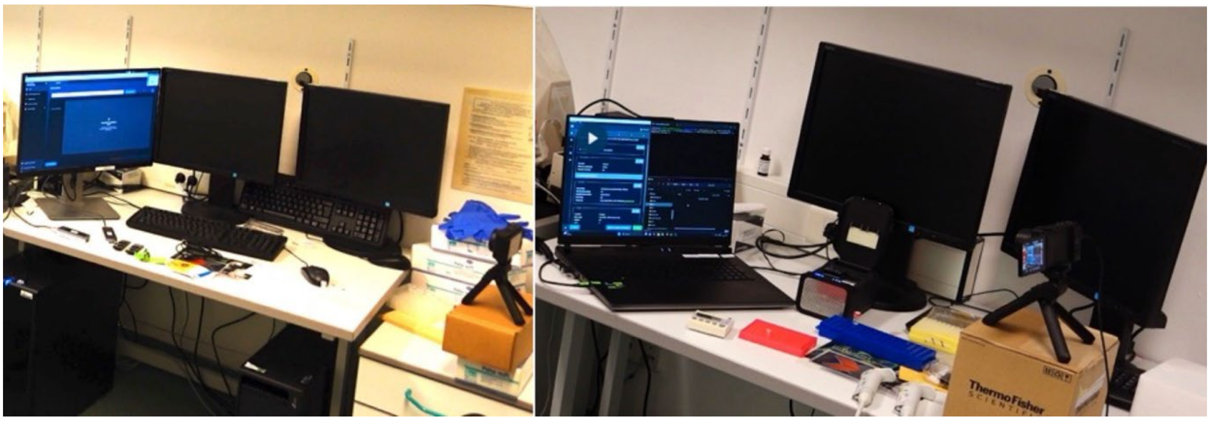

Supplementary Fig. 8: Sequencing and computer workspace.

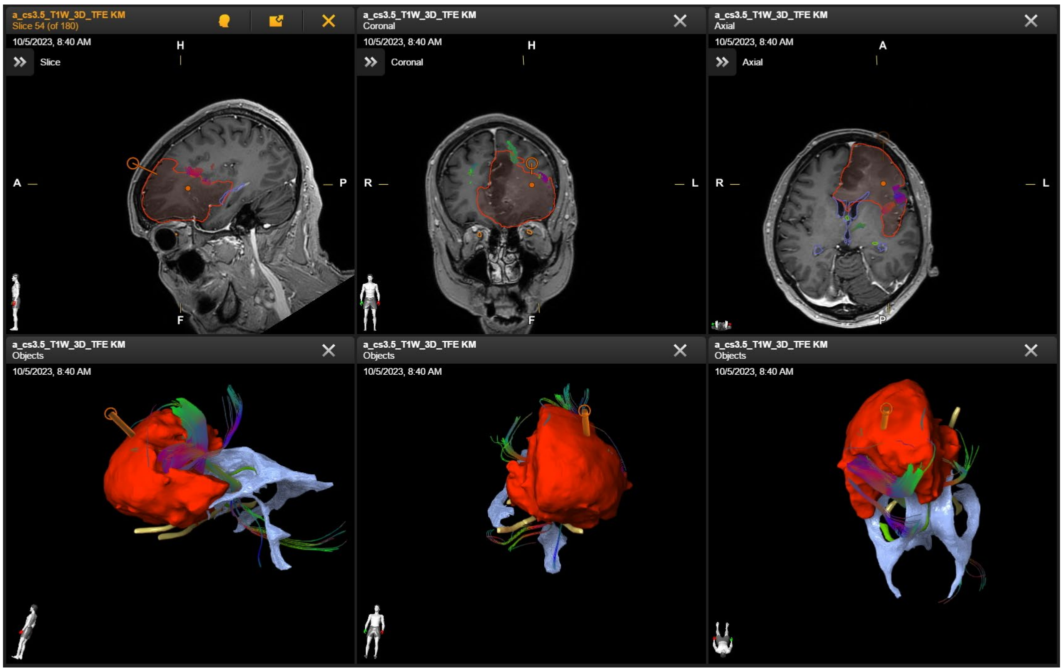

Supplementary Fig. 9: Planning of the surgical approach and biopsy collection in Brainlab. The red area represents the tumor, while the orange line indicates the trajectory for the neurosurgical approach to the brain tumor, highlighting the biopsy collection site where the trajectory intersects the tumor mass. The yellow structures represent the optic nerves, the cyan structures represent the ventricles, and the motor speech pathways (Broca's area) are delineated within the tumor. All six images are synchronized across different planes for comprehensive planning.

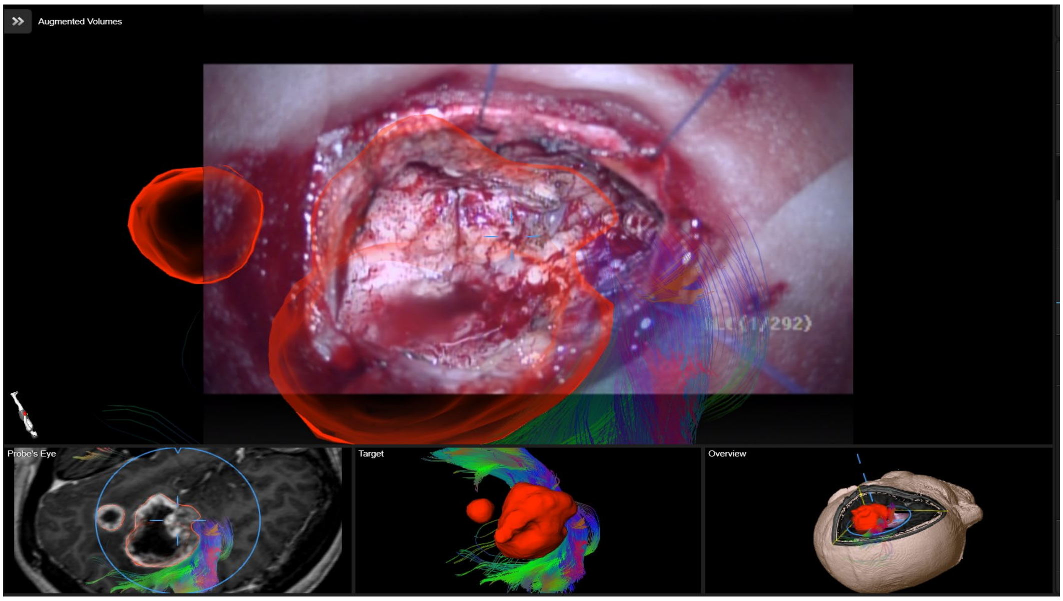

Supplementary Fig. 10: Intraoperative biopsy collection using augmented reality. The red area represents the tumor, visualized intraoperatively. The augmented reality overlay includes critical anatomical structures, such as the pyramidal tracts (visible as colored pathways). The main panel shows the real-time surgical view with the augmented tumor boundary. The lower panels provide additional perspectives: "Probe's Eye" view with the surgical trajectory targeting the tumor, a 3D target view highlighting the tumor and surrounding anatomy, and an overview of the patient's head with the surgical trajectory and tumor position. This integration of augmented reality aids in precise surgical planning and execution.

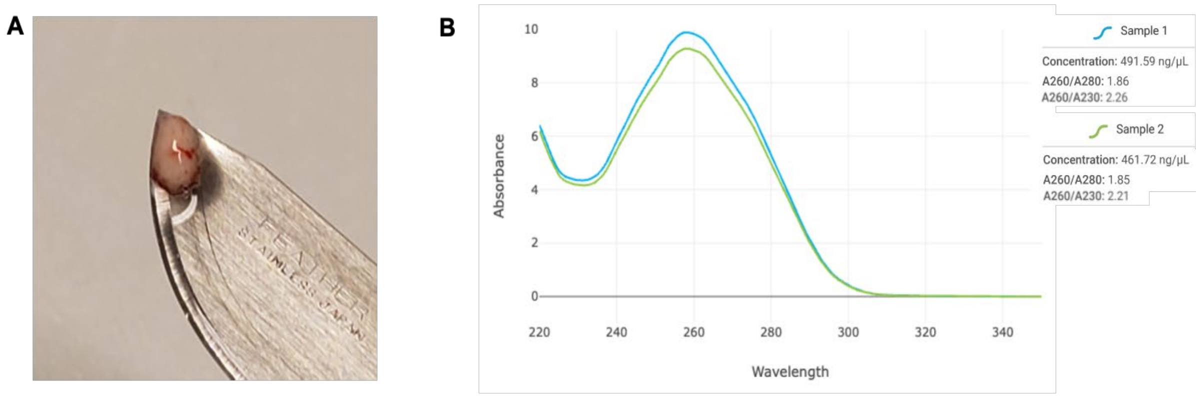

Supplementary Fig. 11: Representative images from clinical demonstrator sample IEG114 classified as Oligodendroglioma. A pinhead-sized tissue fragment of 10-15 mg. (A) enables sufficient DNA extraction using the QIAamp Fast DNA Tissue Kit, and the quality/quantity was assessed by a Nanodrop One instrument (B).

2 Supplementary Tables

Supp. Table 22: Buffer of the QIAamp Fast DNA Tissue kit that requires the addition of ethanol/isopropanol

Buffer	Supplement with	Volume
AW1	ethanol (96-100%)	25 ml
AW2	ethanol (96-100%)	40 ml
MVL	2-propanol (100%)	3.85 ml

Caution: Buffer AW1 and MVL contain a chaotropic salt and are incompatible with bleach disinfectants. Take appropriate laboratory safety measures and wear gloves when handling. Buffer AW1, AW2, and MVL are premixed with ethanol or isopropanol. Both are flammable, harmful if swallowed and cause serious eye irritation.

Supp. Table 23: Composition of the master digest buffer mix according to the QIAamp Fast DNA Tissue Kit

Reagent	MMX 1	MMX 3
Buffer AVE	200 µl	600 µl
Buffer VXL	40 µl	120 µl
Reagent DX	1 µl	3 µl
Proteinase K (600 mAU/ml)	20 µl	60 µl
RNase A (100 mg/ml)	4 µl	12 µl

Caution: Buffer AVE contains sodium azide as a preservative. Buffer VXL contains a chaotropic salt and is incompatible with bleach disinfectants. Take appropriate laboratory safety measures and wear gloves when handling.

Supp. Table 24: Components of the rapid sequencing kit (SQK-RAD114).

Reagent	Abbreviation	Cap color	Volume/rxn	Process/usage

Fragmentation mix	FRA	orange	1 µl	Provides the transposome complex for DNA tagmentation. Cleaves DNA molecules and simultaneously attaches transposase adapter to the (cleaved) DNA ends.
Adapter buffer	ADB	clear	3.5 µl	Used for proper dilution of the rapid adapter (RA)
Rapid adapter	RA	green	1.5 µl	Requires dilution with adapter buffer (ADB). The rapid adapter is attached to the tagmented DNA library and contains the motor protein vital for sequencing.
Sequencing buffer	SQB	red	100 µl	SQB is supplemented to the final sequencing library. It provides ATP to the motor protein (DNA-Helicase) and allows the proper function of the enzyme during sequencing.
Library loading beads	LIB	pink	68 µl	LIB is supplemented to the final sequencing library if DNA fragments are expected to be short, e.g., ≤ 20 kbp. Library-loading beads interact with DNA molecules and help bring them close to the nanopores.
Flow cell tether	FCT	purple	30 µl	To be used in conjunction with flow cell flush (FCF) to form the priming mix. Flow cell tether binds to the membrane of the sensor array and guides DNA molecules in close proximity to the nanopores.
Flow cell flush	FCF	blue	1170 µl	To be used in conjunction with flow cell flush (FCT) to form the priming mix. Allows removal of flow cell storage buffer.

Supp. Protocol Table 25: Preparation of the final sequencing library before loading.

Reagent	Volume
DNA library	12 µl
nuclease-free H2O	20 µl
LIB	68 µl
SQB	100 µl
Total	200 µl
