# Supplementary material for: Rapid brain tumor classification from sparse epigenomic data
Source: Nat Med. 2025 Feb 28;31(3):840–8. doi: 10.1038/s41591-024-03435-3 (PMC11922770; doi:10.1038/s41591-024-03435-3)
Supplement: Supplementary file 2 — Reporting Summary [file 41591_2024_3435_MOESM2_ESM.pdf]

nature portfolio

Corresponding author(s): Alena van Boemmel, Helene Kretzmer, Franz-Josef Müller

Last updated by author(s): Oct 14, 2024

Reporting Summary

Nature Portfolio wishes to improve the reproducibility of the work that we publish. This form provides structure for consistency and transparency in reporting. For further information on Nature Portfolio policies, see our Editorial Policies and the Editorial Policy Checklist.

Statistics

For all statistical analyses, confirm that the following items are present in the figure legend, table legend, main text, or Methods section.

n/a	Confirmed	
☐	☒	The exact sample size (*n*) for each experimental group/condition, given as a discrete number and unit of measurement
☐	☒	A statement on whether measurements were taken from distinct samples or whether the same sample was measured repeatedly
☐	☒	The statistical test(s) used AND whether they are one- or two-sided<br>*Only common tests should be described solely by name; describe more complex techniques in the Methods section.*
☐	☒	A description of all covariates tested
☐	☒	A description of any assumptions or corrections, such as tests of normality and adjustment for multiple comparisons
☐	☒	A full description of the statistical parameters including central tendency (e.g. means) or other basic estimates (e.g. regression coefficient) AND variation (e.g. standard deviation) or associated estimates of uncertainty (e.g. confidence intervals)
☐	☒	For null hypothesis testing, the test statistic (e.g. *F*, *t*, *r*) with confidence intervals, effect sizes, degrees of freedom and *P* value noted<br>*Give P values as exact values whenever suitable.*
☐	☒	For Bayesian analysis, information on the choice of priors and Markov chain Monte Carlo settings
☒	☐	For hierarchical and complex designs, identification of the appropriate level for tests and full reporting of outcomes
☐	☒	Estimates of effect sizes (e.g. Cohen's *d*, Pearson's *r*), indicating how they were calculated

Our web collection on statistics for biologists contains articles on many of the points above.

Software and code

Policy information about availability of computer code

Data collection	Minfi (version 1.32.0), MinKnow with dorado v7.0.9, minimap2 v2.24, guppy v6.2.7
Data analysis	MethyLYZR code with a pre-trained model and test data, as well as documentation, is available under the following link: https://github.com/marasteiger/MethyLYZR<br><br>Python (3.8.10) for preprocessing; R (version 4.2.2) and Python (3.7.11) for data analysis.<br>R packages: ComplexHeatmap v2.14.0, ggplot2 v3.4.3, ggsankey v0.0.99999, caret v6.0-93, cluster v2.1.4, ggpubr v0.5.0, RColorBrewer v1.1-3, arrow v10.0.1, rtracklayer v1.58.0, GenomicRanges v1.50.2, ggsci v2.9, circlize v0.4.15, tidyverse v1.3.2.<br>Python packages: arrow v1.3.0 , contourpy v1.2.0 , cycler v0.12.1, Cython v0.29.36, fonttools v4.47.2, importlib-resources v6.1.0, kiwisolver v1.4.5, mappy v2.26, matplotlib v3.8.2, memory-profiler v0.61.0, natsort v8.4.0, numpy v1.26.3, packaging v23.2, pandas v2.2.0, pillow v10.2.0, pyarrow v15.0.0, pyparsing v3.1.1, pysam v0.22.0 with samtools v1.16.1 and htslib v1.16, python-dateutil v2.8.2, pytz v2024.1, scipy 1.12.0, setuptools v44.0.0, six v1.16.0, tabulate v0.9.0, types-python-dateutil v2.8.19.20240106, tzdata v2023.4, watchdog v3.0.0, zipp v3.17.0.

For manuscripts utilizing custom algorithms or software that are central to the research but not yet described in published literature, software must be made available to editors and reviewers. We strongly encourage code deposition in a community repository (e.g. GitHub). See the Nature Portfolio guidelines for submitting code & software for further information.

Data

Policy information about availability of data

All manuscripts must include a data availability statement. This statement should provide the following information, where applicable:

- Accession codes, unique identifiers, or web links for publicly available datasets
- A description of any restrictions on data availability
- For clinical datasets or third party data, please ensure that the statement adheres to our policy

Sequencing data obtained via Nanopore or PacBio sequencing have been deposited in the European Genome–Phenome archive (study accession no. EGAS50000000559, Nanopore R9 dataset accession no. EGAD50000000832, Nanopore R10 dataset accession no. EGAD50000000791, PacBio dataset accession no. EGAD50000000798). The methylation values have been provided in feather format and have been deposited at https://zenodo.org/records/13324498. The Supplementary Video has been deposited at https://zenodo.org/records/13236097.
Previously published 450k or EPIC arrays were used for classifier training and evaluation: Brain normal and cancer data (GSE90496, GSE109379), Metastasis (GSE108576), and Sarcoma data (GSE140686). DNA methylation data for purity analysis were previously published by Djirackor et al. (2021) and were reprocessed for this study as described above. DNA methylation data for the liquid biopsy analysis were directly obtained from the authors of the original study Afflerbach et al. (2024) upon personal communication.
The reference genome GRCH38.p13 was obtained from UCSC Genome Browser.

Research involving human participants, their data, or biological material

Policy information about studies with human participants or human data. See also policy information about sex, gender (identity/presentation), and sexual orientation and race, ethnicity and racism.

Reporting on sex and gender	We report sex and age of individuals in supplementary tables. Sex was determined from information in electronic health records. Our research findings do not apply to only one sex or gender, no sex- and gender-based analyses have been performed, and sex- and gender are not relevant to our research findings.
Reporting on race, ethnicity, or other socially relevant groupings	We did not analyse race, ethnicity, or other socially relevant groupings in this study.
Population characteristics	Patients with a radiologically suspected primary brain tumor or brain metastasis undergoing surgery at the University Hospital Schleswig-Holstein Campus Kiel, Dept. of Neurosurgery, without any age restrictions, were asked to participate in the study. As the classifier was trained on a publicly available microarray dataset, only post hoc analysis of the classifier results was performed without any further patient characteristics stratification. Biopsies of Gliomas, including Glioblastomas, Astrocytomas, and Oligodendrogliomas, were sourced from archive collections at the Brain Tumor Center, University Medical Center Regensburg, and the Department of Pathology & Laboratory Medicine, Faculty of Medicine, University of British Columbia in Vancouver. Our research findings do not apply to only one sex or gender, no sex- and gender-based analyses have been performed, and sex- and gender are not relevant to our research findings.
Recruitment	Patients scheduled for an open craniotomy due to a suspected primary brain tumor or brain metastasis were consecutively identified at the Department of Neurosurgery, UKSH Campus Kiel. Patients were contacted and asked to participate. Patients initially agreeing to participate were assigned a study ID (IEGXXX). Depending on age and legal status, patients, their parents, or legal guardians signed an informed consent for using their tissue and clinical data in research (opt-in procedure). Patients who did not sign the informed consent documents, whose legal capacity to consent was unclear, or whose ability to consent was questionable due to, for example, neurocognitive deficits, were excluded from the study. However, their IEGXXX designation was retained. Samples from Vancouver and Regensburg were assigned a consecutive IEGXXX number on the day they were sequenced.
Ethics oversight	The study protocol was approved by and adhered to the Clinical Ethics Committee of the Medical Faculty of Kiel University (D443/20). All included patients, or their legal guardians/parents provided written informed consent for participation in the study. The results were not shared with treating physicians or caregivers and, therefore, not used to alter patient treatment or diagnosis. The study was approved by and adhered to the University of British Columbia Research Ethics Committee, Vancouver, BC, Canada (REB# H08-02838). The study was approved by and adhered to the Clinical Ethics Committee of the Medical Faculty of Regensburg University (20-1799-101).

Note that full information on the approval of the study protocol must also be provided in the manuscript.

Field-specific reporting

Please select the one below that is the best fit for your research. If you are not sure, read the appropriate sections before making your selection.

☒ Life sciences ☐ Behavioural & social sciences ☐ Ecological, evolutionary & environmental sciences

For a reference copy of the document with all sections, see nature.com/documents/nr-reporting-summary-flat.pdf

Life sciences study design

All studies must disclose on these points even when the disclosure is negative.

Sample size	No statistical methods were used to predetermine sample sizes, but were determined based on availability. Further clinical validation is pending, however the low incidence of CNS tumors and the high number of different classes prevents collection of extensive cohorts in a timely manner. Sample sizes are indicated in the figure panels or legends.
Data exclusions	Patients who did not sign the informed consent documents, whose legal capacity to consent was unclear, or whose ability to consent was questionable due to, for example, neurocognitive deficits, were excluded from the study.
Replication	Tests on 450k methylation arrays were performed on 100 synthetic replicates from the same sample (average prediction accuracy >95%). Tumor classes followed in replication number the distribution as obtained in the clinics (ONT sequencing), with 1-6 replicates per sample. All technical replication attempts were successful, biological replication; ie sequencing from different samples of the same tumor showed some variability, likely due to sample purity and covered genomic regions.
Randomization	Our genomic analyses are independent of human intervention and analyze each sample equally and in an unbiased fashion.
Blinding	Blinding was not relevant for this study since this is not an intervention study. However, our analytical pipeline followed uniform criteria applied to all samples, allowing us to analyze our data in an unbiased manner.

Reporting for specific materials, systems and methods

We require information from authors about some types of materials, experimental systems and methods used in many studies. Here, indicate whether each material, system or method listed is relevant to your study. If you are not sure if a list item applies to your research, read the appropriate section before selecting a response.

Materials & experimental systems

n/a	Involved in the study
☒	☐ Antibodies
☒	☐ Eukaryotic cell lines
☒	☐ Palaeontology and archaeology
☒	☐ Animals and other organisms
☒	☐ Clinical data
☒	☐ Dual use research of concern
☒	☐ Plants

Methods

n/a	Involved in the study
☒	☐ ChIP-seq
☒	☐ Flow cytometry
☒	☐ MRI-based neuroimaging

Plants

Seed stocks	*Report on the source of all seed stocks or other plant material used. If applicable, state the seed stock centre and catalogue number. If plant specimens were collected from the field, describe the collection location, date and sampling procedures.*
Novel plant genotypes	*Describe the methods by which all novel plant genotypes were produced. This includes those generated by transgenic approaches, gene editing, chemical/radiation-based mutagenesis and hybridization. For transgenic lines, describe the transformation method, the number of independent lines analyzed and the generation upon which experiments were performed. For gene-edited lines, describe the editor used, the endogenous sequence targeted for editing, the targeting guide RNA sequence (if applicable) and how the editor was applied.*
Authentication	*Describe any authentication procedures for each seed stock used or novel genotype generated. Describe any experiments used to assess the effect of a mutation and, where applicable, how potential secondary effects (e.g. second site T-DNA insertions, mosiacism, off-target gene editing) were examined.*
